# Hidden Flaws Behind Expert-Level Accuracy of GPT-4 Vision in Medicine

**Published:** 2024-01-24

**Authors:** Qiao Jin, Fangyuan Chen, Yiliang Zhou, Ziyang Xu, Justin M. Cheung, Robert Chen, Ronald M. Summers, Justin F. Rousseau, Peiyun Ni, Marc J Landsman, Sally L. Baxter, Subhi J. Al’Aref, Yijia Li, Michael F. Chiang, Yifan Peng, Zhiyong Lu

**Affiliations:** 1 National Library of Medicine, National Institutes of Health, Bethesda, MD, USA.; 2 University of Pittsburgh, Pittsburgh, PA, USA.; 3 Department of Population Health Sciences, Weill Cornell Medicine, New York, NY, USA.; 4 Ronald O. Perelman Department of Dermatology, New York University Grossman School of Medicine, New York City, NY, USA.; 5 Department of Medicine, Harvard Medical School and Massachusetts General Hospital, Boston, MA, USA.; 6 Pathology & Laboratory Medicine, Weill Cornell Medicine, New York, NY, USA.; 7 Imaging Biomarkers and Computer-Aided Diagnosis Laboratory, Department of Radiology and Imaging Sciences, National Institutes of Health Clinical Center, Bethesda, MD, USA.; 8 Department of Neurology, Peter O’Donnell Jr. Brain Institute, UT Southwestern Medical Center, Dallas, TX, USA.; 9 Division of Gastroenterology, Department of Medicine, Harvard Medical School and Massachusetts General Hospital, Boston, MA, USA.; 10 Division of Gastroenterology, Department of Medicine, Metrohealth Medical Center, Cleveland, OH, USA. Case Western Reserve University School of Medicine, Cleveland, OH, USA.; 11 Division of Ophthalmology Informatics and Data Science, Viterbi Family Department of Ophthalmology and Shiley Eye Institute, University of California San Diego, La Jolla, CA, USA.; 12 Division of Cardiology, Department of Internal Medicine, University of Arkansas for Medical Sciences, Little Rock, AR, USA.; 13 University of Pittsburgh Medical Center, Pittsburgh, PA, USA.; 14 National Eye Institute, National Institutes of Health, Bethesda, MD, USA.

## Abstract

Recent studies indicate that Generative Pre-trained Transformer 4 with Vision (GPT-4V) outperforms human physicians in medical challenge tasks. However, these evaluations primarily focused on the accuracy of multi-choice questions alone. Our study extends the current scope by conducting a comprehensive analysis of GPT-4V’s rationales of image comprehension, recall of medical knowledge, and step-by-step multimodal reasoning when solving *New England Journal of Medicine* (NEJM) Image Challenges – an imaging quiz designed to test the knowledge and diagnostic capabilities of medical professionals. Evaluation results confirmed that GPT-4V outperforms human physicians regarding multi-choice accuracy (88.0% vs. 77.0%, p=0.034). GPT-4V also performs well in cases where physicians incorrectly answer, with over 80% accuracy. However, we discovered that GPT-4V frequently presents flawed rationales in cases where it makes the correct final choices (27.3%), most prominent in image comprehension (21.6%). Regardless of GPT-4V’s high accuracy in multi-choice questions, our findings emphasize the necessity for further in-depth evaluations of its rationales before integrating such models into clinical workflows.

Large language models (LLMs) exemplified by Generative Pre-trained Transformer 4 (GPT-4)^1^ have achieved remarkable performance on a wide range of biomedical tasks^2^, including answering medical license examination questions^3,4^, assisting literature search^5^, matching patients to clinical trials^6^, and summarizing medical evidence^7^. However, most of these LLMs are unimodal, utilizing only the context in natural language, while clinical tasks most often require the integration of narrative clinical descriptions and multiple types of imaging tests^8,9^. Recently, OpenAI released GPT-4 with Vision (GPT-4V), a state-of-the-art multimodal LLM that allows users to instruct GPT-4 to analyze both images and texts together. Subsequent pilot studies have been conducted to analyze the performance of GPT-4V in the medical domain^10–12^ (summarized in Supplementary Table 1). These evaluations mainly focused on the accuracy of GPT-4V in answering multi-choice medical questions, and in some cases, GPT-4V outperformed medical students and even physicians in closed-book settings. However, the multi-choice accuracy might not reflect the actual competence of GPT-4V, and there is no guarantee that correct final choices are based on accurate underlying rationales. Therefore, a thorough analysis is imperative to assess whether the decision-making of GPT-4V is based on sound rationales, rather than arbitrary conjecture.

To bridge this gap, we used 100 multiple-choice questions with single correct answers from *New England Journal of Medicine* (NEJM) Image Challenge (between November 11, 2021, and October 5, 2023) as this task is non-trivial (see details with a prior vision-language foundation model in Methods). Specifically, we concentrated on evaluating the GPT-4V model, assessing its proficiency in generating both the final answer and the rationales with respect to three essential capabilities – (1) **Image comprehension**, where the model describes the provided patient image(s); (2) **Recall of medical knowledge**, where the model generates relevant medical knowledge required to solve the question, such as outlining the radiological characteristics associated with each possible choice; and (3) **Step-by-step reasoning**, where the model demonstrates detailed multimodal reasoning to answer the given question, utilizing the generated content from both image comprehension and recall of medical knowledge. The Institutional Review Board (IRB) has determined that this study does not involve human subjects. Therefore, IRB review and approval are not required.

Fig. 1 presents the overall design of this study. A senior medical student collected and answered the questions, establishing a student baseline. We then used a specifically designed prompt to ask GPT-4V to generate rationales in separate sections, which facilitates easier localization of the involved capability (described in Online Methods). GPT-4V responses were manually recorded in independent chat sessions. Each question in the dataset was then categorized into a medical specialty and was annotated by one clinician in that field. A multidisciplinary cohort of nine attending and resident physicians from different specialties was recruited to answer the questions and evaluate the rationales of GPT-4V based on their expertise, with reference to the official correct answers and explanations provided by NEJM Image Challenge.

The evaluation results are shown in Fig. 2. Human performance is evaluated in two settings: the closed-book setting (without using external tools such as a literature search engine or the Internet), and the open-book setting (with external resources). GPT-4V can be considered using a closed-book setting because web browsing is disabled. First and foremost, GPT-4V surpassed physicians in closed-book setting per final choice selection (Fig. 2a). GPT-4V achieved 88% overall accuracy (CI: 82–94%), significantly higher than that of physicians (77%, CI: 62–85%). GPT-4V also largely outperforms the senior medical student, who achieved an average accuracy of 60% (CI: 50–70%) under the closed-book setting, representing a human passing score. The best performance under the open-book setting is achieved by human physicians (95%, CI: 91–99%), though not significantly different from GPT-4V. Our findings, therefore, align with the previous ones, which show the superior performance of GPT-4V in the closed-book setting^12,13^.

To investigate performance in relation to question difficulty, we classified the questions into three levels containing similar numbers of questions based on the percentage of correct answers chosen by the users from the NEJM website – easy (33 questions), medium (34 questions), and hard (33 questions). Overall performance correlates with question difficulty – almost all respondent groups showed non-inferior performance in easy questions compared to the other two levels. Differences between the studied groups are not significant for easy questions. For the medium-level questions, GPT-4V significantly outperforms all human groups in the closed-book setting, but there is no significant difference between the performance of GPT-4V and the open-book human physicians. Interestingly, for hard questions, human physicians with the open-book setting achieved a significantly higher score than GPT-4V, suggesting the assistance of GPT-4V to physicians in complex clinical scenarios could be fairly limited.

Fig. 2b displays the confusion matrices of GPT-4V and human physicians. Overall, 19 out of 23 (82.6%) questions that physicians failed to answer in the closed-book setting were correctly answered by GPT-4V. Similarly, 4 out of 5 (80.0%) questions incorrectly answered by physicians in the open-book setting were correctly answered by GPT-4V. This suggests that GPT-4V holds potential in decision support for physicians, particularly in closed-book scenarios. Such potential utility can be illustrated via Question 96 (Supplementary Data), which all human groups answered incorrectly. In comparison, GPT-4V successfully deduced tongue ulceration as a rare complication in the context of other manifestations of giant cell arteritis. Overall, only 1 out of 100 questions was answered incorrectly by both physicians (open-book) and GPT-4V, indicating a promising synergy between the current open-book tools and GPT-4V.

We next evaluated the rationales of GPT-4V in three dimensions – image comprehension, recall of medical knowledge, and step-by-step reasoning (Fig. 2c). We found that image comprehension is the most problematic, with more than 20% of cases (21.3%–29.4% in different difficulty levels) containing flawed rationales. For example, GPT-4V mistakenly counted the input image containing three CT images, while there are only two provided in Question 12 (Supplementary Data). In contrast, medical knowledge recall is the most reliable, with error rates ranging from 12.1% to 15.2%. Step-by-step reasoning has an intermediate error rate (19.2%–21.2%). Across all types of rationales, GPT-4V reached accurate rationales in the majority (70.6%–87.9%) of all difficulty groups.

Surprisingly, despite overall satisfactory performance, a closer investigation showed that GPT-4V can still be erroneous in one or multiple rationales when the final answer is correct – these mistakes predominantly occur in image comprehension (21.6%), as opposed to knowledge recall (10.2%) and reasoning (11.4%). For instance, in image comprehension of Question 21 (Supplementary Data), GPT-4V correctly identified malignant syphilis with multiple evidence, but it failed to recognize that the two skin lesions presenting at different angles actually arise from the same pathology. In another case of Question 89 (Supplementary Data), GPT-4V correctly linked Lisch nodules with features of the iris surface; however, medical knowledge recall of other options was mistaken – e.g., it wrongly stated that iris nodule is uncommon in sarcoid-associated uveitis. GPT-4V could also be logically incomplete while guessing right – in Question 95 (Supplementary Data), it failed to exclude Argyll Roberson pupil with a sound reason, a condition which also presents with light-near dissociation (the correct answer) but has drastically different etiology than the truth. This showed its incompetence to distinguish similar manifestations. This finding is consistent with the design of the multi-choice question format, as one does not need to understand all related knowledge to predict the correct choice. However, a solid and comprehensive understanding of real-world cases is expected from clinicians, which is crucial in making valid real-world decisions.

Our evaluation has several limitations. First, we studied a relatively small number of 100 questions as each GPT-4V output requires human examination which is costly and time consuming. Like other similar studies, we use challenge questions with single correct answers. However, clinicians routinely encounter cases with incomplete information, where multiple diagnoses are possible. This requires listing rationales for each differential diagnosis with supportive or excluding evidence, and proposals of further testing or treatment. In future studies, we plan to also evaluate the rationales of human physicians in answering medical questions for comparison.

In summary, we present a comprehensive evaluation of GPT-4V’s rationales in multimodal medical challenge tasks. Although GPT-4V demonstrates superior multi-choice accuracy compared to human physicians in closed-book settings, physicians remain superior with open-book tools, especially in hard questions. Moreover, among correctly answered questions, GPT-4V may fail to understand or interpret medical scenarios correctly at individual rationales. Our research also identified image comprehension as the greatest challenge for GPT-4V, with an error rate of over 20%, while medical knowledge recall was the most reliable. This suggests that comprehensive evaluations beyond mere multi-choice accuracy are needed before these models can be integrated into clinical practices.

## Online Methods

### Collecting NEJM Image Challenge

For the collection of NEJM Image Challenges, we assembled 100 most recent questions (between November 11, 2021, and October 5, 2023) along with their ground-truth explanations and answers at https://www.nejm.org/image-challenge. The proportion of correct answers from NEJM users, which varied between 31% and 83%, was employed to indicate question difficulty. Consequently, the challenges were categorized into three difficulty tiers: “easy” for a 57%–83% correct answer rate, “medium” for 44%–57%, and “hard” for 31%–44%. The medical specialty and imaging modality distributions are shown in Supplementary Fig. 1.

### Prompting GPT-4V

We used the Web version of GPT-4V on 10/05/2023 through https://chat.openai.com/, the pre-training of which was completed in 2022. We observed consistent performance, with no significant difference between 2023 and earlier questions (2021–2022), indicating minimal impact from potential dataset leakage. The prompt we used to evaluate GPT-4V is shown below:

{*image*}

{*question*}

{*choices*}

Please first describe the image in a section named ”Image comprehension”.

Then, recall relevant medical knowledge that is useful for answering the question but is not explicitly mentioned in a section named ”Recall of medical knowledge”.

Finally, based on the first two sections, provide your step-by-step reasoning and answer the question in a section named ”Step-by-step reasoning”.

Please be concise.

Here {*image*}, {*question*}, and {*choices*} represent the actual image, question, and the set of possible answers for each NEJM Image Challenge, respectively.

### BiomedCLIP

To assess the difficulty of the NEJM Image Challenge for vision-language foundation models, we tested the performance of BiomedCLIP^14^, a multimodal LLM that is contrastively pre-trained on a dataset of 15 million figure-caption pairs extracted from biomedical literature. We used BiomedCLIP in a zero-shot setting to predict the correct choice for each question. Specifically, let $E_{i}$ be the pre-trained image encoder and $E_{t}$ be the pre-trained text encoder. Both the image and the text encoders are accessed from Hugging Face via https://huggingface.co/microsoft/BiomedCLIP-PubMedBERT_256-vit_base_patch16_224. Each NEJM Image Challenge contains an image I and five free-text choices $C_{1},\ldots ,C_{5}$. We first generated the embeddings of the image and all choices with their corresponding encoders, and then computed the logit for each choice by its dot product with the image representation:

(1) $$Logit\left(C_{i}\right)=E_{i}(I{)}^{T}E_{t}\left(C_{i}\right)\in R$$

The choice with the highest logit will be the predicted answer by BiomedCLIP.


(2) $$\overset{ˆ}{i}=Logit\left(C_{i}\right)$$

Overall, BiomedCLIP achieved the lowest performance of 26% accuracy (CI: 17–35%), only slightly better than chance (20%). This suggests the difficulty of the NEJM Image Challenge for vision-language foundation models of smaller sizes.

### Annotations on MTurk

The challenge questions are first triaged into nine medical specialties, including dermatology (34 cases), pathology (10 cases), pulmonology (8 cases), gastroenterology (7 cases), neurology (7 cases), ophthalmology (6 cases), cardiology (6 cases), infectious diseases (5 cases), and other internal medicine (17 cases). A senior medical student provided a closed-book answer for each question as the student baseline. For each specialty, a resident or attending physician was recruited to perform a two-stage annotation. In the initial stage, the physicians were asked to answer the questions of their specialty both without (closed-book) and with (open-book) the use of external resources such as internet searches.

In the second stage of annotation, the human physicians review GPT-4V’s responses given the ground-truth explanations and answers provided by the NEJM website, evaluating the presence of errors within each segment of GPT-4V’s rationale (Image Comprehension, Recall of Medical Knowledge, Step-by-step Reasoning) and the accuracy of GPT-4V’s final answers. GPT-4V’s rationale for each capability is labeled as either “Correct”, “Partially Correct”, or “Incorrect”. When “Partially Correct” or “Incorrect” are chosen, the physicians are also required to explain the reasons. Both stages of annotations were conducted on the Amazon Mechanical Turk (MTurk) platform at https://workersandbox.mturk.com/. The annotation interfaces of the first and second stages of annotations are shown in Supplementary Figures 2 and 3, respectively. The full annotation reports are shown in the Supplementary Data.

### Related work

The related studies on evaluating GPT-4V are summarized in Supplementary Table 1 and are compared to this study. Importantly, our evaluation is the only one that includes both quantitative performance with physicians and systematic rationale evaluations for all answers generated by GPT-4V.

## Supplementary Material

Supplement 1

## Figures and Tables

**Figure 1:**
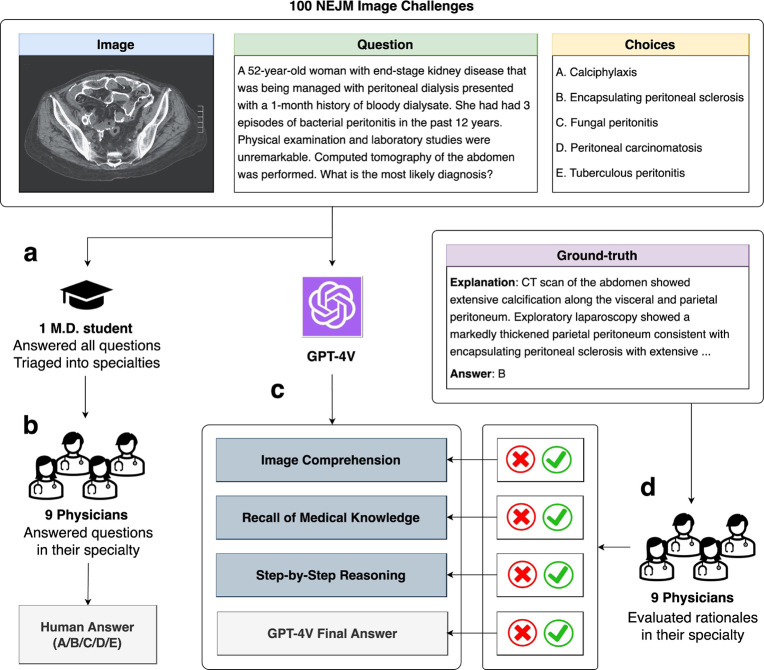
Evaluation Procedure for GPT-4 with Vision (GPT-4V). This figure illustrates the evaluation workflow for GPT-4V using 100 NEJM Image Challenges collected between November 11, 2021, and October 5, 2023. **a**, A senior medical student answered all questions and triaged them into specialties. **b**, A cohort of nine resident and attending physicians provided their answers to the questions in their specialty. **c**, GPT-4V is prompted to answer challenge questions with a final choice and structured responses reflecting three specific capabilities (Image Comprehension, Recall of Medical Knowledge, and Step-by-Step Reasoning). **d**, The human physicians then appraised the validity of each component of GPT-4V’s responses based on the ground-truth explanations.

**Figure 2:**
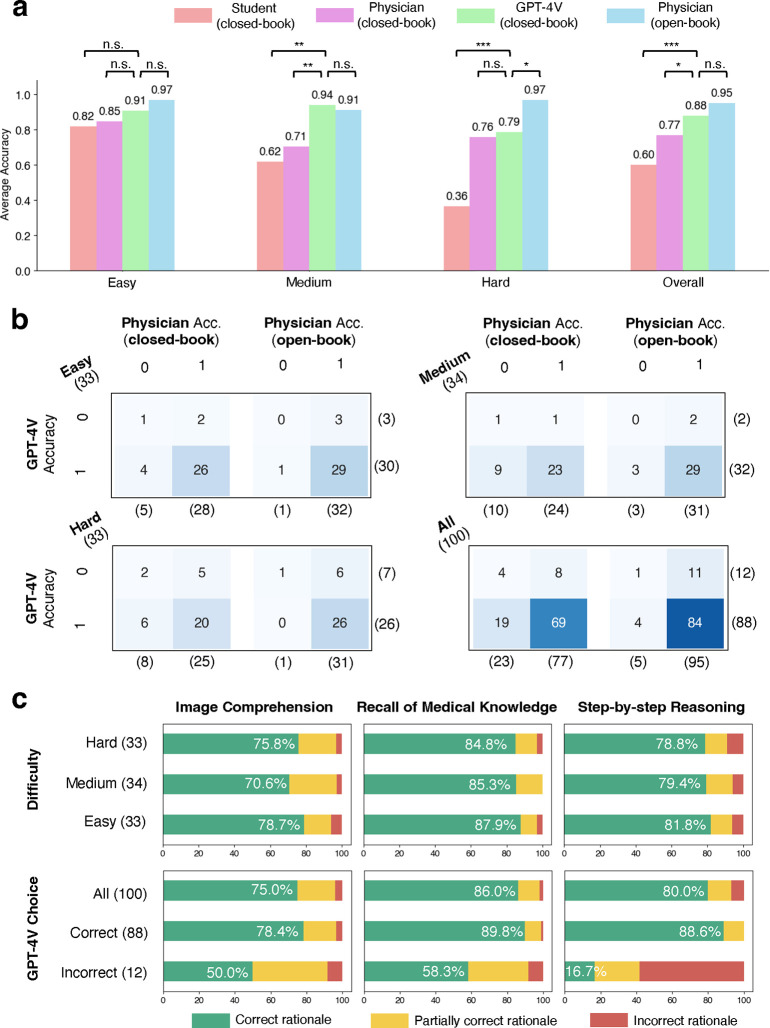
Evaluation results. **a**, Average multi-choice accuracies achieved by various models and individuals, segmented by question difficulty. Statistical significance is computed by two-sided paired t-test. **b**, Confusion matrices showing the intersection of errors made by GPT-4V and human physicians. **c**, Bar graphs representing the percentage of GPT-4V’s rationales in each capability area as evaluated by human physicians for accuracy. *: *p* < 0.05, **: *p* < 0.01, ***: *p* < 0.001, n.s.: not significant.
