# Supplementary material for: Hidden Flaws Behind Expert-Level Accuracy of Multimodal GPT-4 Vision in Medicine
Source: ArXiv. 2024 Apr 22:arXiv:2401.08396v3. Preprint. [Version 3] (PMC10896362)
Supplement: Supplementary file 1 [file NIHPP2401.08396v3-supplement-1.pdf]

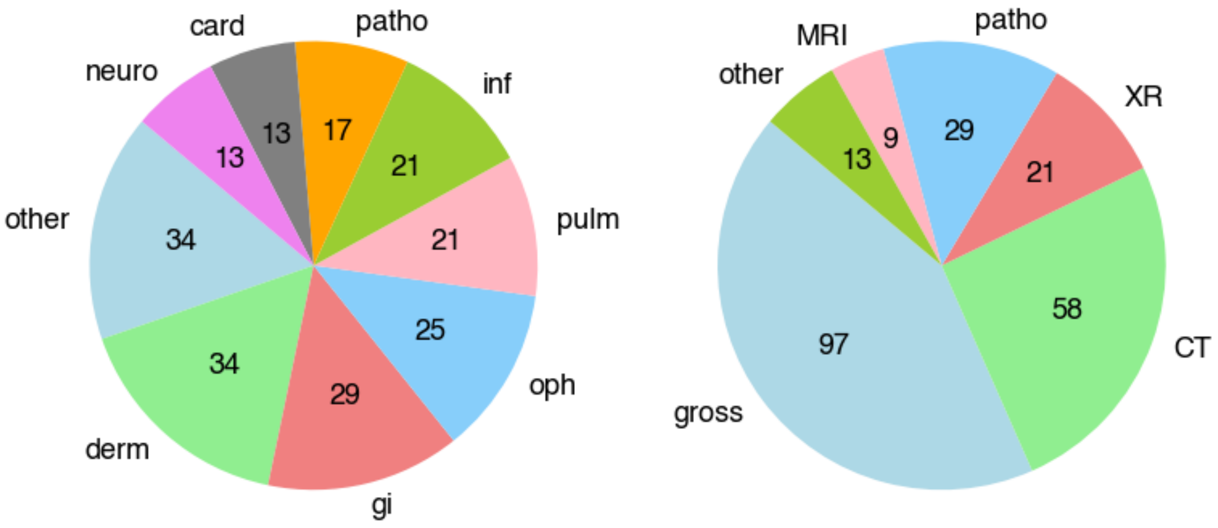

Supplementary Fig. 1. The medical specialty and imaging modality distributions of the 207 NEJM Image Challenges used in this study. (derm: dermatology, other: other internal medicine, pulm: pulmonology, neuro: neurology, gi: gastroenterology, oph: ophthalmology, card: cardiology, inf: infectious diseases; gross: macroscopic clinical images, patho: microscopic pathology, CT: computed tomography, XR: X-ray, MRI: magnetic resonance imaging; other: other image types including electrocardiogram, echocardiogram, etc.).

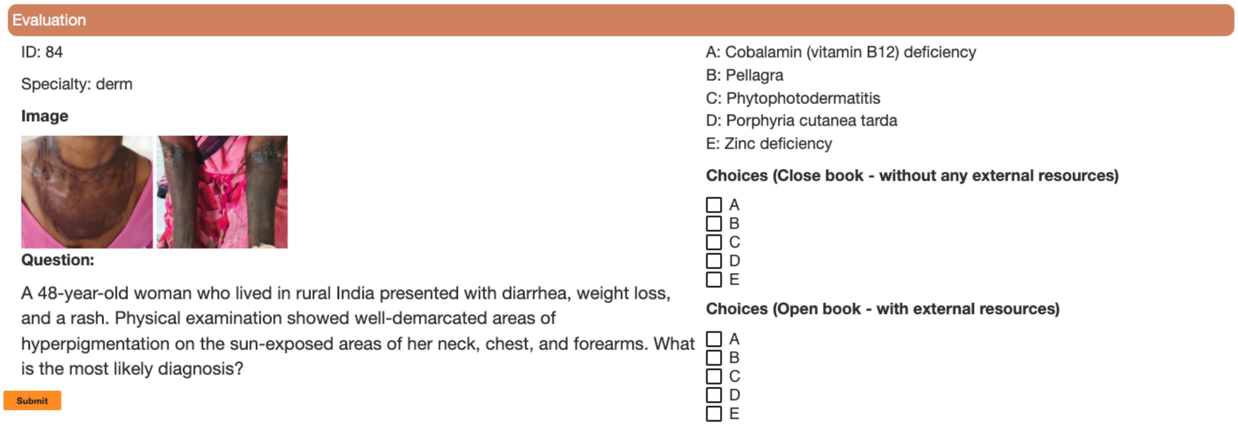

Supplementary Fig. 2. Annotation interface for human physicians to provide their closed-book and open-book answers to NEJM Image Challenge.

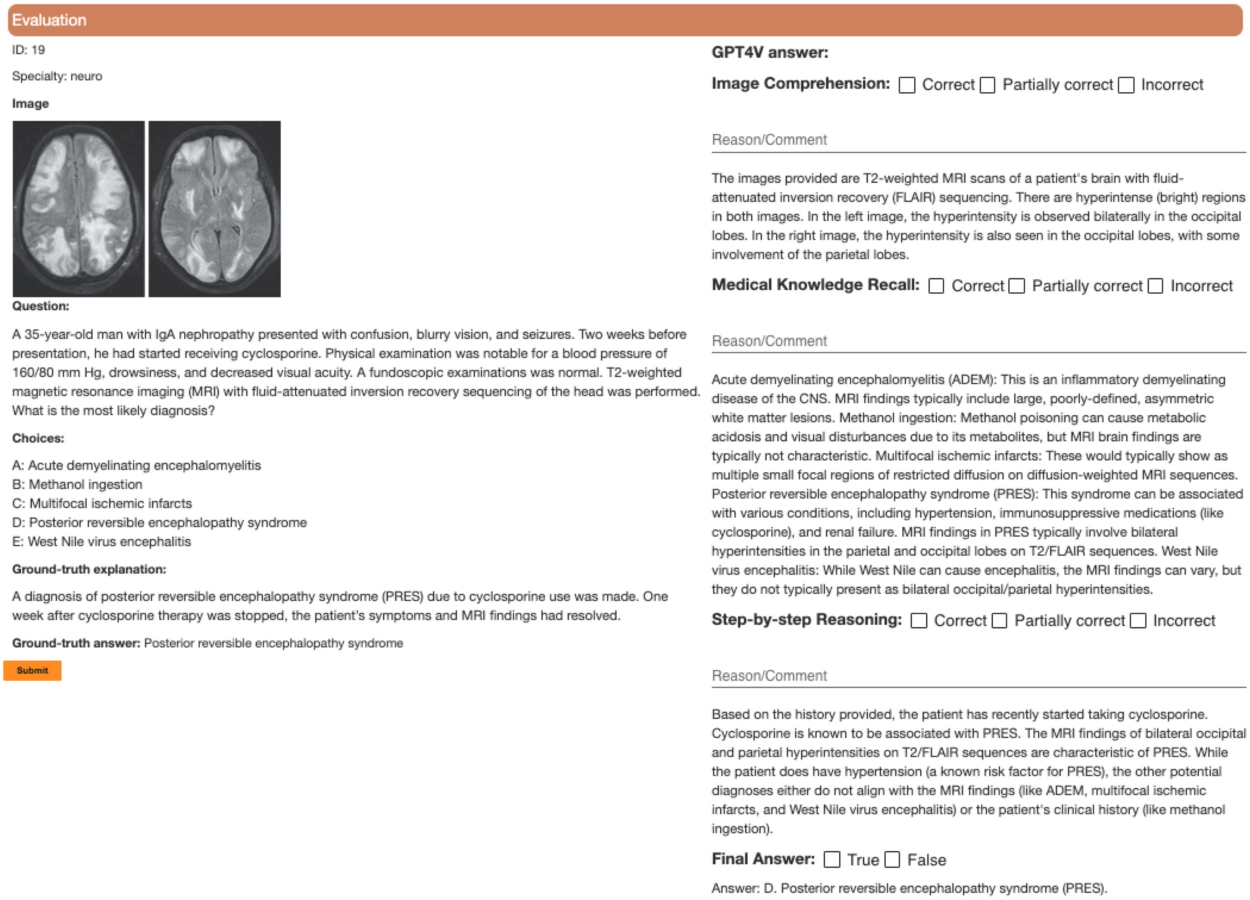

Evaluation

ID: 19

Specialty: neuro

Image

Question:

A 35-year-old man with IgA nephropathy presented with confusion, blurry vision, and seizures. Two weeks before presentation, he had started receiving cyclosporine. Physical examination was notable for a blood pressure of 160/80 mm Hg, drowsiness, and decreased visual acuity. A fundoscopic examinations was normal. T2-weighted magnetic resonance imaging (MRI) with fluid-attenuated inversion recovery sequencing of the head was performed. What is the most likely diagnosis?

Choices:

A: Acute demyelinating encephalomyelitis
B: Methanol ingestion
C: Multifocal ischemic infarcts
D: Posterior reversible encephalopathy syndrome
E: West Nile virus encephalitis

Ground-truth explanation:

A diagnosis of posterior reversible encephalopathy syndrome (PRES) due to cyclosporine use was made. One week after cyclosporine therapy was stopped, the patient's symptoms and MRI findings had resolved.

Ground-truth answer: Posterior reversible encephalopathy syndrome

Submit

GPT4V answer:

Image Comprehension: ☐ Correct ☐ Partially correct ☐ Incorrect

Reason/Comment

The images provided are T2-weighted MRI scans of a patient's brain with fluid-attenuated inversion recovery (FLAIR) sequencing. There are hyperintense (bright) regions in both images. In the left image, the hyperintensity is observed bilaterally in the occipital lobes. In the right image, the hyperintensity is also seen in the occipital lobes, with some involvement of the parietal lobes.

Medical Knowledge Recall: ☐ Correct ☐ Partially correct ☐ Incorrect

Reason/Comment

Acute demyelinating encephalomyelitis (ADEM): This is an inflammatory demyelinating disease of the CNS. MRI findings typically include large, poorly-defined, asymmetric white matter lesions. Methanol ingestion: Methanol poisoning can cause metabolic acidosis and visual disturbances due to its metabolites, but MRI brain findings are typically not characteristic. Multifocal ischemic infarcts: These would typically show as multiple small focal regions of restricted diffusion on diffusion-weighted MRI sequences. Posterior reversible encephalopathy syndrome (PRES): This syndrome can be associated with various conditions, including hypertension, immunosuppressive medications (like cyclosporine), and renal failure. MRI findings in PRES typically involve bilateral hyperintensities in the parietal and occipital lobes on T2/FLAIR sequences. West Nile virus encephalitis: While West Nile can cause encephalitis, the MRI findings can vary, but they do not typically present as bilateral occipital/parietal hyperintensities.

Step-by-step Reasoning: ☐ Correct ☐ Partially correct ☐ Incorrect

Reason/Comment

Based on the history provided, the patient has recently started taking cyclosporine. Cyclosporine is known to be associated with PRES. The MRI findings of bilateral occipital and parietal hyperintensities on T2/FLAIR sequences are characteristic of PRES. While the patient does have hypertension (a known risk factor for PRES), the other potential diagnoses either do not align with the MRI findings (like ADEM, multifocal ischemic infarcts, and West Nile virus encephalitis) or the patient's clinical history (like methanol ingestion).

Final Answer: ☐ True ☐ False

Answer: D. Posterior reversible encephalopathy syndrome (PRES).

Supplementary Fig. 3. Annotation interface for human physicians to provide their judgments on whether each section (image comprehension, recall of medical knowledge, step-by-step reasoning, and the final answer) of the GPT-4V response contains errors.

Supplementary Table 1. The physician accuracy, GPT-4V accuracy, and the identified hidden flaws for each specialty.

Specialty	Physician accuracy	GPT-4V accuracy	Hidden Flaws
Dermatology	79.4%	94.1%	18.8%
Pathology	70.6%	82.4%	42.9%
Pulmonary	85.7%	81.0%	52.9%
Gastrointestinal	75.9%	79.3%	39.1%
Neurology	84.6%	100.0%	53.8%
Ophthalmology	88.0%	80.0%	60.0%
Cardiology	61.5%	92.3%	0.0%
Infectious diseases	95.2%	66.7%	64.3%
Internal Medicine	61.8%	70.6%	8.3%
All	77.8%	81.6%	35.5%

Supplementary Table 2. Summary of relevant studies in comparison to our evaluation.

Evaluation	Multi-choice Accuracy		GPT-4V Rationale Evaluation
	GPT-4V	Human Performance	
92 cases from radiology and pathology websites[10]	NA	NA	Yes (all) & qualitative by ad-hoc comments
133 from medical visual question answering datasets[11]	29.9% (pathology) 50.0% (radiology)	NA	Partial (through case studies in the error analysis of GPT-4V)
226 USMLE examination questions[12]	84.2%-88.9%	NA	Partial (through case studies in the error analysis of GPT-4V)
934 NEJM Image Challenge[13]	61% (scored by GPT-4)	49% (NEJM online anonymous respondents)	NA
207 NEJM Image Challenge (this study)	81.6% (scored by physicians)	61.4% (medical student) 77.8% (physicians)	Yes (all 207 cases) & systematically on three distinct capabilities
