# Supplementary material for: Hidden Flaws Behind Expert-Level Accuracy of GPT-4 Vision in Medicine
Source: ArXiv. 2024 Jan 24:arXiv:2401.08396v2. Preprint. [Version 2] (PMC10896362)
Supplement: Supplementary file 1 [file NIHPP2401.08396v2-supplement-1.pdf]

Supplementary materials

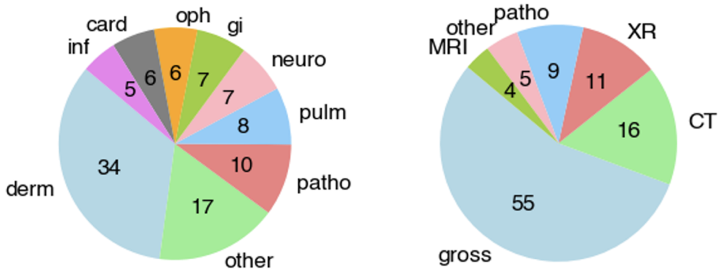

Supplementary Figure 1: The medical specialty and imaging modality distributions of the 100 NEJM Image Challenges used in this study. (derm: dermatology, other: other internal medicine, pulm: pulmonology, neuro: neurology, gi: gastroenterology, oph: ophthalmology, card: cardiology, inf: infectious diseases; gross: macroscopic clinical images, patho: microscopic pathology, CT: computed tomography, XR: X-ray, MRI: magnetic resonance imaging; other: other image types including electrocardiogram, echocardiogram, etc.).

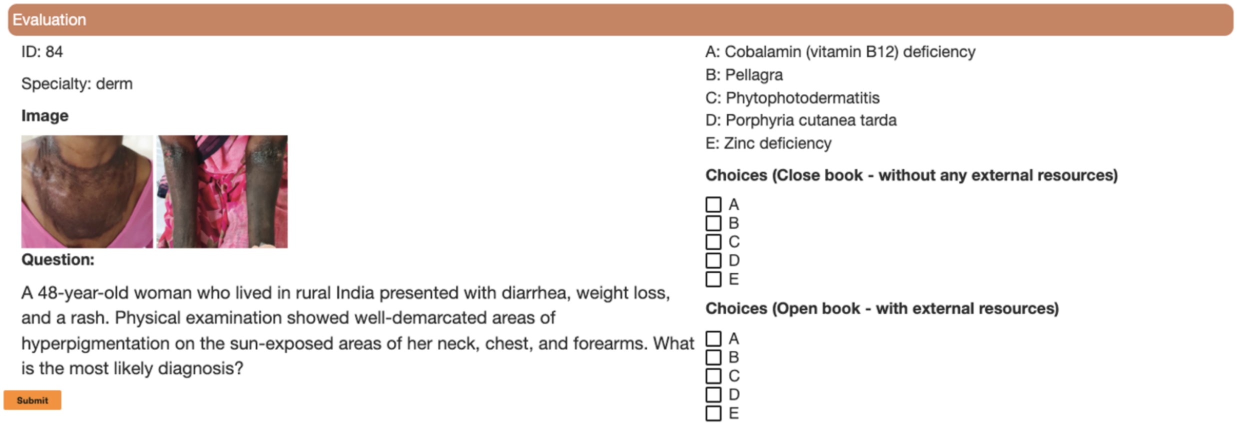

Supplementary Figure 2: Annotation interface for human physicians to provide their closed-book and open-book answers to NEJM Image Challenge.

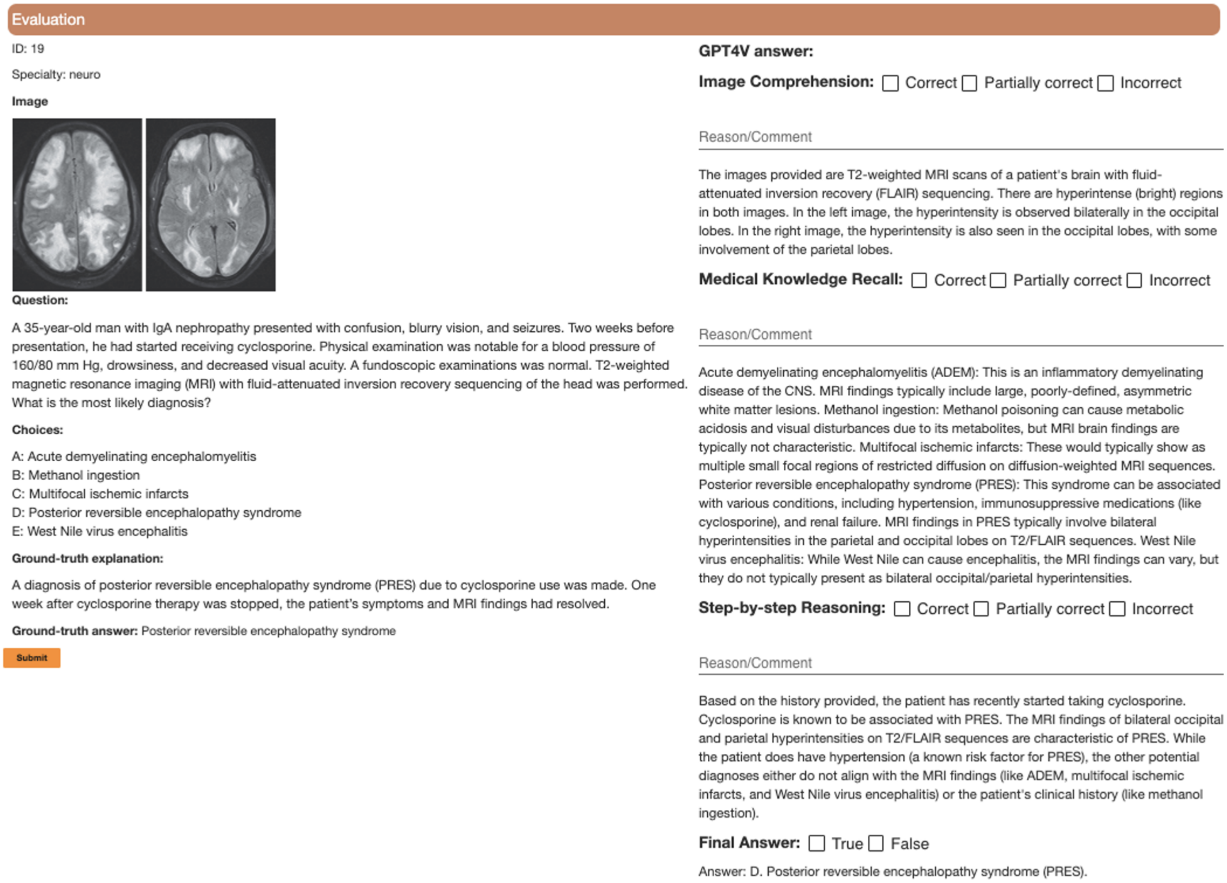

Supplementary Figure 3: Annotation interface for human physicians to provide their judgments on whether each section (image comprehension, recall of medical knowledge, step-by-step reasoning, and the final answer) of the GPT-4V response contains errors.

Supplementary Table 1: Summary of relevant studies in comparison to our evaluation

Evaluation	Multi-choice Accuracy		GPT-4V Rationale Evaluation
	GPT-4V	Human Performance	
92 cases from radiology and pathology websites [10]	NA	NA	Yes (all) & qualitative by ad-hoc comments
133 from medical visual question answering datasets [11]	29.9% (pathology), 50.0% (radiology)	NA	Partial (through case studies in the error analysis of GPT-4V)
226 USMLE examination questions [13]	84.2%-88.9%	NA	Partial (through case studies in the error analysis of GPT-4V)
934 NEJM Image Challenge [12]	61% (scored by GPT-4)	49% (NEJM online anonymous respondents)	NA
100 NEJM Image Challenge (this study)	88% (scored by physicians)	60% (medical student), 77% (physicians)	Yes (all 100 cases) & systematically on three distinct capabilities

Q1. NEJM Image Challenge 10/05/2023

A 72-year-old man presented to the emergency department with a 2-day history of an itchy rash on his back. On physical examination, edematous, flagellate plaques and linear patches were present across the patient's entire back and upper buttocks. There was no adenopathy, dermographism, or mucosal involvement. What substance had the patient most likely handled and ingested before subsequently developing this rash?

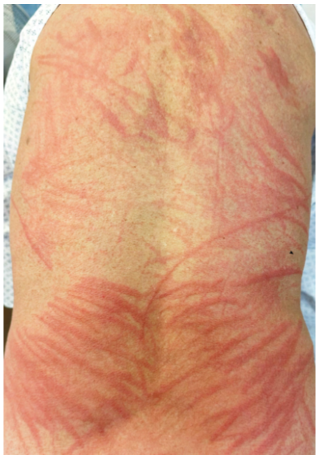

A. Phenytoin
B. Shellfish
C. Shiitake mushrooms
D. Toxicodendron radicans (poison ivy)
E. Warfarin

Correct Answer: Shiitake mushrooms
Explanation: The correct answer is shiitake mushrooms. Two days before the onset of symptoms, the patient shared that he had prepared and eaten a meal containing shiitake mushrooms. Shiitake dermatitis occurs after consumption of raw or undercooked shiitake mushrooms. The characteristic streaky rash — also known as flagellate erythema — may also be seen after exposure to bleomycin or, in rare cases, in dermatomyositis or adult-onset Still's disease. The condition is self-limited. The patient was treated with topical glucocorticoids and oral antihistamines for symptom management. At 2-week follow-up, residual areas of postinflammatory hyperpigmentation were present, but the itchiness had abated.

GPT-4V Answer

**Image Comprhension:** The image displays a close-up view of a person's skin, specifically the back area. The skin is marked with pronounced red, linear, and streaky welts or rashes. The pattern of these rashes appears like whip marks or as described, "flagellate."	**Annotation:** Correct<br>**Comment:** None
**Recall of Medical Knowledge:** Phenytoin is an antiepileptic drug. Its side effects can sometimes include a rash, but it doesn't typically manifest as a flagellate rash.<br>Shellfish allergy can cause a variety of symptoms, including skin reactions. However, it more commonly leads to urticaria (hives) or anaphylaxis rather than a flagellate pattern of rash.<br>Shiitake mushrooms can cause a dermatologic condition called Shiitake dermatitis. This condition is characterized by a flagellate rash on the body after consuming raw or undercooked shiitake mushrooms.<br>Toxicodendron radicans (poison ivy) induces contact dermatitis, which results in itchy, blistering rashes. The rash can be linear if a person brushed against the plant, but it doesn't typically produce a flagellate pattern.<br>Warfarin is an anticoagulant. One of its side effects can be skin necrosis, but this is quite distinct from a flagellate rash.	**Annotation:** Correct<br>**Comment:** None
**Step-by-step Reasoning:** Given the flagellate pattern of the rash and the absence of other symptoms such as adenopathy, dermographism, or mucosal involvement, Shiitake dermatitis becomes a primary consideration. Among the options provided:<br>Phenytoin, shellfish, and warfarin are not known to cause flagellate rashes. Poison ivy can cause linear rashes but they tend to be more associated with blistering and are not described as flagellate. Only Shiitake mushrooms are known to cause this specific type of flagellate rash following ingestion.	**Annotation:** Correct<br>**Comment:** None
**Final Answer:** Thus, based on the presentation and the distinctive appearance of the rash in the image, the patient had most likely handled and ingested C. Shiitake mushrooms before developing this rash.	**Annotation:** Correct

Q2. NEJM Image Challenge 9/28/23

A 55-year-old man presented with 10 years of progressive handwriting impairment and rapid, slurred speech. In his thirties, he had worked as a welder without access to personal protective equipment. Neurologic examination was notable for reduced facial expression, blepharospasm, and cluttered, dysarthric speech. Postural reflexes were mildly impaired. MRI imaging of the head showed a nonenhancing, T1-weighted, hyperintense signal in the basal ganglia on both sides. Ceruloplasmin and iron levels were normal. What treatment should be administered?

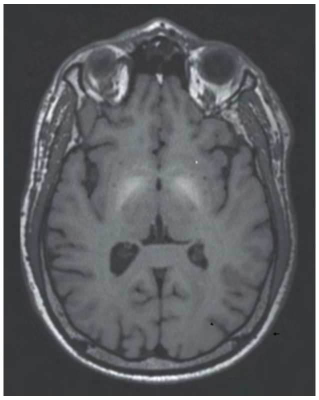

A. Carbidopa-levodopa
B. Chelation therapy
C. Cholinesterase inhibitors
D. Manganese repletion
E. Phlebotomy

Correct Answer: Chelation therapy
Explanation: The correct answer is chelation therapy. Based on the patient's occupational exposure history, parkinsonism, and blepharospasm, a diagnosis of manganese poisoning was made. Chelation treatment with intravenous EDTA was administered for 6 months, and the patient's symptoms and abnormal findings on MRI abated.

GPT-4V Answer

**Image Comprhension:** The provided image appears to be an MRI scan of a brain, specifically a T1-weighted axial section. There is bilateral hyperintensity, particularly in the basal ganglia region, which is the deep-seated group of nuclei in the brain responsible for various functions including movement coordination. The described hyperintense signal is evident in both hemispheres.	**Annotation:** Correct<br>**Comment:** None
**Recall of Medical Knowledge:** The basal ganglia, especially the globus pallidus, are the primary regions in the brain affected by manganese toxicity. Manganese toxicity, or manganism, can result from chronic exposure to high levels of manganese, such as might occur in occupational settings like welding, especially when personal protective equipment is not used. Clinical manifestations of manganism can mimic Parkinson's disease (tremors, bradykinesia, postural instability) but often also include psychiatric symptoms and dystonia. The mentioned symptoms like handwriting impairment, slurred speech, reduced facial expression, and blepharospasm are consistent with manganism. The primary treatment for manganese toxicity is removal from the source of exposure and chelation therapy.	**Annotation:** Correct<br>**Comment:** This is interesting. It is different from prior demonstrations of medical knowledge recall where it had listed information about all the choices. Instead, it is describing information about the findings on imaging combined with the information from the history as it relates to manganese toxicity.
**Step-by-step Reasoning:** The patient has a history of working as a welder without personal protective equipment, which puts him at risk for manganese exposure. The MRI shows hyperintensity in the basal ganglia, which is consistent with manganese deposition. His clinical symptoms, including handwriting impairment, slurred speech, and reduced facial expression, fit the clinical picture of manganism. Given that ceruloplasmin and iron levels were normal, conditions like Wilson's disease or hemochromatosis are less likely. Therefore, the most appropriate treatment for suspected manganese toxicity in this patient would be chelation therapy.	**Annotation:** Correct<br>**Comment:** None
**Final Answer:** Answer: B. Chelation therapy.	**Annotation:** Correct

Q3. NEJM Image Challenge 9/21/23

A 54-year-old woman with asthma and allergic rhinitis presented with a 3-month history of productive cough and dyspnea. She also reported fevers, chills, night sweats, and an unintentional 9-kg (20-lb) weight loss. On lung examination, there was expiratory wheezing and diffuse crackles. Laboratory testing showed pronounced hypereosinophilia. A computed tomography of the chest showed upper lobe–predominant peripheral and subpleural consolidations that spared the perihilar region (shown). Serum testing for IgE against Aspergillus fumigatus, antibodies against coccidioides, and antineutrophil cytoplasmic antibodies was negative. Bronchoscopy with bronchoalveolar lavage was notable for 74

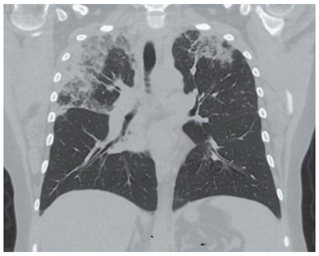

A. Allergic bronchopulmonary aspergillosis
B. Chronic eosinophilic pneumonia
C. Cryptogenic organizing pneumonia
D. Drug-Induced eosinophilic pneumonia
E. Pulmonary tuberculosis

Correct Answer: Chronic eosinophilic pneumonia
Explanation: A diagnosis of chronic eosinophilic pneumonia was made. Airspace consolidation with a predominantly peripheral distribution is commonly present in this condition, as well as peripheral eosinophilia, which is usually notably absent in acute eosinophilic pneumonia. Treatment with oral prednisone was started. At follow-up 17 days later, the patient's symptoms had abated and the radiographic findings had resolved. The patient continued to take prednisone for 5 months and had no further flare-ups.

GPT-4V Answer

**Image Comprhension:** The image appears to be a computed tomography (CT) scan of the chest, showing the lungs in a coronal view. There are notable areas of opacity, or consolidations, in the upper lobes of both lungs. These consolidations are predominantly located peripherally and subpleurally, and they seem to spare the perihilar region of the lungs. The pattern of distribution is suggestive of a certain lung disease or condition.	**Annotation:** Partially correct<br>**Comment:** This statement is vague: The pattern of distribution is suggestive of a certain lung disease or condition.
**Recall of Medical Knowledge:** Allergic bronchopulmonary aspergillosis (ABPA) usually occurs in patients with asthma or cystic fibrosis and is caused by a hypersensitivity reaction to Aspergillus species. CT findings in ABPA typically show bronchiectasis, mucus impaction, and high-attenuation mucus. Serum IgE against Aspergillus fumigatus is often elevated. Chronic eosinophilic pneumonia (CEP) is characterized by peripheral and subpleural lung opacities on imaging, and patients usually have eosinophilia on blood tests and bronchoalveolar lavage. Cryptogenic organizing pneumonia (COP), formerly known as bronchiolitis obliterans organizing pneumonia (BOOP), presents with patchy consolidations on imaging. Drug-Induced eosinophilic pneumonia results from a drug reaction and often presents with eosinophilia and pulmonary infiltrates on imaging. Pulmonary tuberculosis is caused by the bacterium Mycobacterium tuberculosis and can present with various radiographic patterns, including upper lobe consolidations, cavitation, and lymphadenopathy.	**Annotation:** Correct<br>**Comment:** None
**Step-by-step Reasoning:** Given that serum testing for IgE against Aspergillus fumigatus is negative and the CT findings do not predominantly show bronchiectasis or mucus impaction, ABPA (Option A) is less likely. The presentation with peripheral and subpleural consolidations, especially in the upper lobes that spare the perihilar region, along with pronounced hypereosinophilia in both blood and bronchoalveolar lavage, fits the profile of CEP (Option B). While COP (Option C) can have some similar radiographic findings, the pronounced eosinophilia in this case makes CEP more likely. There is no mention of drug exposure or new medications that could have led to drug-induced eosinophilic pneumonia (Option D). The presentation does not fit the typical findings for pulmonary tuberculosis (Option E), especially with negative tests for infectious diseases.	**Annotation:** Correct<br>**Comment:** None
**Final Answer:** Answer: Based on the given information and the image findings, the most likely diagnosis is B. Chronic eosinophilic pneumonia.	**Annotation:** Correct

Q4. NEJM Image Challenge 9/14/23

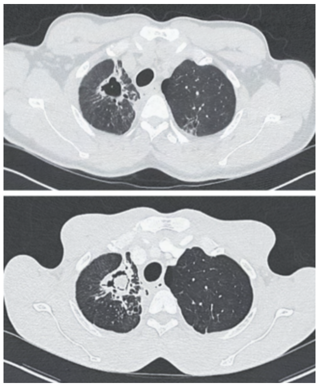

A 51-year-old man from the Democratic Republic of Congo presented to the emergency department with a 1-week history of small-volume hemoptysis. Sixteen months before presentation, computed tomography (CT) of the chest had shown a right upper lung cavitation that was a sequela of treated pulmonary tuberculosis (top). At the current presentation, a repeat CT of the chest showed thickening of the wall of the right upper lung cavitation and a new intracavitary mass (bottom). Which of the following agents may be used in the management of this condition?

A. Azithromycin
B. Rifampin
C. Rituximab
D. Trimethoprim-sulfamethoxazole
E. Voriconazole

Correct Answer: Voriconazole
Explanation: The patient was diagnosed with pulmonary aspergilloma after a serum sample was positive for aspergillus-specific IgG antibodies. Aspergillus fumigatus was identified by means of mass spectrometry. Concurrent tuberculosis was ruled out. Treatment with voriconazole was initiated. The patient was lost to follow-up before plans for a lobectomy could be made. Fourteen months later, he presented with massive hemoptysis and underwent pulmonary-artery embolization.

GPT-4V Answer	
**Image Comprhension:** The images provided are CT scans of the chest, with axial sections at the level of the lungs. The top image shows a cavitary lesion in the right upper lobe of the lung. This cavity is likely a sequela of treated pulmonary tuberculosis, as mentioned. The bottom image, taken 16 months later, reveals changes in the same cavitary lesion. The wall of the cavity appears to be thicker, and there is a new intracavitary mass present.	**Annotation:** Correct<br>**Comment:** None
**Recall of Medical Knowledge:** Cavitary lesions in the lungs can result from various etiologies, including tuberculosis, certain bacterial infections, fungal infections, and some malignancies. Since this patient's cavity was previously associated with tuberculosis and has now developed thickening and an intracavitary mass, one potential concern is a secondary infection or colonization of the cavity. Among the organisms known to colonize pre-existing lung cavities are the fungi from the genus Aspergillus, leading to a condition called aspergilloma or "fungus ball." Aspergilloma often presents with hemoptysis, matching the clinical scenario described. Voriconazole is an antifungal medication used to treat various Aspergillus infections.	**Annotation:** Correct<br>**Comment:** None
**Step-by-step Reasoning:** The patient presents with hemoptysis, and the CT findings show progression of a previously known cavitary lesion in the right upper lung. Given the clinical presentation and the imaging findings of an intracavitary mass in a previously known cavitary lesion, aspergilloma is a strong differential diagnosis. Aspergilloma, or fungus ball, is a non-invasive fungal infection resulting from colonization by Aspergillus species in a pre-existing lung cavity. Voriconazole is a first-line treatment option for Aspergillus infections. Therefore, among the options provided, voriconazole (E) is the most appropriate agent for managing this condition.	**Annotation:** Correct<br>**Comment:** None
**Final Answer:** Answer: E. Voriconazole.Answer: E. Voriconazole	**Annotation:** Correct

Q5. NEJM Image Challenge 9/7/23

A 34-year-old man presented to the emergency department with a 10-day history of shortness of breath that worsened when he bent forward or laid supine and abated when he sat upright. His respiratory rate was 36 breath per minute, and his oxygen saturation was 98

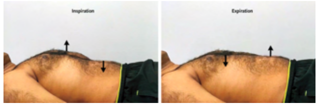

A. Chest radiography
B. Measurement of inspiratory pressures
C. Polysomnography
D. Pulmonary function tests
E. Ultrasonography of the diaphragm

Correct Answer: Polysomnography
Explanation: All of the above tests except for polysomnography could be used to identify bilateral diaphragmatic paralysis – the diagnosis that should be considered when a patient has paradoxical abdominal movements during respiration. In this case, chest radiographs obtained during inspiration and expiration showed a lack of diaphragmatic movement on both sides. Spirometry also identified a decrease in forced vital capacity of more than 50

GPT-4V Answer

**Image Comprhension:** The image shows a man in a supine position with two different states labeled "Inspiration" and "Expiration". In the "Inspiration" state, the abdominal wall appears to move inward, and in the "Expiration" state, the abdominal wall appears to move outward. The movement of the abdominal wall is paradoxical during the respiratory cycle when the patient is in a supine position.	**Annotation:** Correct<br>**Comment:** None
**Recall of Medical Knowledge:** Paradoxical movement of the abdominal wall, wherein it moves inward during inspiration and outward during expiration, is indicative of diaphragmatic dysfunction. The diaphragm is the primary muscle of inspiration. Its dysfunction can cause respiratory distress and a characteristic paradoxical movement. The tests to evaluate diaphragmatic dysfunction include: Chest radiography: To visualize diaphragmatic elevation or other abnormalities. Measurement of inspiratory pressures: To measure the strength of respiratory muscles. Pulmonary function tests: To assess overall lung function and detect restrictive patterns that can be caused by diaphragmatic dysfunction. Ultrasonography of the diaphragm: To directly visualize diaphragmatic movement and thickness. Polysomnography is a type of sleep study used to diagnose sleep disorders, including obstructive sleep apnea.	**Annotation:** Correct<br>**Comment:** None
**Step-by-step Reasoning:** Given that the patient has paradoxical movement of the abdominal wall, there is a strong indication of diaphragmatic dysfunction. Tests like chest radiography, measurement of inspiratory pressures, pulmonary function tests, and ultrasonography of the diaphragm are all relevant to diagnose and understand the extent of diaphragmatic dysfunction. Polysomnography, on the other hand, is not directly relevant to diaphragmatic dysfunction but rather to sleep disorders.	**Annotation:** Correct<br>**Comment:** None
**Final Answer:** Based on the above reasoning, the answer is: C.Polysomnography is not a test used to confirm the cause of the patient's dyspnea.	**Annotation:** Correct

Q6. NEJM Image Challenge 8/31/23

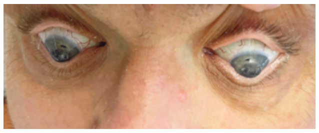

A 53-year-old man who had been admitted to the hospital after a fall was noted to have an abnormal indentation of the lower eyelids. He had a history of corneal transplantation in both eyes. He had recently experienced progressive visual impairment, which had led to his fall. Ophthalmologic examination was notable for a deflection of the lower eyelids when he was looking down, owing to dome-shaped eyes, and decreased visual acuity. What is the most likely diagnosis?

A. Astigmatism
B. Corneal ulcer
C. Keratoconus
D. Keratoglobus
E. Pellucid marginal degeneration

Correct Answer: Keratoconus
Explanation: The correct answer is keratoconus. Keratoconus is a noninflammatory disorder characterized by corneal thinning and bulging outward in a cone shape. Downward deflection of the eyelid on downward gaze, or Munson's Sign, may be seen in severe cases. Given the advanced nature of his disease, this patient was referred to ophthalmology for repeat corneal transplantation

GPT-4V Answer

**Image Comprhension:** The image displays a close-up view of a man's eyes. Both eyes exhibit an abnormal contour, with the cornea appearing to bulge outward in a dome shape. The lower eyelids show a noticeable indentation, especially pronounced when aligned with the protruding cornea. The eyes themselves appear clear, but the abnormal shape is evident.	**Annotation:** Correct<br>**Comment:** None
**Recall of Medical Knowledge:** The cornea is the clear front surface of the eye. Various conditions can affect its shape and clarity, leading to visual disturbances. Among the conditions mentioned: Astigmatism is a refractive error due to an uneven curvature of the cornea or lens, causing blurred or distorted vision. Corneal ulcer is an open sore on the cornea, usually associated with infection and inflammation. Keratoconus involves a progressive thinning and cone-shaped protrusion of the cornea. This leads to vision distortion. Keratoglobus is a rare non-inflammatory condition where the cornea thins and bulges forward in a globular shape. It involves more of the cornea than keratoconus and leads to a significant protrusion. Pellucid marginal degeneration is a thinning of the cornea at its inferior margin, leading to a forward bulging.	**Annotation:** Partially correct<br>**Comment:** The explanation is not that good at distinguishing between keratoconus, keratoglobus, and pellucid marginal degeneration. However, the key thing that was missing was mention of Munson's sign, which is clearly demonstrated in the image and associated with keratoconus.
**Step-by-step Reasoning:** The patient's history of corneal transplantation suggests a significant past corneal condition. The image shows a profound forward bulging of the entire cornea, causing the lower eyelids to indent, especially when looking down. The patient's progressive visual impairment aligns with conditions that alter the corneal shape. The most distinguishing feature in the image is the generalized, globular bulging of the corneas. This feature is most consistent with Keratoglobus. Among the options provided, the description best aligns with keratoglobus, a rare non-inflammatory condition causing the entire cornea to thin and bulge forward in a pronounced globular manner.	**Annotation:** Incorrect<br>**Comment:** GPT failed to recognize the image as demonstrating Munson's sign and a cone-shaped protrusion consistent with keratoconus, rather than globular, diffuse protrusion consistent with keratoglobus.
**Final Answer:** Answer: D. Keratoglobus	**Annotation:** Incorrect

Q7. NEJM Image Challenge 8/24/23

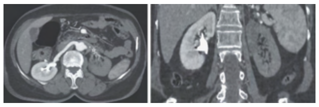

A 73-year-old woman presented with a 2-day history of left flank pain. Findings from a physical examination were normal. Laboratory testing was notable for a platelet count of 652,000 per cubic millimeter (reference range, 150,000 to 400,000) with an otherwise normal complete blood count. No previous platelet count was available. Computed tomography of the abdomen with intravenous contrast material showed a filling defect in the left renal artery and a perfusion defect in the left renal cortex, consistent with an occlusive thrombosis of the left renal artery and associated renal infarction. Further testing revealed no evidence of cardioembolic disease, renal artery injury, or inherited thrombophilia. Genetic testing was positive for a genetic variant. Which of the following variants is most likely to be associated with the above findings?

A. BCR-ABL1
B. CTLA4
C. FLT3
D. JAK2
E. MYC

Correct Answer: JAK2
Explanation: Owing to a persistently elevated platelet count, genetic testing was performed for the JAK2 V617F variant, and the result was positive. A diagnosis of essential thrombocythemia was made. Essential thrombocythemia may be complicated by arterial or venous thrombosis. The goal of treatment in patients with this condition is the prevention of vascular events. Treatment with hydroxyurea and aspirin was initiated. At follow-up 2 months later, the patient felt well and the platelet count was normal.

**GPT-4V Answer**	
**Image Comprhension:** The provided image displays a series of computed tomography (CT) scans of the abdomen. From left to right, the images depict:<br>An axial cross-section showing a prominent defect in the left renal artery. This defect is indicative of a filling defect or a possible clot or thrombus. Another axial image, this time focused more on the kidney itself, displaying an area of reduced contrast uptake in the left renal cortex. This is consistent with a perfusion defect, which usually indicates an area of tissue that is not receiving an adequate blood supply, possibly due to an obstruction or occlusion. A coronal cross-section revealing the length of the spine and the abdominal region. Again, there is an evident perfusion defect in the left kidney, corresponding to the region affected by the potential renal artery thrombosis.	**Annotation:** Correct<br>**Comment:** None
**Recall of Medical Knowledge:** Renal artery thrombosis can result in renal infarction if a segment or the entire kidney becomes ischemic due to reduced blood flow. Several genetic mutations or variants can be linked to a predisposition to clot formation or thrombosis. Among the options provided: BCR-ABL is associated with chronic myeloid leukemia (CML), which can present with increased platelet counts. CTLA variants are associated with immune regulation but not directly with clotting. FLT mutations can be found in hematologic malignancies but are not directly associated with thrombotic risk. JAK mutations can be associated with myeloproliferative disorders, which can manifest with elevated platelet counts and an increased risk of thrombosis. MYC is an oncogene but isn't directly associated with thrombotic events.	**Annotation:** Correct<br>**Comment:** None
**Step-by-step Reasoning:** The patient's presentation of an occlusive thrombosis of the left renal artery, associated renal infarction, and a notably high platelet count suggests a hypercoagulable state. The information doesn't provide any evidence of external factors that might have caused the thrombosis, such as injury or cardioembolic disease. In the context of the elevated platelet count and the absence of other identifiable causes for the thrombosis, we should consider genetic mutations that could be linked to conditions known for hypercoagulability. Among the given genetic variants, only BCR-ABL and JAK mutations are directly associated with conditions that could increase platelet counts and the risk of thrombosis. However, considering the clinical context of an elevated platelet count with no other obvious cause, a JAK mutation is more likely since it's associated with myeloproliferative disorders that can result in elevated platelet counts and thrombotic events.	**Annotation:** Correct<br>**Comment:** None
**Final Answer:** Answer: D.JAK.	**Annotation:** Correct

Q8. NEJM Image Challenge 8/17/23

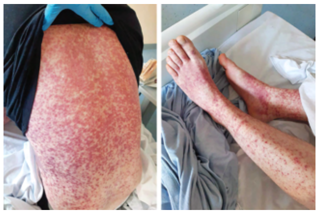

A 30-year-old male with recently diagnosed aplastic anemia presented to the emergency department with a 2-day history of fever, nonpruritic rash, and ankle and knee pain. Ten days prior, he had completed a course of horse anti-thymocyte globulin as a treatment for his aplastic anemia. Laboratory studies were notable for worsening neutropenia, low C3 and C4 levels, a CRP of 114 mg per liter (reference value < 5), and negative blood cultures. What type of hypersensitivity reaction is most likely responsible for this patient's clinical presentation?

A. Type I hypersensitivity reaction
B. Type II hypersensitivity reaction
C. Type III hypersensitivity reaction
D. Type IV hypersensitivity reaction
E. This is not a hypersensitivity reaction

Correct Answer: Type II hypersensitivity reaction
Explanation: This patient was diagnosed with serum sickness due to horse anti-thymocyte globulin. Serum sickness is a type III hypersensitivity reaction due to the formation of antigen-antibody or immune complexes. After a five-day course of intravenous glucocorticoids, the patient's symptoms resolved.

GPT-4V Answer

**Image Comprhension:** The image displays two parts, with the left side showing the back of a person with a widespread, reddish, non-blistering rash covering a large area of the back. The right side of the image shows both legs of a person, with a similar reddish rash distributed across the legs, particularly pronounced around the feet and ankles.	**Annotation:** Correct<br>**Comment:** None
**Recall of Medical Knowledge:** Hypersensitivity reactions are immune responses that are exaggerated or inappropriate against an antigen or allergen. There are four types of hypersensitivity reactions:<br>Type I hypersensitivity (Immediate hypersensitivity): This is an IgE-mediated response. Common examples include allergic asthma, anaphylaxis, and allergic rhinitis. Type II hypersensitivity (Cytotoxic hypersensitivity): This involves IgG or IgM antibodies directed against cellular or extracellular matrix antigens with complement fixation. Hemolytic anemia and some drug-induced hemolytic anemias are examples. Type III hypersensitivity (Immune complex-mediated hypersensitivity): This involves antigen-antibody complexes that deposit in tissues, activating the complement system and triggering inflammation. Common examples include serum sickness and some forms of glomerulonephritis. Type IV hypersensitivity (Delayed-type hypersensitivity): This is mediated by T cells, without involvement of antibodies. Tuberculin skin test and contact dermatitis are examples. Serum sickness is a systemic Type III hypersensitivity reaction that is characterized by fever, rash, arthritis, and sometimes glomerulonephritis. It can occur after exposure to certain drugs or other substances that act as antigens. The symptoms usually begin 1 to 2 weeks after exposure to the antigen.	**Annotation:** Correct<br>**Comment:** None
**Step-by-step Reasoning:** Given the patient's recent exposure to horse anti-thymocyte globulin, this is suggestive of serum sickness, which is a classic example of a Type III hypersensitivity reaction. The patient's clinical presentation aligns with typical features of serum sickness including fever, rash, joint pain, and laboratory abnormalities like low C3 and C4 levels (due to consumption by the immune complexes), and elevated CRP. Therefore, the most likely hypersensitivity reaction responsible for this patient's clinical presentation is a Type III hypersensitivity reaction.	**Annotation:** Correct<br>**Comment:** None
**Final Answer:** Answer: C. Type III hypersensitivity reaction.	**Annotation:** Correct

Q9. NEJM Image Challenge 8/10/23

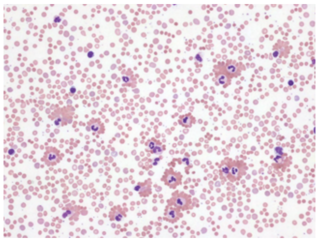

A 3-year-old girl presented to the emergency department with a 1-day history of dark urine and jaundice after the development of an upper respiratory tract infection 1 week earlier. The physical examination was notable for pharyngeal erythema and exudates, conjunctival pallor, and scleral icterus. There was no hepatosplenomegaly, rash, or acrocyanosis. Laboratory studies showed a new anemia and findings consistent with hemolysis. A direct antiglobulin test was positive for C3d and weakly positive for IgG. A subsequent peripheral blood smear is shown. What is the most likely diagnosis?

A. Cold agglutinin syndrome
B. G6PD Deficiency
C. Hemolytic uremic syndrome
D. Hereditary spherocytosis
E. Warm autoimmune hemolytic anemia

Correct Answer: Cold agglutinin syndrome
Explanation: The blood smear showed varied sizes and shapes of red cells that included reticulocytes and spherocytes, as well as erythrocyte agglutination. The cold agglutinin titer was 1:1024. A diagnosis of autoimmune hemolytic anemia from post-infectious cold agglutinin syndrome was made. Tests for Mycoplasma pneumoniae, Epstein–Barr virus, and other common respiratory viruses were negative. Supportive care, including blood transfusions and cold avoidance, was provided. At the 3-week follow-up, the child felt well and the anemia had abated.

GPT-4V Answer

**Image Comprhension:** The image is a peripheral blood smear. It displays numerous red blood cells of varying shapes, some of which are spherocytes (round cells without the central lightening that normal red cells have). There are also some cells that appear to be agglutinated, or clumped together. The absence of fragmented or "helmet-shaped" cells (schistocytes) is notable.<br>Recall of Medical Knowled	**Annotation:** Correct<br>**Comment:** None
**Recall of Medical Knowledge:** Cold agglutinin syndrome: This is caused by autoantibodies (usually IgM) that bind to red blood cells at cooler temperatures, leading to agglutination and hemolysis, especially in peripheral circulation (like fingers and toes). It can be triggered by infections, such as Mycoplasma pneumoniae or Epstein-Barr virus. The direct antiglobulin test (DAT) is often positive for C3d.<br>G6PD Deficiency: This is an X-linked recessive disorder where the red blood cells lack glucose-6-phosphate dehydrogenase enzyme, making them vulnerable to oxidative stress. Triggers include certain medications, fava beans, and infections. It doesn't typically cause agglutination on blood smear.<br>Hemolytic uremic syndrome (HUS): Often seen in children after a diarrheal illness caused by a specific strain of E. coli. It leads to hemolytic anemia, thrombocytopenia, and renal insufficiency. Peripheral smear usually shows schistocytes.<br>Hereditary spherocytosis: A genetic disorder causing a defect in red blood cell membrane proteins. It results in spherical red blood cells (spherocytes) that are less flexible and more prone to hemolysis. The direct antiglobulin test is negative in this condition.<br>Warm autoimmune hemolytic anemia (wAIHA): This is caused by autoantibodies, usually IgG, that bind to red blood cell surface antigens at body temperature, leading to hemolysis. The direct antiglobulin test is positive for IgG.	**Annotation:** Correct<br>**Comment:** None
**Step-by-step Reasoning:** The presence of spherocytes on the smear might suggest hereditary spherocytosis, but this is inconsistent with the positive direct antiglobulin test.<br>G6PD deficiency usually doesn't cause agglutination on blood smear.<br>The absence of schistocytes on the smear makes HUS an unlikely diagnosis.<br>The positive DAT for C3d with agglutination in the blood smear suggests that antibodies are binding to the red blood cells and causing them to clump, consistent with cold agglutinin syndrome. Additionally, the recent upper respiratory tract infection could serve as a trigger for this condition.	**Annotation:** Correct<br>**Comment:** None
**Final Answer:** Considering the information given, the image of the peripheral blood smear, and the recalled medical knowledge, the most likely diagnosis is: A. Cold agglutinin syndrome.	**Annotation:** Correct

Q10. NEJM Image Challenge 8/3/23

A 95-year-old woman presented with a 4-week history of dyspnea and dry cough. She had not previously reported these symptoms to her doctor. For the past 6 months, she had been taking nitrofurantoin daily to prevent recurrent urinary tract infections. Her oxygen saturation was 83

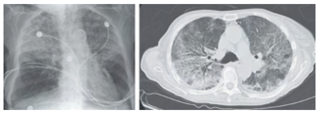

A. Antibiotics
B. Cessation of nitrofurantoin
C. Glucocorticoids
D. Intravenous loop diuretics
E. Scheduled nebulized bronchodilators

Correct Answer: Cessation of nitrofurantoin
Explanation: Owing to the patient's imaging findings and long-term nitrofurantoin exposure, a diagnosis of chronic nitrofurantoin-induced lung injury was made. Treatment with nitrofurantoin was stopped, and a prednisone taper was prescribed. Two months later, the patient's symptoms had abated and the bronchiectasis had stabilized.

GPT-4V Answer

**Image Comprhension:** The images presented consist of a chest radiograph (on the left) and computed tomography (CT) scans of the chest (on the right). The chest radiograph displays bilateral patchy opacities prominently in the upper lung fields. The CT scans of the chest similarly show bilateral diffuse ground-glass opacities in the upper lobes. There are no signs of consolidation, pleural effusion, or lymphadenopathy.	**Annotation:** Correct<br>**Comment:** None
**Recall of Medical Knowledge:** Nitrofurantoin is an antibiotic commonly used for the treatment and prevention of urinary tract infections. A known side effect of nitrofurantoin is pulmonary toxicity, which can manifest as acute or chronic pulmonary reactions. Acute reactions can occur within days to weeks of starting the drug and can present as fever, dyspnea, cough, and lung infiltrates on imaging. Chronic reactions can occur after long-term use (often months to years) and may present with symptoms similar to interstitial lung disease, including dyspnea, cough, and changes on chest imaging.	**Annotation:** Correct<br>**Comment:** None
**Step-by-step Reasoning:** The patient's presentation of dyspnea, dry cough, inspiratory crackles in the upper lung fields, and the imaging findings are consistent with a drug-induced lung injury. The fact that she has been on nitrofurantoin for the past 6 months and her symptoms started within this period strongly suggests nitrofurantoin-induced pulmonary toxicity. The primary treatment for drug-induced lung toxicity is to discontinue the offending drug. Antibiotics are not indicated unless there's evidence of bacterial infection, which in this case there isn't as the sputum culture was negative. Similarly, glucocorticoids are not first-line for drug-induced lung injury unless the condition doesn't improve or worsens after discontinuation of the drug. Loop diuretics would be indicated for pulmonary edema due to heart failure, which is not suggested here. Nebulized bronchodilators would be used for bronchospasm or COPD, which again isn't indicated by her symptoms or imaging findings.	**Annotation:** Correct<br>**Comment:** None
**Final Answer:** Answer: B. Cessation of nitrofurantoin.	**Annotation:** Correct

Q11. NEJM Image Challenge 7/27/23

A 13-year-old boy from Mali was referred to the pediatric urology clinic with a 3-month history of gross hematuria. He reported no fevers, flank pain, or dysuria. A physical examination was normal. Laboratory studies showed normal kidney function and an absolute eosinophil count of 2660 per cubic millimeter (reference range, 40-200). A urinalysis showed hematuria and pyuria, and a urine culture was negative. Microsopic examination of the urine is shown. What is the most likely diagnosis?

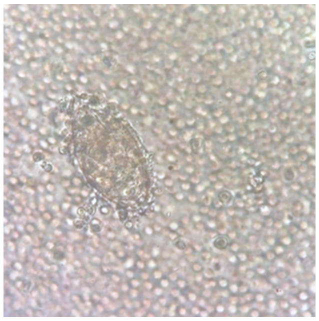

A. Balantidium coli
B. Schistosoma hematobium
C. Schistosoma mansoni
D. Strongyloides stercoralis
E. Trichomonas vaginalis

Correct Answer: Schistosoma hematobium
Explanation: The correct answer is Schistosoma hematobium, a parasite prevalent in Africa and the Middle East. The microscopic examination of urine showed oval-shaped parasite eggs with a terminal spine. Histopathological examination of a bladder-biopsy specimen showed acute and chronic papillary and polypoid cystitis, and no dysplasia. Treatment with praziquantel was administered, and symptoms resolved one month after initiation

GPT-4V Answer	
**Image Comprhension:** The image appears to be a microscopic view of a sample, possibly from the urine given the context provided. There is a prominent, elongated structure that looks like a helminthic parasite, surrounded by smaller, circular structures which could represent cells or other debris.	**Annotation:** Correct<br>**Comment:** None
**Recall of Medical Knowledge:** Balantidium coli is a protozoan parasite, which is the only ciliate known to infect humans. Its primary reservoir is pigs. It usually causes colitis. Schistosoma hematobium is a blood fluke that primarily affects the urinary system. Eggs of S. hematobium are typically passed in the urine, and the infection is often associated with hematuria. The adult flukes reside in the venous plexus of the bladder. Schistosoma mansoni is another species of blood fluke, but it primarily affects the liver and intestines. Eggs of S. mansoni are usually passed in the feces. Strongyloides stercoralis is an intestinal nematode, which can cause a hyperinfection syndrome, especially in immunocompromised individuals. Larvae, rather than eggs, are typically found in the stool. Trichomonas vaginalis is a flagellated protozoan parasite that primarily causes vaginal infections in women and urethral infections in men. It is typically detected by wet mount microscopy of vaginal or urethral secretions.	**Annotation:** Correct<br>**Comment:** None
**Step-by-step Reasoning:** Given the information:<br>The patient presents with gross hematuria. The urinalysis confirms hematuria and pyuria. There's an elevated eosinophil count, which is often associated with parasitic infections. The image suggests the presence of a helminthic (worm-like) parasite. From the provided choices:<br>Balantidium coli is unlikely given its protozoan nature and its primary association with gastrointestinal symptoms. Schistosoma mansoni affects the intestines and liver primarily. Strongyloides stercoralis is primarily an intestinal parasite. Trichomonas vaginalis affects the genitourinary tract but is protozoan and not typically associated with the observed presentation and findings. This narrows down our most likely culprit to Schistosoma hematobium, which is known to cause urinary symptoms, including hematuria. The observed helminthic structure in the image is consistent with the morphology of Schistosoma species, and the presentation and findings align well with an infection of S. hematobium.	**Annotation:** Correct<br>**Comment:** Great reasoning. For board exam another subtle clue is the spike at one pole of the egg.
**Final Answer:** Answer: B. Schistosoma hematobium.	**Annotation:** Correct

Q12. NEJM Image Challenge 7/20/23

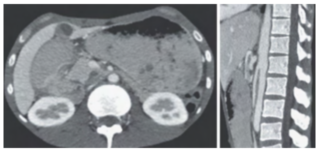

A 26-year-old man from Somalia presented with a 5-month history of dry cough, night sweats, and unintentional weight loss of 18 kg. During this period, epigastric pain and postprandial vomiting had also developed. His BMI was 11. On examination, he was cachectic with abdominal distention and diffuse tenderness to palpation. On the basis of chest imaging and sputum studies, a diagnosis of pulmonary tuberculosis was made, and intravenous antituberculous treatment was initiated. However, he continued to have postprandial vomiting. Contrast-enhanced CT of the abdomen was obtained. What is the cause of his abdominal symptoms?

A. Chronic mesenteric ischemia
B. Gastric tuberculosis
C. Gastrointestinal lymphoma
D. Intussusception
E. Superior mesenteric artery syndrome

Correct Answer: Superior mesenteric artery syndrome
Explanation: The correct answer is superior mesenteric artery syndrome. The CT scan of the abdomen showed a paucity of mesenteric and subcutaneous fat, dilatation of the stomach, and compression of the duodenum between the superior mesenteric artery and the aorta, with an aortomesenteric distance of 3 mm (reference range, 10 to 20) and an aortomesenteric angle of 7° (reference range, 45 to 60). A diagnosis of superior mesenteric artery syndrome due to cachexia from tuberculosis was made.

GPT-4V Answer

**Image Comprhension:** The provided image consists of three separate sections of a contrast-enhanced CT scan, primarily focusing on the abdomen. The scans reveal:<br>The first section (leftmost): A transverse view of the upper abdomen which shows a dilated stomach with retained content and significant distension. The surrounding vessels and structures appear normal.<br>The second section (middle): Another transverse view slightly below the first section, showing continued stomach dilation and distension. The duodenum appears to be compressed.<br>The third section (rightmost): A sagittal section of the abdomen, which provides a clearer view of the compressed duodenum between the aorta and the superior mesenteric artery.	**Annotation:** Partially correct<br>**Comment:** There are only 2 CT images, thus the GPT is misinterpreting this fact.
**Recall of Medical Knowledge:** Superior Mesenteric Artery (SMA) syndrome is a rare gastrointestinal condition where the third part of the duodenum is compressed between the aorta and the superior mesenteric artery. This compression results in partial or complete duodenal obstruction. The syndrome is often associated with conditions leading to rapid weight loss, which causes a loss of the mesenteric fat pad, thereby reducing the angle between the SMA and the aorta and leading to compression of the third part of the duodenum. Symptoms include postprandial vomiting, abdominal pain, and weight loss.	**Annotation:** Correct<br>**Comment:** None
**Step-by-step Reasoning:** The patient's history of rapid weight loss (18 kg) and the resultant low BMI of 11 supports the notion of a diminished mesenteric fat pad. The CT images show a dilated stomach with retained contents, which suggests a form of gastric outflow obstruction. The sagittal section of the abdomen distinctly reveals the third part of the duodenum being compressed between the aorta and the superior mesenteric artery. This anatomical change, combined with the patient's symptoms and clinical presentation, leads us to the diagnosis.	**Annotation:** Correct<br>**Comment:** None
**Final Answer:** Answer: E. Superior mesenteric artery syndrome.	**Annotation:** Correct

Q13. NEJM Image Challenge 7/13/23

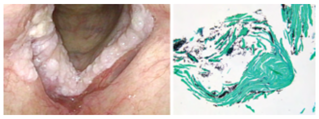

A 78-year-old man with chronic obstructive pulmonary disease (COPD) presented with a 2-month history of dysphonia. For the past 10 years, he had used an inhaled glucocorticoid daily to manage his COPD. Fiberoptic laryngoscopy revealed white plaques on both vocal cords. A biopsy showed hyperkeratinized stratified squamous epithelium and threadlike filaments that stained with Grocott-Gomori methenamine silver stain. What is the most likely diagnosis?

A. Laryngeal amyloidosis
B. Laryngeal candidiasis
C. Laryngeal papillomatosis
D. Leukoplakia
E. Vocal-cord dysfunction

Correct Answer: Laryngeal candidiasis
Explanation: The threadlike filaments that stained positive on Grocott-Gomori methenamine silver indicated a fungal presence. A diagnosis of laryngeal candidiasis was made. The patient received treatment with a course of fluconazole. At a 2-week follow-up visit, his voice had normalized and the laryngoscopic findings had improved.

GPT-4V Answer

**Image Comprhension:** The image displays two visual representations side by side.<br>On the left, there's a close-up view of what appears to be the vocal cords. The image shows whitish plaques coating the surfaces.<br>On the right, there's a microscopic image of tissue stained with Grocott-Gomori methenamine silver stain. The tissue sample displays hyperkeratinized stratified squamous epithelium, which is a type of skin tissue. There are also threadlike filaments scattered throughout the stained tissue.	**Annotation:** Correct<br>**Comment:** None
**Recall of Medical Knowledge:** Grocott-Gomori methenamine silver stain: This is a special fungal stain used in histopathology for fungi and some other organisms. Fungi will typically appear black on a light green background.<br>Laryngeal candidiasis: Also known as thrush, it's a fungal infection of the larynx, typically caused by Candida species. Chronic inhalation of corticosteroids, as seen in some COPD patients, is a risk factor. Candida infections can manifest as white plaques on mucosal surfaces, and it would stain positively with Grocott-Gomori methenamine silver stain.<br>Other options: Laryngeal amyloidosis involves the deposition of amyloid protein in the larynx, which wouldn't typically present with white plaques. Laryngeal papillomatosis is caused by HPV and results in wart-like growths. Leukoplakia leads to white patches on the mucous membranes, commonly due to irritants like smoking, but doesn't have fungal elements. Vocal-cord dysfunction is a functional disorder and wouldn't lead to the visual findings seen in the image.	**Annotation:** Correct<br>**Comment:** None
**Step-by-step Reasoning:** The presentation of white plaques on both vocal cords with the patient's history of inhaled corticosteroid use points towards a fungal infection, such as candidiasis. The Grocott-Gomori methenamine silver stain showing threadlike filaments further supports this diagnosis, as this is indicative of fungal elements. Given that laryngeal candidiasis presents as white plaques and would stain positively with the Grocott-Gomori methenamine silver stain, this is the most likely diagnosis.	**Annotation:** Correct<br>**Comment:** None
**Final Answer:** Answer: B. Laryngeal candidiasis.	**Annotation:** Correct

Q14. NEJM Image Challenge 7/6/23

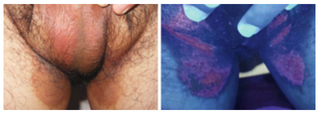

A 38-year-old man presented with a 9-month history of a mildly itchy rash in his groin. The rash had been previously diagnosed as tinea cruris, but it had not improved with topical antifungal treatment. On physical examination, well-circumscribed, reddish-brown plaques were visualized in the inguinal folds when the patient elevated his genitals (left image). No scaling or satellite lesions were present. A potassium hydroxide preparation of skin scrapings was negative. Under a Wood's lamp, the rash showed coral-red fluorescence (right image). What is the most likely causative organism?

A. Candida albicans
B. Corynebacterium minutissimum
C. Malassezia furfur
D. Pseudomonas aeruginosa
E. Trichophyton mentagrophytes

Correct Answer: Corynebacterium minutissimum
Explanation: Corynebacterium minutissimum grew on a bacterial culture of skin scrapings and a diagnosis of inguinal erythrasma was made. Erythrasma is a superficial bacterial skin infection caused by C. minutissimum. It typically manifests in the intertriginous or interdigital areas. It may be mistaken for a superficial fungal skin infection, but it can be differentiated from a dermatophytosis by its reddish-brown color and minimal scaling. A Wood's lamp examination facilitates diagnosis owing to the characteristic coral-red fluorescence, which is caused by porphyrins produced by the bacteria.

GPT-4V Answer	
**Image Comprhension:** The images provided display a close-up view of the groin region of a male patient. The left image shows a well-circumscribed, reddish-brown rash in the inguinal folds. There's no visible scaling or satellite lesions. The right image, taken under a Wood's lamp, reveals that the rash has a distinctive coral-red fluorescence.	**Annotation:** Correct<br>**Comment:** None
**Recall of Medical Knowledge:** Tinea cruris is a fungal infection of the groin region, often caused by the dermatophyte fungi like Trichophyton rubrum and Epidermophyton floccosum. Typically, it will have a positive potassium hydroxide (KOH) preparation showing fungal elements. Candida albicans is a common cause of skin and mucous membrane infections. Satellite lesions are often seen with candidal infections. Corynebacterium minutissimum causes erythrasma, a superficial skin infection. It produces coral-red fluorescence under Wood's lamp due to the production of porphyrins. Malassezia furfur causes pityriasis versicolor, a superficial fungal infection of the skin that may fluoresce yellow-gold under Wood's lamp. Pseudomonas aeruginosa is a bacterium that can cause a variety of infections, but a characteristic feature in skin infections is a green pigment. Trichophyton mentagrophytes is a dermatophyte fungus that can cause tinea infections in various parts of the body.	**Annotation:** Correct<br>**Comment:** None
**Step-by-step Reasoning:** Given the details:<br>The rash is reddish-brown, well-circumscribed in the inguinal folds. There is no scaling or satellite lesions. KOH preparation was negative, ruling out a fungal infection. The rash fluoresces a coral-red color under a Wood's lamp. Considering the recalled medical knowledge, the coral-red fluorescence under Wood's lamp is a characteristic feature of erythrasma caused by Corynebacterium minutissimum.	**Annotation:** Correct<br>**Comment:** None
**Final Answer:** Thus, the most likely causative organism for the rash presented in the images is: B. Corynebacterium minutissimum.	**Annotation:** Correct

Q15. NEJM Image Challenge 6/29/23

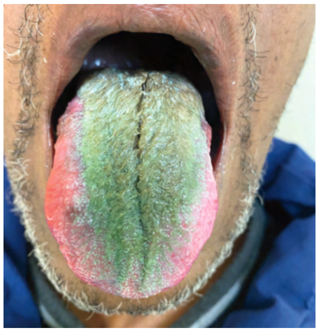

A 64-year-old man who reported current use of tobacco presented with a 2-week history of tongue discoloration. He reported no associated dysgeusia or tongue pain. Approximately 21 days before presentation, he had completed a course of clindamycin to treat a periodontal infection. After the tongue discoloration began, a course of fluconazole was prescribed to treat possible oral candidiasis. However, the tongue changes had persisted. Which of the following is the appropriate next step in management?

A. Apply topical budesonide
B. Counsel patient to stop smoking
C. Perform a biopsy
D. Prescribe nystatin
E. Screen for high risk sexual behaviors

Correct Answer: Counsel patient to stop smoking
Explanation: The patient was advised to gently scrub the surface of his tongue with a toothbrush four times daily and was counseled on smoking cessation. Hairy tongue is a benign condition that involves elongation and discoloration of the filiform papillae on the dorsal aspect of the tongue. Risk factors include smoking, dehydration, poor oral hygiene, and antimicrobial use.

GPT-4V Answer	
**Image Comprhension:** The image depicts a close-up view of an open mouth, revealing a tongue with notable discoloration. The tongue has a greenish hue mixed with some normal reddish-pink areas. The texture appears to be somewhat rough and patchy. Surrounding the tongue, the inner mouth region also displays some discoloration, but it's less pronounced.	**Annotation:** Correct<br>**Comment:** None
**Recall of Medical Knowledge:** Tongue discoloration can be caused by a variety of factors, including medications, infections, poor oral hygiene, and lifestyle habits like smoking. Clindamycin is an antibiotic that can alter the normal flora of the mouth, leading to fungal overgrowth such as oral candidiasis (thrush). Fluconazole is an antifungal medication commonly prescribed for oral candidiasis. A green or black discoloration of the tongue, known as "black hairy tongue" (lingua villosa), can result from the elongation of filiform papillae. Factors that can cause or contribute to this condition include poor oral hygiene, smoking, certain medications (including antibiotics like clindamycin), and a lack of regular tongue cleaning. Budesonide is a corticosteroid that can reduce inflammation but is not typically the first line of treatment for tongue discoloration unless there is an inflammatory cause. Nystatin is another antifungal that can be prescribed for oral candidiasis. Biopsy is a procedure used to take a sample of the affected tissue for diagnostic purposes.	**Annotation:** Correct<br>**Comment:** None
**Step-by-step Reasoning:** The patient has a history of clindamycin use, which can alter oral flora and lead to fungal overgrowth. However, the presentation is not consistent with typical oral candidiasis, and the patient's condition did not improve with fluconazole, an antifungal treatment. The greenish discoloration is more in line with black hairy tongue, a condition that can be exacerbated by smoking and certain medications. Before resorting to more invasive or intensive treatments, it would be prudent to address modifiable risk factors. Among the options provided, counseling the patient to stop smoking (option B) is the most appropriate next step. Smoking cessation can help in the resolution of the condition.	**Annotation:** Correct<br>**Comment:** None
**Final Answer:** Based on the above reasoning, the appropriate next step in management would be B. Counsel patient to stop smoking.	**Annotation:** Correct

Q16. NEJM Image Challenge 6/22/23

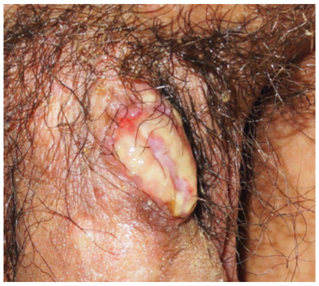

A 39-year-old man with human immunodeficiency virus infection that was being treated with antiretroviral therapy presented to the dermatology clinic with a 1-year history of recurrent, painful penile ulcers. Approximately once per month, erosions would appear ulcerate, heal spontaneously and then recur. Laboratory testing showed a CD4 cell count of 494 per cubic millimeter (reference range, 414 to 1123) and an HIV viral load of 450 copies per milliliter (reference range, <20). Biopsies of the lesions revealed epidermal necrosis, pseudoepitheliomatous epidermal hyperplasia, and a dense infiltrate of inflammatory cells in the dermis and subcutaneous tissue. Next-generation sequencing (NGS) of the tissue was performed. What is the most likely diagnosis?

A. Chancroid
B. Condyloma acuminata
C. Condyloma lata
D. Herpes vegetans
E. Pemphigus vegetans

Correct Answer: Herpes vegetans
Explanation: The correct answer is Herpes vegetans. Results from NGS detected herpes simplex virus (HSV) type 2. Herpes vegetans — also known as hypertrophic herpes simplex — is an uncommon manifestation of HSV infection seen in persons with immunocompromise. It manifests as verrucous, proliferative, or ulcerative lesions, often with a chronic, persistent course. Treatment with oral valacyclovir was given and at a 2-week follow-up, the lesions had abated.

GPT-4V Answer

**Image Comprhension:** The image depicts a close-up view of what appears to be a penile ulceration. The affected area has significant erythema (redness) surrounding the ulcer. The lesion itself seems deep and moist, with irregular borders. There are areas that look like eroded or raw skin with some crusting. The surrounding skin has hair, and the lesion is localized.	**Annotation:** Correct<br>**Comment:** None
**Recall of Medical Knowledge:** Chancroid is a sexually transmitted infection caused by the bacterium Haemophilus ducreyi. It results in painful genital ulcers and swollen lymph nodes in the groin. Condyloma acuminata are genital warts caused by human papillomavirus (HPV). They usually present as cauliflower-like growths on the genital or anal area. Condyloma lata are broad, moist, warty lesions found in the second stage of syphilis. Herpes vegetans is a rare variant of anogenital herpes infection in patients with HIV. It presents as exophytic, verrucous, or cauliflower-like growths. Pemphigus vegetans is a variant of pemphigus vulgaris. It presents with pustules that evolve into vegetative (wart-like) plaques typically in the folds of the skin, such as the groin or under the arms. It's an autoimmune blistering disorder.	**Annotation:** Correct<br>**Comment:** None
**Step-by-step Reasoning:** Given the provided clinical information and the appearance of the lesion in the image:<br>The lesion does not have the classic appearance of a chancroid, which would be a painful, soft, and necrotic genital ulcer. The lesion does not resemble the typical cauliflower-like appearance of condyloma acuminata. The lesion is not the broad, moist appearance of condyloma lata. Herpes vegetans seems like a plausible diagnosis given the appearance of the lesion, the HIV status of the patient, and the recurring nature of the ulcers. The exophytic, verrucous, or cauliflower-like growths associated with this diagnosis match the presentation. The lesion does not appear in a skin fold which is typical for pemphigus vegetans.	**Annotation:** Correct<br>**Comment:** None
**Final Answer:** Considering all the above, the most likely diagnosis based on the provided information and image comprehension is: D. Herpes vegetans.	**Annotation:** Correct

Q17. NEJM Image Challenge 6/15/23

A 67-year-old man with chronic lymphocytic leukemia (CLL) presented with a painful rash. On physical examination, there was purple discoloration of the ear along with livedoid skin changes on the cheek and purpura on both calves. A skin biopsy showed leukocytoclastic vasculitis. Laboratory testing was notable for a low complement 4 level. What is the most likely diagnosis?

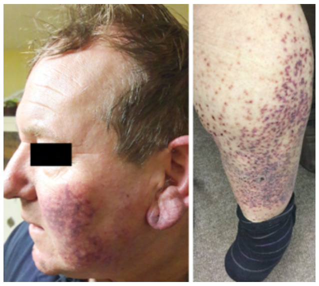

A. Behçet's disease
B. Cryoglobulinemic vasculitis
C. Granulomatosis with polyangiitis
D. Leukemia cutis
E. Microscopic polyangiitis

Correct Answer: Cryoglobulinemic vasculitis
Explanation: The patient reported the rash appeared when he was exposed to temperatures of 3℃ or less. Laboratory testing was also notable for a cryocrit level of 4

GPT-4V Answer

**Image Comprhension:** The image consists of two parts. On the left, there's a close-up of the side profile of a man's face. His eye area is covered with a black rectangle for privacy. He exhibits a purple discoloration of the ear and livedoid skin changes on the cheek, which appear as irregular, purple-red patches. On the right, there's an image of a lower leg showing purpura, which manifests as dark reddish-purple spots and patches on the skin.	**Annotation:** Partially correct<br>**Comment:** Would describe lower leg changes as petechiae
**Recall of Medical Knowledge:** Chronic lymphocytic leukemia (CLL): A type of cancer in which the bone marrow makes too many lymphocytes. CLL can lead to various skin manifestations. Leukocytoclastic vasculitis (LCV): An inflammation of the small vessels that results in the death of blood vessel cells. LCV can be associated with a range of systemic disorders and can present with purpura. Behçet's disease: A rare disorder causing inflammation in blood vessels. Its symptoms might include mouth sores, eye inflammation, and skin problems. Cryoglobulinemic vasculitis: A type of vasculitis associated with the presence of cryoglobulins in the serum. It affects small and medium-sized vessels and can be associated with purpura, low complement levels, and is often seen in the context of other diseases like hepatitis C or autoimmune diseases. Granulomatosis with polyangiitis: A condition that causes inflammation of the blood vessels in the nose, sinuses, throat, lungs, and kidneys. Leukemia cutis: Direct infiltration of the skin by malignant leukocytes, resulting in localized or generalized skin lesions. It's often associated with CLL. Microscopic polyangiitis: A systemic vasculitis affecting small vessels, associated with ANCA antibodies.	**Annotation:** Correct<br>**Comment:** None
**Step-by-step Reasoning:** Given the patient's background of CLL, the appearance of the skin changes in the image, and the biopsy results of leukocytoclastic vasculitis, we can reason through the options:<br>A. Behçet's disease: This is less likely as the primary features usually include oral ulcers, genital ulcers, and eye problems, which aren't mentioned here.<br>B. Cryoglobulinemic vasculitis: This is a strong possibility. It's associated with purpura and low complement levels (as seen in the patient). Additionally, LCV is a common histopathologic finding.<br>C. Granulomatosis with polyangiitis: While it's a vasculitis, it usually affects the upper respiratory tract and kidneys primarily.<br>D. Leukemia cutis: While CLL can manifest with skin changes due to direct infiltration, the biopsy showed LCV, which isn't consistent with leukemia cutis.<br>E. Microscopic polyangiitis: While it affects small vessels, it isn't specifically linked with CLL or low complement levels.	**Annotation:** Correct<br>**Comment:** None
**Final Answer:** Based on the presented symptoms, physical findings, and biopsy results, the most likely diagnosis is:<br>B. Cryoglobulinemic vasculitis.	**Annotation:** Correct

Q18. NEJM Image Challenge 6/8/23

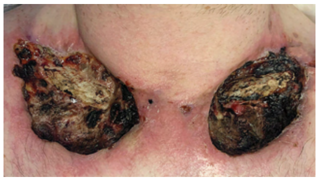

A 32-year-old man presented with 1-month history of enlarging necrotic chest wounds. He reported a 3-year history of daily use of fentanyl by injection into his neck and arm veins. CT of the chest showed osteomyelitis of the clavicles and manubrium, in addition to soft-tissue ulceration and inflammation. Adulteration of fentanyl by which of the following substances is most likely to have contributed to the development of these superinfected wounds?

A. Cocaine
B. Levamisole
C. Methamphetamine
D. Talcum powder
E. Xylazine

Correct Answer: Xylazine
Explanation: The correct answer is xylazine. Xylazine, also known as "tranq", is an 2-adrenergic receptor agonist that has been approved only for veterinary use. It is an increasingly prevalent additive to fentanyl in the United States. Xylazine is associated with skin injury, regardless of the site of injection. The patient underwent chest-wall debridement and microsurgical flap reconstruction, followed by a 6-week course of intravenous antimicrobial therapy. He also received buprenorphine therapy for his opioid-use disorder.

GPT-4V Answer

**Image Comprhension:** The image displays a close-up of a person's neck and upper chest region, which reveals two significant necrotic wounds. These wounds are blackened, with areas of exposed underlying tissue and some regions that appear crusty or scabbed over. The surrounding skin seems to be inflamed, reddened, and somewhat swollen. There are also some puncture marks visible on the skin, which might be associated with injection sites.	**Annotation:** Correct<br>**Comment:** None
**Recall of Medical Knowledge:** Fentanyl: An opioid analgesic primarily used for pain management but has also been associated with illicit drug use. Adulterants: Illicit drugs are frequently cut or adulterated with various substances to increase volume, potency, or to reduce costs. Some of these adulterants can have severe side effects. Cocaine: A stimulant drug, when used as an adulterant, is typically not associated with causing necrotic wounds. Levamisole: Previously used as an immunomodulatory and antihelminthic drug, but it is now more commonly recognized as an adulterant in cocaine. It has been associated with vasculitis, skin necrosis, and neutropenia. Methamphetamine: A potent stimulant drug. Its use can result in a variety of cutaneous manifestations, but large necrotic wounds like those in the image are not typical. Talcum powder: Sometimes used as a filler in illicit drugs, but it can cause granulomatous reactions when injected. It is not commonly associated with large necrotic skin wounds. Xylazine: An animal tranquilizer that has been identified as an adulterant in heroin and other illicit drugs. Xylazine can cause skin and soft tissue necrosis at the injection site.	**Annotation:** Correct<br>**Comment:** None
**Step-by-step Reasoning:** The presented image is indicative of severe necrotic wounds. Given the history of fentanyl injection and the described CT findings, the wounds are likely related to the fentanyl use. The adulterant causing these wounds would be one that is known to result in significant skin and soft tissue damage when injected. Among the choices provided, Cocaine usually doesn't cause the type of severe necrotic wounds seen in the image. Methamphetamine can cause various skin manifestations but not typically extensive necrosis like this. Talcum powder, when injected, might lead to granulomatous reactions but is not typically associated with such extensive necrotic wounds. Levamisole can indeed cause skin necrosis, but it's more commonly associated with smaller areas of necrosis and not the extensive damage observed here. Xylazine has been noted in the literature to cause significant skin and soft tissue necrosis, especially when used as an adulterant in injected drugs. This aligns most closely with the image's findings and the provided clinical history.	**Annotation:** Correct<br>**Comment:** None
**Final Answer:** Based on the above reasoning, the most likely adulterant in the fentanyl that contributed to the development of these superinfected wounds is E. Xylazine.	**Annotation:** Correct

Q19. NEJM Image Challenge 6/1/23

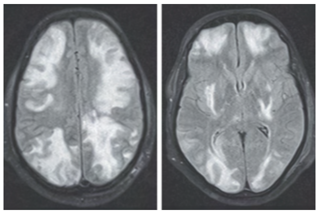

A 35-year-old man with IgA nephropathy presented with confusion, blurry vision, and seizures. Two weeks before presentation, he had started receiving cyclosporine. Physical examination was notable for a blood pressure of 160/80 mm Hg, drowsiness, and decreased visual acuity. A fundoscopic examinations was normal. T2-weighted magnetic resonance imaging (MRI) with fluid-attenuated inversion recovery sequencing of the head was performed. What is the most likely diagnosis?

A. Acute demyelinating encephalomyelitis
B. Methanol ingestion
C. Multifocal ischemic infarcts
D. Posterior reversible encephalopathy syndrome
E. West Nile virus encephalitis

Correct Answer: Posterior reversible encephalopathy syndrome
Explanation: A diagnosis of posterior reversible encephalopathy syndrome (PRES) due to cyclosporine use was made. One week after cyclosporine therapy was stopped, the patient's symptoms and MRI findings had resolved.

**GPT-4V Answer**	
**Image Comprhension:** The images provided are T2-weighted MRI scans of a patient's brain with fluid-attenuated inversion recovery (FLAIR) sequencing. There are hyperintense (bright) regions in both images. In the left image, the hyperintensity is observed bilaterally in the occipital lobes. In the right image, the hyperintensity is also seen in the occipital lobes, with some involvement of the parietal lobes.	**Annotation:** Partially correct<br>**Comment:** The descriptions of the findings were accurate, even describing the sequences. It correctly described the abnormal regions as being hyperintense (bright). The location of the hyperintensities correctly in the occipital lobes but did not mention hyperintensities seen in the biateral frontal lobes and parietal lobes on the L. It correctly identified locations on the right image as seen in the bilateral occipital lobes and parietal lobes but again missed bilateral frontal lobe involvement.
**Recall of Medical Knowledge:** Acute demyelinating encephalomyelitis (ADEM): This is an inflammatory demyelinating disease of the CNS. MRI findings typically include large, poorly-defined, asymmetric white matter lesions. Methanol ingestion: Methanol poisoning can cause metabolic acidosis and visual disturbances due to its metabolites, but MRI brain findings are typically not characteristic. Multifocal ischemic infarcts: These would typically show as multiple small focal regions of restricted diffusion on diffusion-weighted MRI sequences. Posterior reversible encephalopathy syndrome (PRES): This syndrome can be associated with various conditions, including hypertension, immunosuppressive medications (like cyclosporine), and renal failure. MRI findings in PRES typically involve bilateral hyperintensities in the parietal and occipital lobes on T2/FLAIR sequences. West Nile virus encephalitis: While West Nile can cause encephalitis, the MRI findings can vary, but they do not typically present as bilateral occipital/parietal hyperintensities.	**Annotation:** Partially correct<br>**Comment:** All are corret although there are specific findins with methanol ingestion: characteristic putaminal necrosis in the acute setting. Especially in the setting of this imaging, it is important to note (which GPT did not) that PRES does not have to be restrictred to occipital and parietal lobes.
**Step-by-step Reasoning:** Based on the history provided, the patient has recently started taking cyclosporine. Cyclosporine is known to be associated with PRES. The MRI findings of bilateral occipital and parietal hyperintensities on T2/FLAIR sequences are characteristic of PRES. While the patient does have hypertension (a known risk factor for PRES), the other potential diagnoses either do not align with the MRI findings (like ADEM, multifocal ischemic infarcts, and West Nile virus encephalitis) or the patient's clinical history (like methanol ingestion).	**Annotation:** Partially correct<br>**Comment:** Again, the bilateral frontal involvement is not mentioned which is still characteristic of PRES. But everything else is correct.
**Final Answer:** Answer: D. Posterior reversible encephalopathy syndrome (PRES).	**Annotation:** Correct

Q20. NEJM Image Challenge 5/25/23

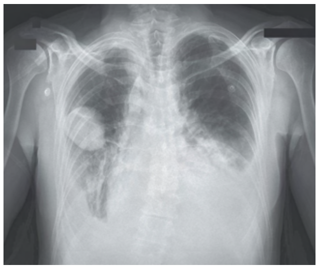

A 50-year-old man with a history of mitral-valve prolapse and of Hodgkin's lymphoma 20 years earlier presented to the emergency department with a 5-day history of dyspnea. Physical examination showed jugular venous distention, a holosystolic murmur at the cardiac apex, and diminished breath sounds in the lung bases. A radiograph of the chest was performed. What is the best next step in regard to the right lung opacity?

A. Collect a sputum culture and then initiate empiric antibiotics
B. Diurese the patient and then obtain a repeat chest radiograph
C. Obtain a computed tomography scan of chest
D. Perform a bronchoscopy
E. Perform a thoracentesis

Correct Answer: Diurese the patient and then obtain a repeat chest radiograph
Explanation: The correct answer is diurese the patient and then obtain a repeat chest radiograph. The chest radiograph showed signs of volume overload as well as a sharply demarcated, lenticular opacity in the right middle lung field. An echocardiogram showed an ejection fraction of 45

GPT-4V Answer

**Image Comprhension:** The image appears to be a chest radiograph (X-ray). The most striking feature on this image is an extensive, unilateral right-sided opacity that appears to occupy the entire lower and mid zone of the right lung. The opacity is homogenous with a clear demarcation between the affected and unaffected lung. The heart and other mediastinal structures appear to be shifted away from the opacity, suggesting a mass effect.	**Annotation:** Partially correct<br>**Comment:** There is also a large left pleural effusion which was not mentioned. I'm not sure the heart is shifted. There is also a scoliosis of the spine.
**Recall of Medical Knowledge:** A unilateral, large opacity on a chest radiograph could represent several pathologies: pneumonia (infection), pleural effusion (fluid accumulation in the pleural space), tumor, or atelectasis (lung collapse). Given the patient's history of mitral valve prolapse, there's an increased risk of developing infective endocarditis. One of the complications of infective endocarditis is septic emboli, which could cause pneumonia. Hodgkin's lymphoma can increase the risk of developing secondary malignancies, but also, the disease itself or its treatment could result in lung complications. Jugular venous distention and a holosystolic murmur might suggest congestive heart failure (CHF) which could cause pulmonary edema, but this would usually be bilateral and not restricted to one side. Physical examination findings of diminished breath sounds over the lung bases are consistent with pleural effusion or atelectasis but less likely with pneumonia.	**Annotation:** Partially correct<br>**Comment:** This statement is incorrect: but this would usually be bilateral and not restricted to one side. There are bilateral pleural effusions (a consequence of edema bilaterally).
**Step-by-step Reasoning:** Option A: Pneumonia is a possibility given the patient's history of mitral valve prolapse and risk of septic emboli. However, the unilateral, homogenous opacity makes this less likely. Option B: Diuresis might be indicated if the opacity was due to pulmonary edema from CHF, but this is typically bilateral, and the presentation doesn't fit perfectly with CHF. Option C: A CT scan would provide detailed imaging and might be helpful, especially if there's suspicion of a mass. Option D: Bronchoscopy might be useful if there was a concern for an obstructing lesion or to obtain samples, but this is not the first line of investigation given the current findings. Option E: The presentation with diminished breath sounds and unilateral opacity most fits with a pleural effusion. The best next step to both confirm the diagnosis and potentially provide therapeutic relief is thoracentesis, where fluid is aspirated from the pleural space.	**Annotation:** Partially correct<br>**Comment:** The oval opacity in the right lung is suspicious for a phantom tumor (fluid in the minor fissure). ChatGPT missed that entirely.
**Final Answer:** Answer: E. Perform a thoracentesis.	**Annotation:** Incorrect

Q21. NEJM Image Challenge 5/18/23

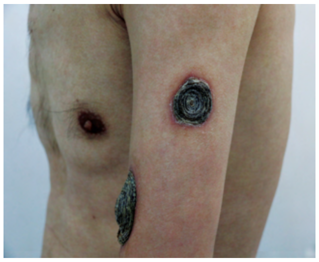

A 44-year-old man with a history of human immunodeficiency virus infection and intermittent adherence to antiretroviral therapy presented to the infectious disease clinic with a 1-month history of fevers and pruritic skin lesions. Physical examination was notable for blackish-brown lamellated plaques on the limbs and scalp. Laboratory studies were notable for a CD4 cell count of 86 per cubic millimeter (reference range, 414 to 1123) and a rapid plasmin reagin titer of 1:32. A skin biopsy of the left forearm showed diffuse dermal lymphocytes and histiocytes admixed with a plasma-cell infiltrate. What is the most likely diagnosis?

A. Erythema annulare centrifugum
B. Erythema gyratum repens
C. Erythema migrans (Lyme disease)
D. Lupus vulgaris (cutaneous tuberculosis)
E. Malignant syphilis

Correct Answer: Malignant syphilis
Explanation: The correct answer is malignant syphilis. A Treponema pallidum particle agglutination assay was also positive. Malignant syphilis is an uncommon form of secondary syphilis that primarily affects immunocompromised persons. The skin manifestations are varied, ranging from ulceronodular lesions to oyster shell-like crusts, as seen in this patient. After a 2-week course of intravenous antimicrobial therapy, the skin lesions and fevers abated.

GPT-4V Answer	
**Image Comprhension:** The image displays a close-up of skin, revealing two main features: A raised blackish-brown plaque with a lamellated appearance. This lesion appears to have concentric rings, giving it a layered look. There is also a darker, brownish circular mark on the skin, which may or may not be related to the patient's presenting symptoms.	**Annotation:** Incorrect<br>**Comment:** The more distal plaque likely represents the same finding as the proximal lesion.
**Recall of Medical Knowledge:** Erythema annulare centrifugum: Presents as annular (ring-shaped) red plaques that tend to be itchy. Erythema gyratum repens: Characterized by concentric rings of erythema that give a wood-grain appearance. It can be associated with internal malignancies. Erythema migrans (Lyme disease): Presents as a red, expanding rash with a clear center, resembling a "bull's-eye." Lupus vulgaris (cutaneous tuberculosis): Chronic and progressive form of skin TB, with reddish-brown nodules and plaques. Malignant syphilis: Also known as lues maligna, is a rare form of secondary syphilis. It manifests as papules, nodules, or ulcers, often covered by a grayish-white membrane or necrotic tissue. Given the patient's HIV status and the described lab findings, this condition becomes more pertinent.	**Annotation:** Correct<br>**Comment:** None
**Step-by-step Reasoning:** Given the description and the image, the skin lesion does not resemble Erythema annulare centrifugum, Erythema gyratum repens, or Erythema migrans. While Lupus vulgaris could present with nodules and plaques, the blackish-brown lamellated appearance of the lesion is not typical for cutaneous tuberculosis. Malignant syphilis, on the other hand, can have varied manifestations, including the described lesion appearance. Moreover, the patient's HIV infection and low CD4 cell count make him more susceptible to atypical infections, including unusual presentations of syphilis. The rapid plasmin reagin (RPR) titer of 1:32 further supports a syphilis diagnosis.	**Annotation:** Correct<br>**Comment:** None
**Final Answer:** Based on the image comprehension, recalled medical knowledge, and step-by-step reasoning, the most likely diagnosis for this patient is E.Malignant syphilis.	**Annotation:** Correct

Q22. NEJM Image Challenge 5/11/23

A 55-year-old man who had been admitted to the intensive care unit had a sudden-onset vesicular rash appear across his trunk and arms, as shown. He had undergone a small-bowel resection; his post-operative course had been complicated by sepsis, fevers, and respiratory failure. On exam, he was wrapped tightly in blankets. The vesicles did not rupture with light palpation but broke when more pressure was applied. What is the diagnosis?

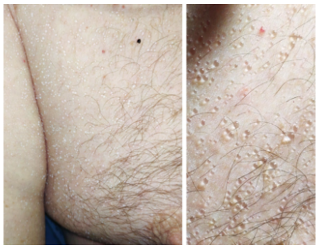

A. Drug eruption
B. Dyshidrotic eczema
C. Herpes zoster due to virus reactivation
D. Miliaria crystallina
E. Pityrosporum folliculitis

Correct Answer: Miliaria crystallina
Explanation: The correct answer is miliaria crystallina. After 24 hours, the rash abated without intervention. Miliaria crystallina is a benign, self-resolving skin condition that results from superficial obstruction of sweat ducts in the stratum corneum. It is most commonly seen in neonates but is also observed in adults exposed to high heat and humidity. A recommendation was given to avoid tight blankets in order to minimize sweating and skin irritation.

GPT-4V Answer

**Image Comprhension:** The image displays a close-up view of a person's skin, specifically areas of the trunk and arms. There are multiple tiny, clear, and blister-like vesicles spread across the skin. These vesicles are closely packed together and seem to be filled with a clear fluid. The surrounding skin appears slightly reddened in some areas, and there is visible body hair.	**Annotation:** Correct<br>**Comment:** None
**Recall of Medical Knowledge:** Drug eruption: Typically presents as a generalized rash that may vary in appearance. It could be maculopapular, urticarial, or vesicular. It can occur as a reaction to medications, often beginning within days to weeks after starting a new drug.<br>Dyshidrotic eczema: Characterized by small, itchy blisters on the edges of fingers, toes, palms, and soles of the feet. The blisters are often filled with fluid.<br>Herpes zoster due to virus reactivation: This is a reactivation of the varicella-zoster virus, which causes chickenpox. It presents as a painful rash that usually appears as a stripe of blisters wrapping around one side of the torso, but can also occur on other parts of the body.<br>Miliaria crystallina: Also known as "sweat rash," this condition is characterized by small, clear, and superficial blisters resulting from sweat trapped under the skin. It is commonly seen in conditions where patients are overheated or have a high fever. It's the mildest form of heat rash and doesn't produce the itchiness commonly associated with other forms.<br>Pityrosporum folliculitis: Caused by a yeast (fungus) infection of the hair follicles. It presents as itchy, acne-like breakouts, commonly appearing on the chest, back, and shoulders.	**Annotation:** Correct<br>**Comment:** None
**Step-by-step Reasoning:** Given the clinical scenario and the image's appearance:<br>The rash appears as tiny, clear vesicles, which aligns with the presentation of miliaria crystallina. The patient was in the intensive care unit, likely immobilized, and wrapped tightly in blankets, creating an environment conducive to overheating and sweat accumulation. The post-operative course with sepsis, fevers, and respiratory failure would have made the patient prone to sweating excessively. The absence of the typical distribution pattern of herpes zoster (which often follows a dermatomal distribution) and the lack of mention of pain or significant discomfort makes herpes zoster less likely. The rash does not resemble the typical presentations of drug eruption, dyshidrotic eczema, or Pityrosporum folliculitis.	**Annotation:** Correct<br>**Comment:** None
**Final Answer:** Based on the image and the recalled medical knowledge, the most likely diagnosis is D. Miliaria crystallina.	**Annotation:** Correct

Q23. NEJM Image Challenge 5/4/23

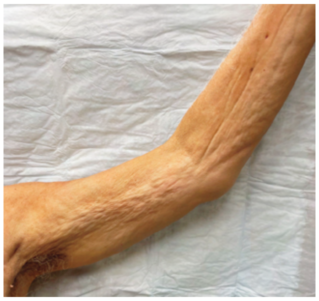

A 66-year-old man presented with an 8-month history of painful skin tightening and swelling of his arms and legs. The symptoms had progressed on glucocorticoids and methotrexate. On physical exam, there was symmetric, woody induration of the trunk, arms, and legs, sparing the hands and feet. The involved skin had a dimpled appearance. Elevation of the arms resulted in visible indentions along the course of superficial veins. Elbow contractures were also noted. What is the most likely diagnosis?

A. Eosinophilic fasciitis
B. Myxedema
C. Scleredema
D. Scleroderma
E. Scleromyxedema

Correct Answer: Eosinophilic fasciitis
Explanation: The visible indentations along the courses of superficial veins were consistent with "groove sign," a finding seen in eosinophilic fasciitis. Laboratory studies were notable for absolute eosinophilia. A skin biopsy of the left arm was performed, and histopathological examination showed a perivascular and interstitial inflammatory infiltrate of lymphocytes and eosinophils in the deep reticular dermis and subcutis. The patient was treated with intravenous methylprednisolone, oral mycophenolate mofetil, and intravenous immune globulin.

GPT-4V Answer

**Image Comprhension:** The image displays an arm, potentially belonging to an older adult, on a white backdrop. The skin on the arm exhibits signs of thickening, with a rough, dimpled appearance, akin to the "peau d'orange" or "orange peel" texture. Additionally, the skin appears to be tight and possibly indurated. There are visible indentations which could correspond to the described superficial veins. No overt lesions, rash, or discoloration is visible beyond the generalized thickening.	**Annotation:** Correct<br>**Comment:** None
**Recall of Medical Knowledge:** Eosinophilic fasciitis: A rare inflammatory condition that affects the fascia (connective tissue) underneath the skin. It leads to skin thickening and may look similar to scleroderma but typically spares the hands and feet. Myxedema: Caused by severe hypothyroidism, it can lead to swelling of the skin and underlying tissues. The skin becomes thick and puffy, especially on the face. Scleredema: A rare skin condition that leads to thickening of the skin on the upper back and neck primarily. It's often associated with diabetes and occurs suddenly. Scleroderma: A group of autoimmune diseases causing the skin and connective tissues to become tightened and thickened. There are two types: localized and systemic. In the systemic form, internal organs might be affected. Scleromyxedema: Another rare condition characterized by the presence of lichenoid papules, thickened skin, and sometimes associated with paraproteinemia.	**Annotation:** Correct<br>**Comment:** None
**Step-by-step Reasoning:** Scleroderma primarily affects the skin but can also affect internal organs. The image does not show the "salt and pepper" skin commonly associated with systemic scleroderma. Also, scleroderma typically affects the hands, causing them to become thickened and claw-like, which is inconsistent with the description that hands are spared. Myxedema often leads to thickening, especially on the face, and not specifically the arms and legs. The description does not mention facial changes, so it's less likely. Scleredema is commonly associated with diabetes and usually affects the upper back and neck. The description does not mention diabetes or specific neck/back involvement, making this diagnosis less likely. Scleromyxedema causes lichenoid papules and skin thickening, but the image does not show papules. Eosinophilic fasciitis presents with skin thickening, especially on the arms and legs, spares the hands and feet, and leads to "woody" induration. The physical findings described in the scenario, especially the sparing of the hands and feet, are consistent with this condition.	**Annotation:** Correct<br>**Comment:** None
**Final Answer:** Based on the given description and the findings in the image, the most likely diagnosis is: A. Eosinophilic fasciitis.	**Annotation:** Correct

Q24. NEJM Image Challenge 4/27/23

A 75-year-old woman presented to the infectious diseases clinic with a 10-day history of painful lesions on the right hand and forearm. An aspiration of the hand lesion was performed. Bacterial culture of the aspirate grew filamentous, gram-positive, acid-fast branching rods. Which of the following activities likely preceded development of the lesions?

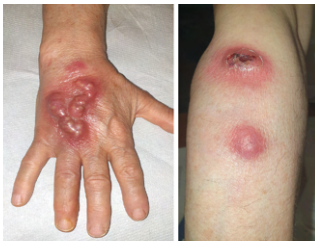

A. Cleaning a fish tank
B. Gardening
C. Getting bitten by a sandfly
D. Handling feline waste
E. Petting an armadillo

Correct Answer: Gardening
Explanation: The nodules had first appeared on the back of her hand at the site of a thorn puncture she had sustained while gardening. The lesions had spread up the hand along a red streak and then had appeared on the forearm. The culture was subsequently identified as Nocardia brasiliensis, and a diagnosis of sporotrichoid nodular lymphangitis was made. After a course of trimethoprim–sulfamethoxazole, the lesions resolved.

GPT-4V Answer	
**Image Comprhension:** The image showcases two distinct views of a human hand and forearm. On the hand, there are painful-looking, reddened, raised lesions with a shiny and blister-like appearance. The forearm displays a larger, circular lesion that is red at its perimeter with a central area that looks necrotic and crusty.	**Annotation:** Partially correct<br>**Comment:** They are not blisters, they are nodules/lumps.
**Recall of Medical Knowledge:** The mentioned bacterial culture result points to a genus of bacteria called "Nocardia." Nocardia infections often occur when the skin is broken and comes in direct contact with soil or water contaminated with the bacteria. It can lead to skin infections that cause abscesses or pus-filled lumps and may also spread to other parts of the body.<br>A. Cleaning a fish tank: Can lead to infections like Mycobacterium marinum, but not typically Nocardia. B. Gardening: Direct contact with soil can introduce pathogens, including Nocardia. C. Getting bitten by a sandfly: Causes leishmaniasis. D. Handling feline waste: Can result in toxoplasmosis or cat scratch disease. E. Petting an armadillo: Associated with the spread of Hansen's disease or leprosy.	**Annotation:** Partially correct<br>**Comment:** Feline waste itself is not a direct source for cat scratch disease.
**Step-by-step Reasoning:** Given the clues provided:<br>The clinical presentation of the lesions on the hand and forearm. The bacterial culture growing filamentous, gram-positive, acid-fast branching rods (indicative of Nocardia). The most likely source of the Nocardia infection is direct contact with soil, as Nocardia species are found in soil worldwide.<br>Considering the options:<br>A. Mycobacterium marinum is more commonly associated with fish tank cleaning. B. Gardening provides direct exposure to soil, which is a common source of Nocardia. C. Sandflies cause leishmaniasis, not Nocardia infections. D. Handling feline waste can lead to other infections, but not Nocardia. E. Armadillos are linked to leprosy, not Nocardia.	**Annotation:** Correct<br>**Comment:** None
**Final Answer:** Given the above, the most probable activity that preceded the development of the lesions is: B. Gardening.	**Annotation:** Correct

Q25. NEJM Image Challenge 4/20/23

A 60-year-old left-handed woman presented to the emergency department with pain in her left forearm. The arm was swollen and tender, especially with passive pronation and supination. The overlying skin was intact, and the results of neurovascular examination were normal. Radiographs of the left forearm were performed (upper image, anteroposterior view; lower image, lateral view). The findings should raise concern for which contributory factor?

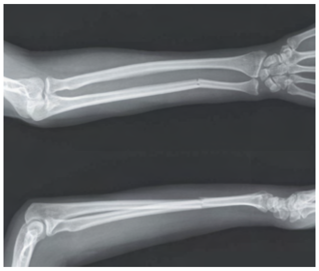

A. Assault
B. Fall
C. Malignancy
D. Osteoporosis
E. Repetitive strain

Correct Answer: Assault

Explanation: The correct answer is assault. The radiographs confirm an isolated ulnar shaft fracture, also called a "nightstick fracture." This injury results from blunt force trauma to the forearm, classically occurring when the upper arm is raised in self-defense against an attack to the head or torso. In this case, the trauma was due to intimate partner violence. Comprehensive assessments of the safety and mental health of the patient were performed, and social services were offered. The patient underwent open reduction and internal fixation of the fracture, followed by splinting, although this type of injury may be managed non-operatively in some cases.

GPT-4V Answer	
**Image Comprhension:** The images presented are radiographs (X-rays) of the left forearm. There are two views provided: anteroposterior (front-to-back) and lateral (side view). In the anteroposterior view, there seems to be a fracture in the midshaft of the radius bone (one of the two bones of the forearm) with slight angulation. The fracture does not appear to be comminuted (broken into multiple pieces) but there is a visible break in the continuity of the bone. The lateral view supports the findings from the anteroposterior view.	**Annotation:** Correct<br>**Comment:** None
**Recall of Medical Knowledge:** The bones in older adults can be prone to fractures due to decreased bone density. This condition is known as osteoporosis. Malignancies can cause a pathologic fracture, but they usually present with a lytic (bone-eating) or blastic (bone-forming) lesion on the radiograph. Repetitive strain typically causes stress fractures, which usually appear as hairline fractures and are often difficult to spot on X-rays. Assaults and falls can cause fractures depending on the force and nature of the trauma. The nature of the fracture can sometimes suggest the mechanism of injury. The presentation of swelling, pain, and tenderness, especially with pronation and supination, is consistent with a forearm fracture.	**Annotation:** Correct<br>**Comment:** None
**Step-by-step Reasoning:** Given the age of the patient and the type of fracture visualized on the X-ray:<br>The absence of any evident lytic or blastic lesions on the radiograph makes malignancy less likely. The fracture appears to be more than just a hairline fracture, which makes repetitive strain an unlikely cause. The nature of the fracture doesn't provide specific indicators to suggest assault or fall directly. Both can lead to such fractures. Considering the patient's age (60 years), osteoporosis is a likely contributory factor that can make bones more susceptible to fractures even with minimal trauma.	**Annotation:** Incorrect<br>**Comment:** Agree with GPT-4V that the patient's demographic puts osteoporosis as the most likely contributory factor. However, the nature of the fracture is isolated to the ulnar shaft and its radiographic presentation is pathognomonic for a nightstick fracture suggesting trauma.
**Final Answer:** Based on the information provided and the X-ray findings, the most concerning contributory factor for this fracture in a 60-year-old woman would be D.Osteoporosis.	**Annotation:** Incorrect

Q26. NEJM Image Challenge 4/13/23

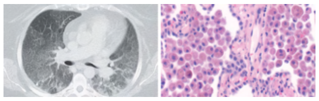

A 34-year-old woman with tobacco use disorder was referred to the pulmonary clinic for worsening dyspnea and dry cough. She had chronic hypoxemic respiratory failure of unknown cause, requiring supplemental oxygen. Computed tomography of the chest showed diffuse ground-glass opacities and areas of peripheral consolidation but no basilar reticulations or honeycombing. Bronchoalveolar lavage was not diagnostic, so surgical lung biopsy was performed. Histopathological analysis showed extensive alveolar filling with pigment-laden macrophages. What is the most likely diagnosis?

A. Desquamative interstitial pneumonia
B. Diffuse alveolar hemorrhage
C. Idiopathic pulmonary fibrosis
D. Lipoid pneumonia
E. Pulmonary alveolar proteinosis

Correct Answer: Desquamative interstitial pneumonia
Explanation: These findings were consistent with desquamative interstitial pneumonia, a rare form of idiopathic interstitial pneumonia that is highly associated with cigarette smoking. Treatment with a smoking-cessation program and a tapering dose of glucocorticoids was initiated.

GPT-4V Answer

Image Comprhension: The image on the left appears to be a computed tomography (CT) scan of the chest, showing a lung cross-section with diffuse ground-glass opacities. The presence of these opacities suggests inflammation or fluid in the alveolar spaces. The image on the right appears to be a histopathological slide, showing lung tissue at a microscopic level. The pink-stained cells are suggestive of macrophages, and the presence of dark pigmentation within these cells suggests that they are laden with some type of pigment.

Annotation: Partially correct
Comment: None

Recall of Medical Knowledge: Desquamative interstitial pneumonia (DIP): Typically associated with smoking, DIP is characterized by the accumulation of pigment-laden macrophages within the alveoli. CT scans of the chest in patients with DIP usually reveal ground-glass opacities.
Diffuse alveolar hemorrhage: This condition is characterized by the presence of blood within the alveoli. It would typically show red blood cells on histopathological analysis and might present with hemoptysis (coughing up blood) in the patient's symptoms.
Idiopathic pulmonary fibrosis: A chronic, progressive lung disease characterized by fibrosis of the lung tissue. On CT, it would typically show a honeycombing pattern, which was not mentioned in the provided information.
Lipoid pneumonia: Caused by the accumulation of lipids in the alveoli, often due to aspiration of oil-based substances. Histopathology would typically show lipid-laden macrophages.
Pulmonary alveolar proteinosis: Characterized by the accumulation of a proteinaceous material in the alveoli. Histopathology would show periodic acid-Schiff (PAS) positive material filling the alveoli.

Annotation: Correct
Comment: None

Step-by-step Reasoning: Given the history of tobacco use, DIP becomes a differential, especially with the presence of pigment-laden macrophages and ground-glass opacities on the CT scan.
The absence of red blood cells in the histopathological analysis makes diffuse alveolar hemorrhage less likely.
The CT scan does not show honeycombing, which makes idiopathic pulmonary fibrosis an unlikely diagnosis.
While lipoid pneumonia also shows lipid-laden macrophages, the patient's history does not suggest any aspiration events of oil-based substances, making it less likely.
The absence of PAS-positive material in the alveoli makes pulmonary alveolar proteinosis an unlikely diagnosis.

Annotation: Correct
Comment: None

Final Answer: Given the presented findings and the recall of medical knowledge, the most likely diagnosis for this patient is A. Desquamative interstitial pneumonia (DIP).

Annotation: Correct

Q27. NEJM Image Challenge 4/6/23

An 87-year-old man with chronic kidney disease, hypertension, and atrial fibrillation presented to the emergency department with a 3-day history of constipation and lower abdominal pain. Physical examination was notable for abdominal distention and tenderness to palpation of the right lower quadrant without rebound or guarding. A plain radiograph of the abdomen is shown. What is the most likely diagnosis?

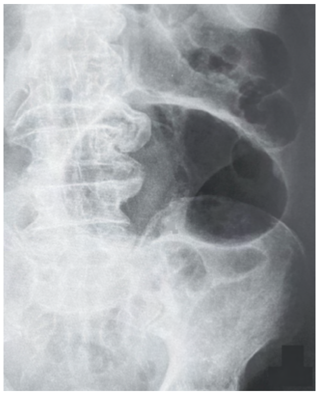

A. Cecal volvulus
B. Constipation
C. Hiatal hernia
D. Ogilvie's Syndrome (acute colonic pseudo-obstruction)
E. Small bowel obstruction

Correct Answer: Cecal volvulus

Explanation: The plain radiograph of the abdomen showed an air-filled loop of large bowel resembling a coffee bean, a finding that may be seen in cecal or sigmoid volvulus. Computed tomography of the abdomen was not available at the remote island hospital to which the patient presented. He was taken urgently to the operating room, and a cecal volvulus without signs of bowel compromise was identified and successfully detorsed. Owing to intraoperative hemodynamic instability, a cecopexy and cecostomy were performed rather than an ileocecectomy or right colectomy. The patient's postoperative course was uncomplicated. At 6 months of follow-up, he was doing well and declined ostomy reversal at that time.

GPT-4V Answer

**Image Comprhension:** The radiograph depicts a large dilated loop of bowel located in the upper abdominal region, which is suggestive of colonic distension. The distension seems to be most prominent in the right side of the abdomen. There are no clear radiographic signs of mechanical obstruction, such as multiple air-fluid levels or dilated small bowel loops, which would typically be seen with small bowel obstruction.	**Annotation:** Partially correct<br>**Comment:** The colonic distention is mid-line to left sided, not right sided. Dilated bowel is often a sign of mechanical obstruction inaddition to air-fluid levels. I believe GPT is referring to small bowel obstruction, so it failed to connect that large and small bowel both can be obstructed.
**Recall of Medical Knowledge:** Cecal volvulus: This is a condition where the cecum (beginning of the large intestine) twists around its mesentery, potentially causing intestinal obstruction. Radiographic findings might show a "coffee bean" sign. Constipation: A common condition in the elderly, it typically presents with hard stools and infrequent bowel movements, but is less likely to cause the marked distension seen in the image. Hiatal hernia: This is a condition where a portion of the stomach pushes up into the chest through the diaphragm. It wouldn't appear as distended bowel in the abdomen on an X-ray. Ogilvie's Syndrome (acute colonic pseudo-obstruction): This condition involves massive dilatation of the colon in the absence of mechanical obstruction. It often occurs in the elderly or critically ill and can be due to a variety of underlying conditions, including electrolyte imbalances, recent surgery, and certain medications. Small bowel obstruction: This usually presents with multiple dilated loops of small bowel and air-fluid levels on radiography.	**Annotation:** Correct<br>**Comment:** None
**Step-by-step Reasoning:** The radiograph does not show the classic "coffee bean" sign associated with cecal volvulus. There is no evidence of small bowel obstruction, as no dilated small bowel loops or air-fluid levels are seen. A hiatal hernia would not cause the findings seen on this radiograph. Given the patient's age, comorbidities, and presentation, along with the radiographic findings, the most likely diagnosis is Ogilvie's Syndrome. The massive dilation of the colon without a mechanical cause fits this diagnosis.	**Annotation:** Incorrect<br>**Comment:** The X-ray does in fact show a "coffee bean" sign. There is dilation of the colon, but it is more focal than would be expected for Ogilvie's syndrome.
**Final Answer:** Based on the above, the answer is: D. Ogilvie's Syndrome (acute colonic pseudo-obstruction).	**Annotation:** Incorrect

Q28. NEJM Image Challenge 3/30/23

A pregnant 33-year-old woman at 35 weeks' gestation presented with redness on both palms without pain and pruritus. She was otherwise asymptomatic. The skin changes had first appeared during the second trimester of pregnancy. Skin examination was notable for mottled, blanching erythema across the palms and fingers of both hands. What is the diagnosis?

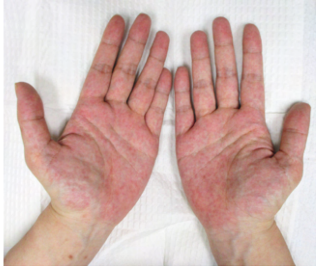

A. Atopic eruption of pregnancy
B. Intrahepatic cholestasis of pregnancy
C. Palmar erythema of pregnancy
D. Polymorphic eruption of pregnancy
E. Tinea manuum

Correct Answer: Palmar erythema of pregnancy
Explanation: The correct answer is palmar erythema of pregnancy, one of the physiologic vascular skin changes of pregnancy. No specific treatment is indicated, and the condition typically resolves within 2 weeks after parturition, although full resolution may take up to 2 months.

GPT-4V Answer

**Image Comprhension:** The image displays both palms of an individual. The palms show a mottled, blanching erythema which is most prominent over the thenar and hypothenar eminences, and extending to the fingers. The skin appears to be intact without any breaks, vesicles, or crust. The redness is not accompanied by any discernible scaling or other secondary skin changes.	**Annotation:** Correct<br>**Comment:** None
**Recall of Medical Knowledge:** Atopic eruption of pregnancy (AEP): This is a pruritic condition that occurs during pregnancy. It often involves the trunk and limbs, presenting as eczematous lesions. Intrahepatic cholestasis of pregnancy (ICP): It's characterized by pruritus, especially on the palms and soles. It can be associated with elevated bile acid levels. ICP can have adverse fetal outcomes. Palmar erythema of pregnancy: This is a benign condition where the palms turn red, often due to increased estrogen levels during pregnancy. It typically affects the thenar and hypothenar eminences. Polymorphic eruption of pregnancy (PEP): This is a pruritic condition characterized by red, raised areas that can form larger plaques, mainly on the abdomen and sparing the umbilical region. Tinea manuum: It's a fungal infection of the hand, usually presenting with scaling and can be unilateral.	**Annotation:** Correct<br>**Comment:** None
**Step-by-step Reasoning:** The described skin changes began during the second trimester of pregnancy, which provides a temporal link to the pregnancy. There's no mention of pruritus, which rules out conditions like ICP and AEP which are typically itchy. The rash is bilateral and does not have features of scaling, making tinea manuum less likely. Polymorphic eruption of pregnancy primarily affects the abdomen and is pruritic, making it an unlikely diagnosis. The distribution and appearance of the erythema, especially the prominence in the thenar and hypothenar regions, along with its onset during pregnancy and lack of other symptoms, strongly suggest Palmar erythema of pregnancy.	**Annotation:** Correct<br>**Comment:** None
**Final Answer:** Answer: C.Palmar erythema of pregnancy.	**Annotation:** Correct

Q29. NEJM Image Challenge 3/23/23

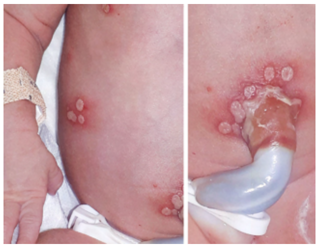

A baby girl was noted to have vesicular skin lesions immediately after spontaneous vaginal delivery at 32 weeks' gestation. Her 37-year-old mother had premature rupture of membranes 12 days before the delivery. On physical examination, clusters of vesicles were seen on the infant's torso (left), periumbilical region (right), and pharyngeal, nasal, and conjunctival mucosa. No lesions were seen on the mother's genitals or the placenta. What is the most likely diagnosis?

A. Bullous impetigo
B. Epidermolysis bullosa
C. Molluscum contagiosum
D. Neonatal herpes simplex virus
E. Transient pustular melanosis

Correct Answer: Neonatal herpes simplex virus
Explanation: The correct answer is neonatal herpes simplex virus. Polymerase-chain-reaction (PCR) testing of samples obtained from the infant's skin, conjunctivae, pharynx, and blood were positive for herpes simplex virus type 1 (HSV-1) and a diagnosis of neonatal HSV-1 infection that was confined to the skin, eyes, and mucosa was made. The mother reported no history of previous HSV-1 infection. However, a postpartum endocervical PCR assay was positive for HSV-1. In this case, a primary maternal infection late in pregnancy was thought to have led to ascending intrauterine infection and possibly preterm labor after prolonged rupture of membranes. Treatment with acyclovir was given. At 2 years of age, the child had had no recurrences and was developmentally normal.

GPT-4V Answer

**Image Comprhension:** The image displays close-up views of a newborn baby's skin, particularly focusing on the torso and periumbilical regions. Notably: On the left side of the image, there are clusters of vesicles on the baby's torso. These vesicles appear round, with a clear or slightly cloudy fluid inside, and are surrounded by erythematous (red) skin. On the right side, there's a more magnified view of vesicles located in the periumbilical region. This image offers a clearer perspective of the vesicles, which are filled with a clear fluid. Adjacent to these vesicles, there's what appears to be a medical tube or catheter.	**Annotation:** Partially correct<br>**Comment:** Not medical catheter but rather baby's umbilical stump
**Recall of Medical Knowledge:** Bullous impetigo: Caused by Staphylococcus aureus or Streptococcus pyogenes, it presents with large, flaccid bullae filled with clear yellow fluid. When the bullae rupture, it leaves a thin brown crust. Epidermolysis bullosa: A group of genetic conditions causing the skin to become very fragile and blister easily. Molluscum contagiosum: A viral infection causing firm, round, painless bumps on the skin. Neonatal herpes simplex virus (HSV): An infection transmitted from an infected mother to her baby during delivery. It can present with skin, eye, and mouth (SEM) involvement, with vesicles in these areas. Central nervous system and disseminated diseases are other presentations. Transient pustular melanosis: Presents with small vesicopustules over a hyperpigmented base, primarily seen in African-American neonates. The vesicles resolve quickly, leaving hyperpigmented macules that disappear after several months.	**Annotation:** Correct<br>**Comment:** None
**Step-by-step Reasoning:** Considering the image shows vesicular lesions, this can rule out transient pustular melanosis as it presents with pustules and not vesicles. Bullous impetigo and epidermolysis bullosa can cause blistering in neonates. However, the absence of yellow fluid or brown crust makes bullous impetigo less likely. Additionally, the widespread distribution of vesicles, especially on mucosal surfaces, is not typical for epidermolysis bullosa. Molluscum contagiosum presents with firm, round bumps and not vesicles filled with clear fluid. Neonatal HSV can manifest with vesicles on the skin, eyes, and mucosal surfaces. Given the presentation and the history provided (especially the premature rupture of membranes), neonatal HSV appears to be the most likely diagnosis.	**Annotation:** Partially correct<br>**Comment:** Mucosal surface not depicted here
**Final Answer:** Answer: D.Neonatal herpes simplex virus.	**Annotation:** Correct

Q30. NEJM Image Challenge 3/16/23

A 12-year-old boy with a history of obesity presented with a 2-week history of limping and dull pain in the right hip. The symptoms had begun after he had slipped and fallen at school. On physical exam, active and passive ranges of motion of the right hip were limited by pain. Radiographs of the pelvis were performed (frog-leg lateral view is shown). What is the most likely diagnosis?

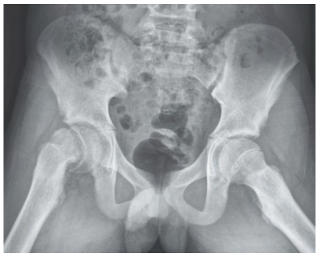

A. Chondrosarcoma
B. Legg-Calve-Perthes disease
C. Osgood-Schlatter disease
D. Osteoarthritis
E. Slipped capital femoral epiphysis

Correct Answer: Slipped capital femoral epiphysis
Explanation: The correct answer is slipped capital femoral epiphysis. This is the most common hip disorder in teenagers. It occurs when the proximal femur displaces anterolaterally and superiorly, which gives an appearance resembling a scoop of ice cream slipping off a cone. The condition is associated with obesity but not typically preceding trauma, as occurred in this case.

GPT-4V Answer	
**Image Comprhension:** The image is a radiograph (X-ray) of the pelvis, showing both hips in a frog-leg lateral view. On the right hip, there is a noticeable displacement of the femoral head (the ball part of the ball-and-socket joint of the hip) relative to the femoral neck. The displacement appears to be posterior and medial, meaning it's shifted backward and toward the midline of the body. There is no clear sign of any tumors, arthritic changes, or other bone abnormalities.	**Annotation:** Correct<br>**Comment:** None
**Recall of Medical Knowledge:** Chondrosarcoma: A type of bone cancer that usually affects adults, especially older adults. Legg-Calve-Perthes disease: A childhood condition where blood supply to the femoral head becomes temporarily interrupted, leading to bone death. It typically presents in children aged 4-8 and causes a gradual onset of symptoms. Osgood-Schlatter disease: Inflammation of the patellar ligament at the tibial tuberosity. It's seen in growing adolescents and presents as knee pain, not hip pain. Osteoarthritis: A degenerative joint disease commonly seen in older adults. It's unlikely in children unless there's a history of trauma or a predisposing condition. Slipped capital femoral epiphysis (SCFE): A condition where the femoral head slips off the neck along the growth plate. It usually occurs during periods of rapid growth in adolescence, especially in obese children. The classic presentation is a child with a limp and pain in the hip, knee, or groin.	**Annotation:** Correct<br>**Comment:** None
**Step-by-step Reasoning:** Chondrosarcoma is unlikely as the patient is only 12, and there's no tumor visible on the radiograph. Legg-Calve-Perthes disease generally affects a younger age group and does not align with a traumatic event like a fall. Osgood-Schlatter disease presents with knee pain, not hip pain. Osteoarthritis is unlikely given the child's age and the absence of arthritic changes on the X-ray. Slipped capital femoral epiphysis (SCFE) is the most likely diagnosis. The history of a fall, the obesity, the age group, the symptoms described, and the radiographic findings all point to SCFE. The image shows a posterior and medial displacement of the femoral head, which is consistent with SCFE.	**Annotation:** Correct<br>**Comment:** None
**Final Answer:** Based on the above reasoning, the answer is: E. Slipped capital femoral epiphysis.	**Annotation:** Correct

Q31. NEJM Image Challenge 3/9/23

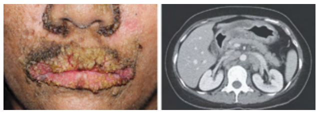

A 30-year-old woman presented with a 3-month history of progressive skin lesions on her face. On physical examination, there were hyperpigmented, yellow, papillomatous papules and plaques on the face (left). Hyperpigmented velvety plaques were also seen on the skin of the neck, groin and axillae. She also reported new-onset heartburn, fatigue, and unintentional 15-kg weight loss. A skin biopsy of the facial lesion showed epidermal papillomatosis, acanthosis, hyperkeratosis, and negative staining for human papillomavirus. A computed tomography (CT) of the abdomen and pelvis was performed (right). What is the most likely diagnosis?

A. Erythrasma
B. Ichthyosis hystrix
C. Malignant acanthosis nigricans
D. Pellagra (Vitamin B3 deficiency)
E. Pemphigus vegetans

Correct Answer: Malignant acanthosis nigricans
Explanation: The correct answer is malignant acanthosis nigricans. This is a paraneoplastic dermopathy involving the sudden onset of velvety plaques on cutaneous and mucosal surfaces. These changes are more extensive and rapidly worsening than those in benign acanthosis nigricans. The CT of the abdomen and pelvis showed thickening of the gastric body and a solid mass in the right ovary. A gastric biopsy showed mucin-rich signet-ring-cell gastric adenocarcinoma, confirming a diagnosis of gastric adenocarcinoma with ovarian metastasis.

GPT-4V Answer

Image Comprhension: The left image shows a close-up view of a part of a person's face. The facial region depicted has hyperpigmented areas with yellow, papillomatous papules and plaques that appear raised and rough. The right image is a CT scan of the abdomen and pelvis. It displays multiple hypo-attenuating lesions within the liver, suggesting potential metastatic disease or multiple liver lesions.

Annotation: Correct
Comment: None

Recall of Medical Knowledge: Erythrasma: This is a superficial skin infection caused by the bacterium Corynebacterium minutissimum. It usually presents as pink or brown patches, primarily in skin folds, and can be identified under ultraviolet light due to coral-red fluorescence.
Ichthyosis hystrix: This is a rare form of ichthyosis, a skin disorder characterized by dry, rough, and scaly skin. It involves the formation of large, spiky hyperkeratoses on the skin, but does not typically result in liver lesions.
Malignant acanthosis nigricans: This is a skin condition characterized by hyperpigmented, velvety plaques, especially in flexural areas. It is often associated with an underlying malignancy, most commonly gastrointestinal adenocarcinomas.
Pellagra (Vitamin B3 deficiency): Pellagra results from a deficiency of niacin (vitamin B3). It is characterized by the four D's: dermatitis, diarrhea, dementia, and death. The skin manifestations include photosensitive dermatitis.
Pemphigus vegetans: This is a variant of pemphigus vulgaris, a blistering skin condition. It presents with vegetating, pustular lesions, primarily in the folds of the skin.

Annotation: Correct
Comment: None

Step-by-step Reasoning: The clinical presentation and the images provided show hyperpigmented, papillomatous, and velvety plaques, especially in the facial region, neck, groin, and axillae. This matches the description of acanthosis nigricans.
The significant weight loss, the onset of symptoms, and the appearance of multiple liver lesions on the CT scan strongly suggest an underlying malignancy. Given this context, benign acanthosis nigricans (which is associated with conditions like obesity and insulin resistance) is less likely.
Among the options provided, only "Malignant acanthosis nigricans" aligns with both the skin presentation and the potential underlying malignancy, especially gastrointestinal adenocarcinomas that could lead to liver metastases as indicated by the CT image.

Annotation: Correct
Comment: None

Final Answer: Answer: C. Malignant acanthosis nigricans.

Annotation: Correct

Q32. NEJM Image Challenge 3/2/23

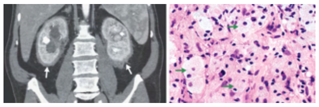

A 62-year-old man undergoing abdominal ultrasonography for the evaluation of gallstones was found to have a retroperitoneal mass. A physical examination and the results of routine laboratory studies were normal. A computed tomographic (CT) urogram was completed (left), and a subsequent positron-emission tomography–CT showed no hypermetabolic activity. He underwent stent placement in both ureters, and a core-biopsy of the perinephric soft tissue was obtained. Hematoxylin and eosin staining of the specimen is shown (right). What is the diagnosis?

A. Erdheim–Chester disease
B. IgG4-related disease
C. Langerhans cell histiocytosis
D. Lymphoma
E. Sarcoidosis

Correct Answer: Erdheim–Chester disease
Explanation: The correct answer is Erdheim–Chester disease. Erdheim–Chester disease is a non–Langerhans-cell histiocytosis that typically manifests with sclerotic lesions in the long bones. It is also associated with extraosseous findings, including infiltration of the retroperitoneal perinephric tissue resulting in a "hairy kidney" appearance on cross-sectional imaging, as seen in this patient. On histopathological examination of the biopsy specimen, diffuse infiltration with pale-staining histiocytes was noted (arrows).

GPT-4V Answer

**Image Comprhension:** The image provided is a split image. The left half appears to be a CT urogram showing a retroperitoneal mass. There are arrows indicating specific parts of the image, possibly highlighting the mass or abnormal areas. The right half is a hematoxylin and eosin (HE) stained histological slide showing cells and tissue structures. There are arrows pointing towards specific cells or structures that seem to be of interest or represent abnormal findings.	**Annotation:** Correct<br>**Comment:** None
**Recall of Medical Knowledge:** Erdheim–Chester disease is a rare form of non-Langerhans cell histiocytosis, and it primarily affects the long bones. It can also affect the kidneys, but its typical histological appearance includes foamy histiocytes and Touton giant cells.<br>IgG4-related disease is an immune-mediated condition that can affect various organs. The hallmark histological finding includes lymphoplasmacytic infiltration with increased numbers of IgG4-positive plasma cells, storiform fibrosis, and obliterative phlebitis.<br>Langerhans cell histiocytosis (LCH) involves the proliferation of Langerhans cells, which are specialized immune cells. On histology, Langerhans cells have a distinctive appearance, including folded or grooved nuclei. The presence of Birbeck granules on electron microscopy is diagnostic, though they are not visible on regular HE stains.<br>Lymphoma is a malignancy of lymphocytes and can present as masses in various parts of the body, including the retroperitoneum. On histology, the appearance will depend on the subtype of lymphoma, but it generally involves the proliferation of abnormal lymphocytes.<br>Sarcoidosis is a granulomatous disease of unknown etiology that can affect multiple organs. The characteristic histological finding is non-caseating granulomas.	**Annotation:** Partially correct<br>**Comment:** None
**Step-by-step Reasoning:** Given the provided information and the histological image, the following observations and deductions can be made:<br>There is no obvious presence of foamy histiocytes or Touton giant cells which would indicate Erdheim-Chester disease. The image doesn't seem to show the hallmark findings of IgG4-related disease such as storiform fibrosis or obliterative phlebitis. The image lacks the distinctive Langerhans cells with folded or grooved nuclei that would indicate LCH. Lymphomas can be varied in their histological presentation, but the provided image does not clearly resemble the most common lymphoma presentations. The histology image seems to show granulomatous structures, which are characteristic of sarcoidosis.	**Annotation:** Correct<br>**Comment:** None
**Final Answer:** Based on the provided information, the image comprehension, and the recall of medical knowledge, the most likely diagnosis is: E. Sarcoidosis.	**Annotation:** Incorrect

Q33. NEJM Image Challenge 2/23/23

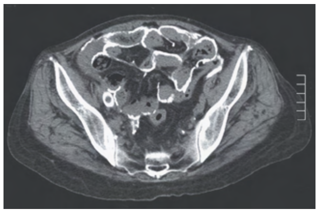

A 52-year-old woman with end-stage kidney disease that was being managed with peritoneal dialysis presented with a 1-month history of bloody dialysate. She had had 3 episodes of bacterial peritonitis in the past 12 years. Physical examination and laboratory studies were unremarkable. Computed tomography of the abdomen was performed. What is the most likely diagnosis?

A. Calciphylaxis
B. Encapsulating peritoneal sclerosis
C. Fungal peritonitis
D. Peritoneal carcinomatosis
E. Tuberculous peritonitis

Correct Answer: Peritoneal carcinomatosis
Explanation: CT scan of the abdomen showed extensive calcification along the visceral and parietal peritoneum. Exploratory laparoscopy showed a markedly thickened parietal peritoneum covered in chalklike deposits. Histopathological examination showed fibrosis and calcification of the parietal peritoneum consistent with encapsulating peritoneal sclerosis with extensive calcifications. The patient's peritoneal catheter was removed and maintenance hemodialysis initiated.

GPT-4V Answer	
**Image Comprhension:** The image appears to be an axial cross-sectional CT (computed tomography) scan of the lower abdomen and pelvis. There is evidence of thickened peritoneal lining and some fluid collections. The peritoneal thickening seems to be quite significant and is seen encasing the bowel loops. The thickening appears to have a slightly irregular pattern. There are no overt calcifications, abscesses, or masses seen in the peritoneal cavity.	**Annotation:** Partially correct<br>**Comment:** There are significant overt calcifications in the bowel wall. GPT4 states that there are none.
**Recall of Medical Knowledge:** Calciphylaxis is characterized by calcification of small and medium-sized blood vessels, leading to skin necrosis. While it can be seen in patients with end-stage renal disease, the primary imaging findings would be vascular calcifications, which aren't visible here. Encapsulating peritoneal sclerosis (EPS) is a rare but serious complication of peritoneal dialysis, leading to a thickened peritoneal membrane, which can encase and constrict the bowel. It can cause bowel obstruction and is associated with long-term peritoneal dialysis. Fungal peritonitis might present with ascites and possibly septations within the peritoneal cavity. However, a primary feature is the presence of fungi, which can sometimes be seen on imaging but are typically diagnosed through fluid analysis. Peritoneal carcinomatosis is the spread of malignancy within the peritoneal cavity. It can manifest as nodular thickening of the peritoneal lining and/or presence of ascitic fluid. While it might be considered given the bloody dialysate, other clinical findings might support this diagnosis more. Tuberculous peritonitis is a form of extrapulmonary tuberculosis. It might show thickened peritoneum, ascites, and sometimes omental caking, but the presentation is usually more subacute.	**Annotation:** Correct<br>**Comment:** None
**Step-by-step Reasoning:** Given the significant and irregular thickening of the peritoneal lining encasing the bowel, the most consistent diagnosis with the provided clinical history and the image is Encapsulating peritoneal sclerosis (EPS). The patient's long history of peritoneal dialysis and recurrent peritonitis episodes put her at a higher risk for EPS. While the other options can have some overlapping features, they are less consistent with the overall clinical and imaging presentation.	**Annotation:** Partially correct<br>**Comment:** The extensive calcifications are vital to achieving the correct anwer (encapsulating peritoneal sclerosis). GPT4 is able to come up with correct answer, but this is an important point that it missed.
**Final Answer:** Therefore, the most likely diagnosis is: B. Encapsulating peritoneal sclerosis.	**Annotation:** Correct

Q34. NEJM Image Challenge 2/16/23

An 80-year-old woman was admitted to the intensive care unit with severe second- and third-degree burns. On laboratory studies, she was noted to have a platelet count of 930,000 per cubic millimeter. Manual analysis of a subsequent peripheral-blood smear identified a platelet count of 115,000 per cubic millimeter. What is the cause for this discrepancy?

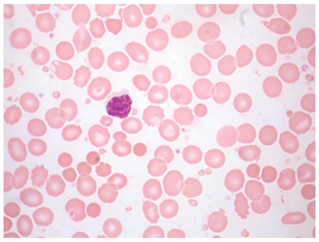

A. Pseudothrombocytopenia due to giant platelets
B. Pseudothrombocytopenia due to plate clumping associated with EDTA
C. Pseudothrombocytosis due to cryoglobulin-related interference by cryoprecipitates
D. Pseudothrombocytosis due to presence of microorganisms
E. Pseudothrombocytosis due to red cell fragments

Correct Answer: Pseudothrombocytosis due to red cell fragments
Explanation: The correct answer is pseudothrombocytosis due to red cell fragments. In patients with severe burn injuries, heat-induced disruption of the red-cell membranes can generate smaller cells that may be incorrectly counted as platelets by automated counters.

GPT-4V Answer

GPT-4V Answer	Annotation
**Image Comprhension:** The image provided shows a microscopic view of a blood smear. The most abundant cells are erythrocytes (red blood cells), which appear as pink, biconcave discs. There is also a notable presence of what appear to be large platelets or aggregated platelets among the erythrocytes. Additionally, there's a single larger cell with a multilobed nucleus, which is likely a neutrophil. The primary observation of interest is the large size or aggregation of the platelets, as this is relevant to the question posed.	**Annotation:** Incorrect<br>**Comment:** None
**Recall of Medical Knowledge:** Pseudothrombocytopenia refers to a falsely low platelet count. EDTA (ethylenediaminetetraacetic acid) is an anticoagulant commonly used in blood collection tubes. In some individuals, platelets can clump in the presence of EDTA, leading to falsely low platelet counts in automated counters but correct counts upon manual examination. Giant platelets can also cause pseudothrombocytopenia, as automated machines might mistake them for white blood cells, leading to an underestimation of the platelet count. Pseudothrombocytosis refers to a falsely elevated platelet count. This can occur due to interference from various factors such as cryoglobulins, microorganisms, or red cell fragments in automated counters. Cryoglobulins are proteins that precipitate at cold temperatures and can interfere with platelet counting. Microorganisms in the blood can be misinterpreted by automated machines as platelets, leading to pseudothrombocytosis. Red cell fragments, which are small pieces of broken red blood cells, can also be misinterpreted as platelets by automated machines.	**Annotation:** Correct<br>**Comment:** None
**Step-by-step Reasoning:** The given information mentions a significant discrepancy between the automated and manual platelet counts. The image shows large or clumped platelets, which are not typically seen in cases of interference from cryoglobulins, microorganisms, or red cell fragments. Given the presentation of large or clumped platelets in the image and the knowledge that EDTA can cause platelet clumping, option B (Pseudothrombocytopenia due to plate clumping associated with EDTA) seems the most likely explanation for the discrepancy between the automated and manual platelet counts.	**Annotation:** Incorrect<br>**Comment:** None
**Final Answer:** Answer: B. Pseudothrombocytopenia due to plate clumping associated with EDTA.	**Annotation:** Incorrect

Q35. NEJM Image Challenge 2/9/23

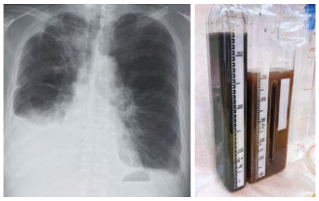

A 77-year-old man with metastatic lung adenocarcinoma presented to the emergency department with a 2-week history of dyspnea. Imaging studies showed a pleural effusion on the right side, previously known liver metastases and perihepatic fluid, and new intrahepatic dilatation of the biliary ducts. A chest tube was placed, and the color of the drained pleural fluid was olive brown. What additional pleural-fluid test is most likely to reveal the diagnosis?

A. Bacterial gram stain and culture
B. Bilirubin
C. Cytology
D. Fungal stain and culture
E. Triglycerides

Correct Answer: Bilirubin
Explanation: The correct answer is pleural-fluid bilirubin levels. Pleural-fluid studies showed a total bilirubin level of 8.2 mg per deciliter and a direct bilirubin level of 7.5 mg per deciliter. The pleural-fluid triglyceride level was normal, and cultures and cytologic studies were negative. A diagnosis of bilothorax was made. Bilothorax occurs when bile flows into the pleural space. In this case, the mechanism was thought to be diaphragmatic defects caused by hepatic metastases.

GPT-4V Answer

GPT-4V Answer	Annotation
**Image Comprhension:** The image contains two parts: An X-ray image of the chest, possibly an anteroposterior (AP) view, showing opacification of the right hemithorax which could be suggestive of a pleural effusion. There are also notable changes in the lung fields, which are consistent with the history of lung adenocarcinoma. A photograph of two clear containers holding fluid. The fluid in one container is dark and olive-brown in color, which is mentioned as the drained pleural fluid. The other container holds a lighter colored fluid, possibly for comparison.	**Annotation:** Partially correct<br>**Comment:** This statement is vague: There are also notable changes in the lung fields. Better would be to state the location of the changes. The comment about the other container is interesting. It’s hard to know why the two containers have fluid with different colors.
**Recall of Medical Knowledge:** Pleural effusion is an abnormal accumulation of fluid in the pleural space. It can be caused by various conditions, including malignancies, heart failure, pneumonia, and liver diseases. An olive-brown pleural fluid color can be suggestive of bilious pleural effusion, which occurs when bile enters the pleural space, commonly due to a fistulous connection between the biliary system and the pleural cavity. Bilirubin is a component of bile. An elevated bilirubin level in pleural fluid is diagnostic of bilious pleural effusion or cholethorax. The presence of cancer cells in pleural fluid (positive cytology) indicates malignant pleural effusion. Other tests like bacterial gram stain, fungal stain, and triglyceride levels can also be used to diagnose other causes of pleural effusion.	**Annotation:** Correct<br>**Comment:** None
**Step-by-step Reasoning:** Given the provided history and imaging findings, the patient’s presentation is consistent with a pleural effusion on the right side. The olive-brown color of the drained pleural fluid is unique and is suggestive of bilious pleural effusion or cholethorax. The presence of new intrahepatic dilatation of the biliary ducts in the context of known liver metastases and perihepatic fluid may suggest an obstruction or damage to the biliary system, leading to bile leakage into the pleural space. To confirm the diagnosis of bilious pleural effusion, the pleural fluid bilirubin level should be checked. If it’s elevated, it would confirm the presence of bile in the pleural fluid. Therefore, the most appropriate test to confirm the diagnosis in this case would be to check the bilirubin level in the pleural fluid.	**Annotation:** Correct<br>**Comment:** None
**Final Answer:** Answer: B.Bilirubin.	**Annotation:** Correct

Q36. NEJM Image Challenge 2/2/23

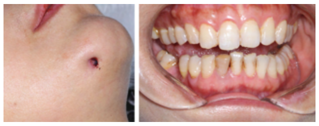

A previously healthy 42-year-old woman presented to the dental clinic with a 6-month history of swelling and pain on the right side of her chin (left). She reported no history of chin trauma, tooth pain, or fevers, but did recall injuring her right lateral incisor 10 years prior. Palpation of the lesion caused pain and serosanguinous fluid drainage. Intra-oral examination showed discoloration of the right lateral mandibular incisor (right). What is the most likely diagnosis?

A. Odontogenic cutaneous fistula
B. Osteosarcoma
C. Pilar cyst
D. Sebaceous cyst
E. Squamous cell carcinoma

Correct Answer: Odontogenic cutaneous fistula
Explanation: The answer is odontogenic cutaneous fistula. Odontogenic cutaneous fistula is caused by chronic infection of the tooth root due to dental caries, periodontal disease, or tooth fracture, as was likely to have occurred in this case. The condition manifests as dimpling, a nodule or cyst on the chin, jaw or elsewhere on the face. Misdiagnosis may occur due to the variable appearance and possible lack of dental symptoms.

GPT-4V Answer

**Image Comprhension:** The image on the left shows a close-up of a portion of an individual's face, specifically the chin area. There is an evident raised lesion on the chin with a small central opening that appears to have some drainage, consistent with the description of serosanguinous fluid drainage mentioned. The image on the right displays an intra-oral view, focusing on the lower teeth. One of the incisors, presumably the right lateral mandibular incisor as mentioned, is visibly discolored compared to the adjacent teeth.	**Annotation:** Correct<br>**Comment:** None
**Recall of Medical Knowledge:** Odontogenic cutaneous fistula: This is an abnormal pathway between the tooth root and the skin. It can be caused by dental abscesses that have eroded through the skin. Patients may present with a chronic draining lesion on the face, often misdiagnosed as a skin infection or cyst. Osteosarcoma: A malignant bone tumor. It would be rare for it to present as a lesion on the chin without other signs like bone destruction on imaging. Pilar cyst: A type of cyst that forms around hair follicles. These are typically not associated with dental issues. Sebaceous cyst: A cyst formed from blocked sebaceous glands. While they can appear on the face, they are not linked with dental problems. Squamous cell carcinoma: A type of skin cancer. It could present as a lesion on the skin, but the linkage to a dental issue a decade prior makes it less likely.	**Annotation:** Correct<br>**Comment:** None
**Step-by-step Reasoning:** Given the patient's presentation and the image provided:<br>The presence of a lesion on the chin, combined with a history of injuring a tooth and the intra-oral discoloration of the incisor, points towards a dental origin. The serosanguinous fluid drainage from the lesion on palpation indicates an active draining pathway. There's no mention of bone destruction, rapid growth, or other alarming signs that would point to a malignancy like osteosarcoma or squamous cell carcinoma. Pilar and sebaceous cysts would not be related to the dental injury and would not explain the intra-oral findings. Considering all the above, the most likely diagnosis for the patient's presentation is an odontogenic cutaneous fistula. The injury to the incisor could have led to a dental infection that eventually created a pathway (fistula) to the skin surface on the chin, leading to the observed clinical findings.	**Annotation:** Correct<br>**Comment:** None
**Final Answer:** Answer: A.Odontogenic cutaneous fistula.	**Annotation:** Correct

Q37. NEJM Image Challenge 1/26/23

A previously healthy 14-year-old boy who lived on a farm presented with a 1-month history of episodic headaches associated with vomiting. Magnetic resonance imaging of the head was performed. Which of the following is the most likely diagnosis?

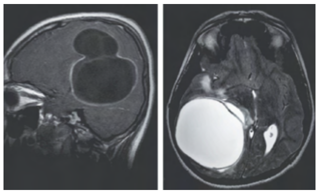

A. Arachnoid cysts
B. Colloid cyst
C. Cystic echinococcosis
D. Neurocysticercosis
E. Pineal cyst

Correct Answer: Cystic echinococcosis
Explanation: The correct answer is cystic echinococcosis. Magnetic resonance imaging of the head showed a multiloculated cyst in the right temporoparietal region of the brain with a hypointense rim and small projections in T2 phase. A craniotomy was performed to excise the cyst, and histopathology confirmed the diagnosis of Echinococcus granulosus. A 3-month course of albendazole was prescribed, and the patient's symptoms resolved.

GPT-4V Answer

**Image Comprhension:** The images provided are cross-sectional views of MRI scans of the head. The most prominent feature observed in both images is a large cystic lesion in the brain. The lesion appears to be multi-loculated, indicating that there are multiple compartments or chambers inside the cyst. It occupies a significant portion of the intracranial space, leading to mass effect with compression of surrounding brain structures.	**Annotation:** Partially correct<br>**Comment:** Cross sectional is not sufficient to describe the views. They are sagittal and axial views. The description of the multiloculated cyst is good. It did not describe the location of the cystic lesion, but that it has mass effect.
**Recall of Medical Knowledge:** A. Arachnoid cysts: These are cerebrospinal fluid-filled sacs that are located between the brain or spinal cord and the arachnoid membrane, one of the three membranes that cover the brain and spinal cord. They are often congenital and can be asymptomatic unless they exert mass effect on adjacent brain structures.<br>B. Colloid cyst: It is a benign brain cyst usually found in the anterior third ventricle. The presentation can be variable, including sudden death if it obstructs cerebrospinal fluid flow.<br>C. Cystic echinococcosis (CE): Caused by the larval stages of the tapeworm Echinococcus granulosus. Humans can become infected through ingestion of parasite eggs in contaminated food, water, or soil, or through direct contact with animal hosts. The most common sites of these cysts in humans are the liver and lungs, but they can also be found in the brain. Cystic echinococcosis is often associated with farming or living in rural areas close to livestock.<br>D. Neurocysticercosis: Caused by the larval stages of the tapeworm Taenia solium. Humans can become infected through ingestion of undercooked pork or contaminated water. It is the most common parasitic disease of the nervous system and is prevalent in many developing countries.<br>E. Pineal cyst: A cyst that forms in the pineal gland. It can be asymptomatic unless it grows in size, leading to compression of adjacent structures.	**Annotation:** Correct<br>**Comment:** None
**Step-by-step Reasoning:** Given the patient's history of living on a farm, there is an increased risk of exposure to certain parasitic infections like cystic echinococcosis (CE) and neurocysticercosis. However, other diagnoses should not be ruled out based solely on this information. The MRI shows a multi-loculated cystic lesion, which is a common feature of cystic echinococcosis (CE). While neurocysticercosis can present with cysts in the brain, they typically are not multi-loculated. Arachnoid cysts, colloid cysts, and pineal cysts can present as cystic lesions on MRI, but they don't typically show the multi-loculated appearance as seen in the provided image. Considering the presentation, MRI findings, and patient's history of living on a farm, the most likely diagnosis is cystic echinococcosis.	**Annotation:** Correct<br>**Comment:** None
**Final Answer:** Answer: C.Cystic echinococcosis	**Annotation:** Correct

Q38. NEJM Image Challenge 1/19/23

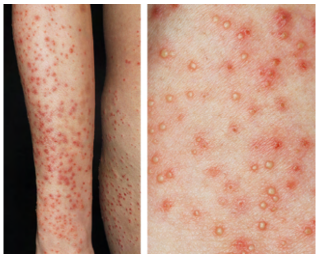

A 37-year-old primigravid woman presented at 30 weeks' gestation with a 4-month history of pruritic pustules on her torso, arms, and legs. On examination, there were papules, nodules, and follicular pustules with surrounding erythema, with no lesions on the palms and soles. A skin biopsy showed subepidermal pustules and perifollicular neutrophilic infiltration. Gram's stain, periodic acid–Schiff stain, and Grocott methenamine silver stain were all negative. What is the most likely diagnosis?

A. Atopic eruption of pregnancy
B. Disseminated herpes zoster
C. Pemphigoid gestationis
D. Polymorphic eruption of pregnancy
E. Secondary syphilis

Correct Answer: Atopic eruption of pregnancy
Explanation: A diagnosis of atopic eruption of pregnancy was made. Atopic eruption of pregnancy is the most common dermatosis of pregnancy. It includes eczema, prurigo, and as seen in this case, pruritic folliculitis. Owing to the patient's severe pruritus, cyclosporine and a high dose of oral glucocorticoid were prescribed. At 2 weeks of follow-up, the lesions had abated.

GPT-4V Answer

**Image Comprhension:** The image shows a close-up of skin, presumably from the described woman. On the skin are multiple erythematous (red) areas with numerous small, raised pustules. Some of the pustules seem to be centered around hair follicles, while others are more randomly dispersed. There is visible inflammation and the pustules vary in size, with some appearing to be slightly larger and more nodular.	**Annotation:** Correct<br>**Comment:** None
**Recall of Medical Knowledge:** Atopic eruption of pregnancy: This is a broad term that includes several pruritic conditions of pregnancy. However, the primary manifestations usually do not include pustules. Disseminated herpes zoster: This would typically show grouped vesicles on an erythematous base, often with a dermatomal distribution, and might be associated with pain. Pemphigoid gestationis: A rare autoimmune blistering disease that can occur during pregnancy. It often starts with pruritus, followed by the appearance of urticarial plaques and blisters. It might have a subepidermal split on histology. Polymorphic eruption of pregnancy: Previously known as pruritic urticarial papules and plaques of pregnancy (PUPPP), it is the most common pruritic condition of pregnancy. Lesions initially appear within stretch marks and may spread. Papules and plaques, not pustules, are the main lesions. Secondary syphilis: Can present with a variety of skin manifestations including maculopapular rash, which can involve the palms and soles, but pustules are not typical.	**Annotation:** Correct<br>**Comment:** None
**Step-by-step Reasoning:** The image does not show vesicles in a dermatomal pattern, ruling out herpes zoster. Atopic eruption usually doesn't manifest with pustules. The biopsy findings of subepidermal pustules align with pemphigoid gestationis. Also, pemphigoid gestationis starts with pruritus and can show urticarial plaques and blisters, somewhat similar to the image. Polymorphic eruption of pregnancy usually starts within stretch marks, which doesn't align with the described presentation. Secondary syphilis was ruled out because there are no lesions on the palms and soles, and pustules aren't typical for it.	**Annotation:** Incorrect<br>**Comment:** Pemphigoid gestationis usually present with vesicles on urticarial base and not folliculitis
**Final Answer:** Based on the image comprehension, recall of medical knowledge, and step-by-step reasoning, the most likely diagnosis is: C.Pemphigoid gestationis.	**Annotation:** Incorrect

Q39. NEJM Image Challenge 1/12/23

A previously healthy 5-year-old boy was brought to the surgery clinic with a 2-day history of intermittent abdominal pain. On palpation of the abdomen there was pain in the periumbilical region, but no rebound or guarding. An ultrasound was normal, and a computed tomography of the abdomen was performed (Panels A,B). Based on the CT findings, what is the most likely etiology of the patient's symptoms?

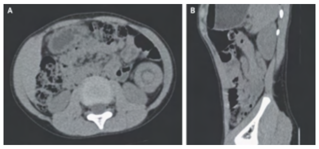

A. Crohn's disease
B. Colocolonic intussusception
C. Colonic pseudo-obstruction
D. Ileocolic intussusception
E. Sigmoid volvulus

Correct Answer: Colocolonic intussusception
Explanation: The correct answer is colocolonic intussusception. Panels A and B show a target sign and telescoping in the descending colon, respectively. Colonic intussusception is a rare cause of intestinal obstruction in children, and most cases are ileocolic rather than colocolonic. After a pneumatic reduction was performed in this case, the patient's symptoms resolved.

**GPT-4V Answer**	
**Image Comprhension:** The computed tomography (CT) images of the abdomen depict two views: Panel A shows a cross-sectional or axial view of the abdomen. In this image, there is an evident "target" or "doughnut" sign, which is represented by a circular configuration of different radiodensities. Specifically, there seems to be a central area of soft tissue density surrounded by a hypodense or fat-containing region, which is then encased by an outer hyper-dense ring.<br>Panel B shows a sagittal or side view of the abdomen. In this view, there's a continuation of the aforementioned target sign, suggesting the telescoping of one bowel segment into another.	**Annotation:** Partially correct<br>**Comment:** It is important to note that the target sign seems to be located in the descending colon rather than the small bowel or small bowel into large bowl (ilecolic).
**Recall of Medical Knowledge:** Intussusception is a condition where one segment of the bowel "telescopes" or slides into an adjacent section. This can cause bowel obstructions and reduce blood flow, leading to ischemia. The characteristic CT sign of intussusception is the "target" or "doughnut" sign.<br>Crohn's disease often presents with segmental bowel wall thickening, skipped lesions, and potentially fistula formations. The target sign isn't a characteristic feature.<br>Colonic pseudo-obstruction (also known as Ogilvie's syndrome) is a condition where there's a functional obstruction without a physical cause. CT would likely show a significant colonic distension.<br>Sigmoid volvulus occurs when the sigmoid colon twists around its mesentery. This can cause bowel obstructions, and the CT might show a "whirlpool" sign due to the twisted mesentery.	**Annotation:** Partially correct<br>**Comment:** Colonic pseudo-obstruction does show dilated colon but there are no transition points or organic causes. Intussusception can cause obstruction leading to colonic distention as well.
**Step-by-step Reasoning:** Crohn's disease (Option A): Although Crohn's can present with abdominal pain, the absence of segmental bowel wall thickening, skipped lesions, and the distinct target sign on the CT makes this less likely.<br>Colocolonic intussusception (Option B): This refers to the intussusception involving segments of the colon. The CT does show the target sign, but it's important to consider where the sign is located and the patient's age.<br>Colonic pseudo-obstruction (Option C): The CT does not show significant colonic distension, making this option unlikely.<br>Ileocolic intussusception (Option D): This refers to the intussusception where the ileum (part of the small intestine) telescopes into the colon. Given the patient's age (5 years old) and the location of the target sign around the periumbilical region, ileocolic intussusception is a common cause of abdominal pain in pediatric patients. The CT findings align with this diagnosis.<br>Sigmoid volvulus (Option E): The absence of a whirlpool sign and the presence of a target sign makes this diagnosis unlikely.	**Annotation:** Incorrect<br>**Comment:** The CT shows a descending colon location rather than a ileocolonic location of the 'target sign'. This is incorrect.
**Final Answer:** Based on the image comprehension and recall of medical knowledge, the most likely etiology of the patient's symptoms is D. Ileocolic intussusception.	**Annotation:** Incorrect

Q40. NEJM Image Challenge 1/5/23

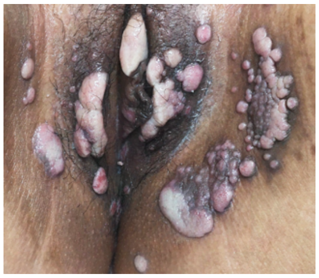

A 17-year-old girl presented with a 1-month history of pruritic genital lesions. Three months before presentation, she had unprotected sexual intercourse with multiple male partners. On exam, multiple smooth, grayish-white papules and plaques were noted on the vulva and upper inner thighs. The lesions had a smooth, moist appearance. What is the most likely diagnosis?

A. Condylomata acuminata
B. Condylomata lata
C. Extraintestinal Crohn's disease
D. Herpes vegetans
E. Mpox

Correct Answer: Condylomata lata
Explanation: A Treponema pallidum particle agglutination assay was positive, and subsequent testing revealed an elevated rapid plasma reagin titer of 1:16. Condylomata lata (highly infectious skin lesions) of secondary syphilis may be confused with condylomata acuminata (genital warts) but typically have a smoother, moist appearance. Treatment with intramuscular penicillin G benzathine was initiated. Testing for other sexually transmitted infections was negative. At the 1-month follow-up visit, the lesions had resolved, and the rapid plasma reagin titer was 1:8.

GPT-4V Answer

**Image Comprhension:** The image displays a close-up of the genital area, revealing multiple grayish-white papules and plaques. These lesions appear smooth and moist. They are spread across the vulva and seem to extend to the upper inner thighs.	**Annotation:** Correct<br>**Comment:** None
**Recall of Medical Knowledge:** Condylomata acuminata: These are genital warts caused by the human papillomavirus (HPV). They often appear as flesh-colored or gray swellings in the genital area. They can be raised or flat, single or multiple, small or large, and sometimes they join together to form shapes that resemble cauliflower.<br>Condylomata lata: These are manifestations of secondary syphilis. They present as large, raised, grayish-white lesions that are moist and may appear in the genital or anal areas.<br>Extraintestinal Crohn's disease: Crohn's disease is a type of inflammatory bowel disease that can affect any part of the gastrointestinal tract. When it affects areas outside the intestine, it's termed extraintestinal. Involvement of the skin, such as the genital area, can lead to skin lesions, but they typically don't look like the ones in the image.<br>Herpes vegetans: This is a rare presentation of herpes simplex virus (HSV). It's characterized by vegetating, foul-smelling, and persistent lesions, but they look different than the ones in the image.<br>Mpox: I'm not aware of a condition named "Mpox" that matches the context provided.	**Annotation:** Correct<br>**Comment:** None
**Step-by-step Reasoning:** Given the description and the image:<br>The lesions are not typical of the cauliflower-like appearance seen in condylomata acuminata. The lesions fit the description of condylomata lata in terms of their smooth, moist, and grayish-white appearance. The patient's history of unprotected intercourse with multiple partners also increases the risk of sexually transmitted infections, including syphilis which causes condylomata lata. The appearance of the lesions does not fit the characteristics of extraintestinal Crohn's disease or herpes vegetans. There is no recognized condition named "Mpox" that fits this context.	**Annotation:** Partially correct<br>**Comment:** ChatGPT was unable to recognize monkey pox
**Final Answer:** Considering the above information, the most likely diagnosis is B. Condylomata lata.	**Annotation:** Correct

Q41. NEJM Image Challenge 12/29/22

A 34-year-old man was admitted to the hospital with diabetic ketoacidosis. He reported having had blurry vision, increasing shoe size, and a change in his facial features over the past several years. On exam, he was found to have bitemporal hemianopsia. Which of the following is the most likely cause of his presentation?

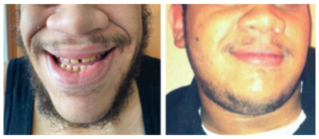

A. Anabolic steroid use
B. Beckwith–Wiedemann syndrome
C. Nelson's syndrome
D. Pachydermoperiostosis
E. Somatotropic pituitary adenoma

Correct Answer: Somatotropic pituitary adenoma
Explanation: The correct answer is somatotropic pituitary adenoma. Laboratory studies showed elevated levels of insulin-like growth factor 1 and growth hormone, confirming a diagnosis of acromegaly. Magnetic resonance imaging of the head revealed a pituitary macroadenoma, which was then resected and found on pathology to stain positive for growth hormone. He was treated with octreotide and required a second resection 8 months after the initial surgery.

GPT-4V Answer

**Image Comprhension:** The image displays two close-up partial facial photographs of an individual. The primary features visible include: Prominent and protruding lower jaw (mandibular prognathism). Thickened, coarse facial skin with enlarged pores. Enlarged and spaced-out teeth. Enlarged nose and lips. Sparse facial hair.	**Annotation:** Correct<br>**Comment:** None
**Recall of Medical Knowledge:** Diabetic ketoacidosis (DKA) is a serious complication of diabetes that occurs when the body produces high levels of blood acids called ketones. Bitemporal hemianopsia is a visual defect in which the patient has loss of peripheral vision in both eyes. This commonly arises due to compression of the optic chiasm. Enlarged shoe size, change in facial features, and the specific findings in the image are consistent with acromegaly, a condition caused by excessive growth hormone. Acromegaly is most commonly due to a benign pituitary tumor (adenoma) producing excessive growth hormone. This is referred to as a somatotropic pituitary adenoma. Other options mentioned: Anabolic steroid use: These can cause many side effects but not the changes mentioned. Beckwith–Wiedemann syndrome: A genetic disorder causing overgrowth and increased risk of tumors in childhood. Nelson's syndrome: Occurs after removal of both adrenal glands and is characterized by a rapid increase in size of a pre-existing pituitary corticotroph tumor. Pachydermoperiostosis: A rare genetic disorder leading to skin thickening, but it doesn't cause the other features mentioned.	**Annotation:** Correct<br>**Comment:** None
**Step-by-step Reasoning:** The individual in the image has facial features consistent with acromegaly, including mandibular prognathism, coarse facial skin, enlarged nose and lips, and spaced-out teeth. Acromegaly results from an excess of growth hormone, most commonly due to a pituitary adenoma. Bitemporal hemianopsia is indicative of compression of the optic chiasm, which is anatomically located near the pituitary gland. A large pituitary tumor pressing on the optic chiasm would cause this visual defect. Given the constellation of symptoms in the clinical vignette (blurry vision, increased shoe size, facial changes) combined with the bitemporal hemianopsia on exam and the image findings, the most likely cause is a somatotropic pituitary adenoma. The other options do not align with all the clinical and visual findings provided.	**Annotation:** Correct<br>**Comment:** None
**Final Answer:** E. Somatotropic pituitary adenoma.	**Annotation:** Correct

Q42. NEJM Image Challenge 12/22/22

An 18-year-old man presented with a 2-day history of fever, vomiting, and diarrhea. Three weeks before presentation, he had fallen into a canal. Physical exam was notable for the findings shown in the image. Laboratory studies showed acute kidney injury and elevations in aminotransferase and total bilirubin levels. What is the most likely diagnosis?

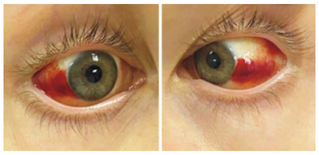

A. Acute hemorrhagic conjunctivitis
B. Ebola hemorrhagic fever
C. Giardiasis
D. Leptospirosis
E. Subarachnoid hemorrhage

Correct Answer: Leptospirosis
Explanation: Leptospirosis manifesting as fever, jaundice, renal failure, and hemorrhage was suspected on the basis of the patient's classic presentation (including the subconjunctival bleeding) and exposure to water with probable contamination by rodent urine. Empirical treatment with intravenous penicillin was initiated. Serum nucleic acid amplification testing identified leptospira species of the serogroup icterohaemorrhagiae. At follow-up 3 weeks later, his symptoms, subconjunctival hemorrhage, and liver and kidney injury had resolved.

GPT-4V Answer

**Image Comprhension:** The image displays two close-up views of a human eye. Both images appear to show the same eye with subtle differences in angle or lighting. The white part of the eye (sclera) is prominently reddened in its lower half. The redness seems to be due to blood, giving a hemorrhagic appearance. There's no obvious discharge, swelling, or other abnormalities visible in the eyelid or surrounding structures.	**Annotation:** Correct<br>**Comment:** None
**Recall of Medical Knowledge:** Acute hemorrhagic conjunctivitis: A viral infection that affects the eyes. Symptoms typically include sudden onset of pain, swelling, tearing, and redness in the eyes.<br>Ebola hemorrhagic fever: A viral disease characterized by fever, muscle pain, sore throat, and severe internal and external bleeding. It's a severe, often fatal illness and outbreaks occur primarily in Africa.<br>Giardiasis: An infection in your small intestine. It's caused by a microscopic parasite. While it can lead to symptoms such as diarrhea and abdominal pain, it does not typically cause eye symptoms.<br>Leptospirosis: A bacterial disease that can lead to a wide range of symptoms. Some infected persons may have no symptoms at all. Others might have symptoms that can range from mild to severe. It can be caught from water contaminated by infected animal urine. Symptoms can include high fever, severe headache, muscle pain, chills, redness of the eyes (conjunctival suffusion), abdominal pain, jaundice (yellow skin and eyes), hemorrhages in the skin and mucous membranes, vomiting, diarrhea, and rash.<br>Subarachnoid hemorrhage: Bleeding into the space between the brain and the tissue covering the brain, typically caused by a ruptured aneurysm. Symptoms can include a sudden, severe headache, often described as the "worst headache" one has ever had.	**Annotation:** Correct<br>**Comment:** None
**Step-by-step Reasoning:** Given the provided history and the findings in the image:<br>The patient's symptoms started after falling into a canal, suggesting exposure to possibly contaminated water. The most relevant symptoms in relation to the options provided are fever, vomiting, diarrhea, kidney injury, liver abnormalities, and the hemorrhagic conjunctiva. Among the options, Giardiasis, while associated with contaminated water, does not typically present with the eye or systemic findings described. Acute hemorrhagic conjunctivitis is primarily an eye condition and would not explain the systemic findings. Ebola hemorrhagic fever can cause severe systemic symptoms and hemorrhagic findings, but there's no mention of the patient being in an area endemic for Ebola or having exposure to someone with the disease. Subarachnoid hemorrhage primarily presents with neurological symptoms, which are not described here. Leptospirosis fits the clinical picture best. The exposure to potentially contaminated water, systemic symptoms, kidney and liver involvement, and the hemorrhagic conjunctiva are all consistent with leptospirosis.	**Annotation:** Correct<br>**Comment:** None
**Final Answer:** Based on the above reasoning, the most likely diagnosis is: D. Leptospirosis.	**Annotation:** Correct

Q43. NEJM Image Challenge 12/15/22

A 58-year-old woman with a history of metastatic uterine leiomyosarcoma presented with a 1-month history of a nonpruritic rash on her arms. On examination, hyperpigmented plaques were observed on the dorsa of both hands at the sites of previous intravenous access. The darkened skin extended up the arms in a linear pattern along the network of superficial veins; the lesions were palpable but non-tender. What is the most likely diagnosis?

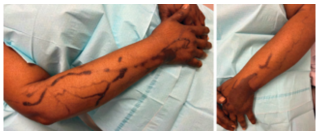

A. Bleomycin-induced flagellate erythema
B. Erythema ab igne
C. Phlegmasia cerulea dolens
D. Serpentine supravenous hyperpigmentation
E. Superficial thrombophlebitis

Correct Answer: Serpentine supravenous hyperpigmentation
Explanation: A diagnosis of docetaxel-associated serpentine supravenous hyperpigmentation was made. The mechanism of skin hyperpigmentation remains unclear, but the reaction is benign; the underlying veins remain patent. A central venous catheter was placed for subsequent administration of chemotherapy. At a follow-up visit 2 months later, the rash had abated.

GPT-4V Answer

**Image Comprhension:** The image presents an arm and hand showing hyperpigmented streaks and patches. These hyperpigmented areas follow the superficial veins' pattern on the arm, particularly evident on the dorsum of the hand and extending up the forearm and possibly the arm. The markings are in linear patterns.	**Annotation:** Correct<br>**Comment:** None
**Recall of Medical Knowledge:** Bleomycin-induced flagellate erythema: This is a characteristic side effect of the chemotherapy drug bleomycin. It presents as linear streaks on the body resembling whip marks, commonly on the trunk. Erythema ab igne: A condition caused by repeated exposure to moderate levels of infrared radiation or heat. It presents as a mottled, reddish-brown reticulated pattern on the skin. Phlegmasia cerulea dolens: A severe form of deep venous thrombosis which results in near-total venous occlusion. Symptoms include severe pain and swelling, with the affected limb turning bluish. Serpentine supravenous hyperpigmentation: Occurs after certain intravenous (IV) drug administrations. The pigmentation follows the path of the superficial veins where the drug was administered. Superficial thrombophlebitis: Inflammation of the superficial veins which can be caused by various factors including trauma. It presents as red, tender, warm linear areas overlying superficial veins.	**Annotation:** Correct<br>**Comment:** None
**Step-by-step Reasoning:** Given that the hyperpigmentation is noted on the dorsa of both hands at the sites of previous intravenous access and extends linearly along the network of superficial veins, this strongly suggests a drug reaction. Bleomycin-induced flagellate erythema often presents on the trunk and not specifically along veins. Erythema ab igne is related to heat exposure and would not specifically follow the venous pattern. Phlegmasia cerulea dolens is related to deep venous thrombosis and would not present solely as skin hyperpigmentation. Superficial thrombophlebitis would typically present as red, tender areas and not just as hyperpigmentation. Serpentine supravenous hyperpigmentation matches the presentation, where there is hyperpigmentation along the superficial veins after IV drug administration.	**Annotation:** Correct<br>**Comment:** None
**Final Answer:** Conclusion: The most likely diagnosis is D. Serpentine supravenous hyperpigmentation.	**Annotation:** Correct

Q44. NEJM Image Challenge 12/8/22

An 81-year-old woman with a history of hepatitis C virus–related cirrhosis and hepatocellular carcinoma, who was treated 9 days earlier with transarterial chemoembolization with the use of doxorubicin-eluting beads, presented with painful, progressively worsening skin lesions over the abdomen. A skin biopsy revealed epidermal necrosis and occlusion of small vessels in the reticular dermis. What is the most likely cause of the epidermal necrosis?

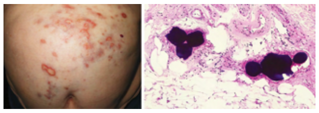

A. Air Embolism
B. Bacterial Embolism
C. Drug-Eluting Bead Embolism
D. Cholesterol Embolism
E. Tumor Embolism

Correct Answer: Drug-Eluting Bead Embolism
Explanation: The correct answer is embolization of the doxorubicin-eluting beads used in the procedure. The spread of chemoembolization material beyond the hepatic-artery vascular territory, or nontarget embolization, is a potential complication of the procedure. The patient was treated with oral pentoxifylline and topical glucocorticoids with improvement of the lesions but residual scarring observed at 6 months.

GPT-4V Answer

**Image Comprhension:** The image on the left side shows multiple red, irregularly shaped lesions on the skin, with varying sizes and some areas of central necrosis or ulceration. The right side of the image presents a histological view of skin tissue stained with hematoxylin and eosin. The epidermal layer shows areas of necrosis. Additionally, within the dermis, there are dark, amorphous materials occluding small vessels.	**Annotation:** Correct<br>**Comment:** None
**Recall of Medical Knowledge:** Air embolism typically results from the introduction of air into the bloodstream, which can cause ischemia in distant organs. Clinical presentations depend on the size and site of the embolism but skin manifestations are not typically seen. Bacterial embolism usually results from septicemia and can cause a variety of symptoms based on the site of the embolism. Septic emboli to the skin can cause painful nodules, abscesses, or ulcers. Drug-eluting bead embolism refers to the occlusion of small vessels by beads that are loaded with a chemotherapeutic agent, commonly used in procedures like transarterial chemoembolization for liver cancers. These beads can sometimes inadvertently travel to other sites, causing tissue damage. Cholesterol embolism or atheroembolism can occur when cholesterol crystals from plaques in large arteries get dislodged and travel to small vessels, leading to occlusion. Skin manifestations include livedo reticularis, blue toe syndrome, and ulcers. Tumor embolism is the occlusion of vessels by tumor cells. This is relatively rare and can lead to symptoms based on the site of embolism.	**Annotation:** Correct<br>**Comment:** None
**Step-by-step Reasoning:** Given that the patient was treated with transarterial chemoembolization with doxorubicin-eluting beads, there is a direct clue pointing towards the possibility of bead embolism. The histological image shows occlusion of small vessels in the dermis with dark amorphous materials, which aligns with the presentation of drug-eluting beads. Although bacterial embolism can cause skin lesions, the clinical history and histological findings don't strongly support this. The same applies to air, cholesterol, and tumor embolisms. The clinical history, skin presentation, and histology align most closely with drug-eluting bead embolism.	**Annotation:** Correct<br>**Comment:** None
**Final Answer:** Based on the above reasoning, the most likely cause of the epidermal necrosis is: C.Drug-Eluting Bead Embolism.	**Annotation:** Correct

Q45. NEJM Image Challenge 12/1/22

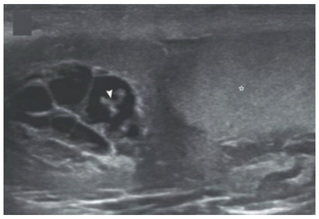

A 26-year-old man presented to the outpatient clinic with a 1-month history of pain and swelling in the scrotum and low-grade fevers. On examination, there was swelling and tenderness of the right side of the scrotum. Laboratory studies showed peripheral eosinophilia. An ultrasound of the scrotum showed echogenic, linear structures moving within the lymphatic channels (arrowhead) adjacent to the epididymal head and testis (asterisk) — a finding known as "filarial dance sign." What vector is responsible for transmitting the nematode causing this disease?

A. Aquatic snail
B. Blackfly
C. Mosquito
D. Sandfly
E. Tsetse fly

Correct Answer: Mosquito
Explanation: The correct answer is mosquito. The image shows scrotal lymphatic filariasis, a parasitic infection caused by mosquito-transmitted nematodes. The mobile echogenic structures represent the live worms that had migrated into the lymphatic channels. The diagnosis was confirmed when light microscopy of the scrotal aspirate identified Wuchereria bancrofti. After a 3-week course of oral diethylcarbamazine, the patient's symptoms abated, and a second ultrasound showed no evidence of the motile microfilariae.

GPT-4V Answer

Image Comprhension: The grayscale ultrasound image of the scrotum depicts linear echogenic structures located near the epididymal head and testis. The structures are highlighted with an arrowhead and are described as moving within the lymphatic channels. This specific appearance is referred to as the "filarial dance sign." Additionally, there's an asterisk marking what seems to be the epididymal head and testis.

Annotation: Correct
Comment: None

Recall of Medical Knowledge: Filarial worms are a group of parasitic nematodes that cause various forms of filariasis in humans. The "filarial dance sign" is a characteristic ultrasound finding seen in cases of lymphatic filariasis, which is caused by nematodes such as Wuchereria bancrofti, Brugia malayi, and Brugia timori. These nematodes are transmitted to humans through the bite of infected mosquitoes, predominantly from the genus Culex, Anopheles, and Aedes.

Annotation: Correct
Comment: None

Step-by-step Reasoning: The description and ultrasound image show the "filarial dance sign," which is characteristic of lymphatic filariasis. Lymphatic filariasis is caused by filarial worms like Wuchereria bancrofti, Brugia malayi, and Brugia timori. These worms are transmitted to humans via the bite of infected mosquitoes. Among the given choices, the vector responsible for transmitting these nematodes is the mosquito.

Annotation: Correct
Comment: None

Final Answer: Answer: C.Mosquito.

Annotation: Correct

Q46. NEJM Image Challenge 11/24/22

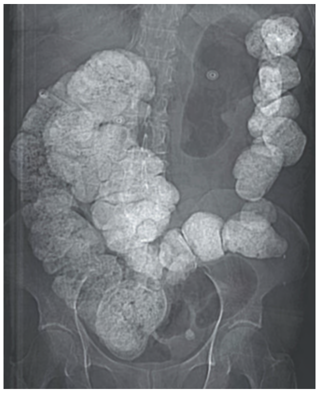

A 70-year-old woman with depression and irritable bowel syndrome presented to the emergency department with a 3-day history of altered mental status, shortness of breath, nausea, and vomiting. She had recently been self-medicating worsening irritable bowel symptoms. Physical examination was notable for a respiratory rate of 22 breaths per minute, confusion, and mild, diffuse abdominal pain. Laboratory testing showed primary anion-gap metabolic acidosis and primary respiratory alkalosis. Toxicity of which of the following is the most likely cause of her acute symptoms and the findings seen in this non-contrast abdominal radiograph?

A. Bismuth Salicylate
B. Calcium Carbonate
C. Ferrous Sulfate
D. Magnesium hydroxide
E. Simethicone

Correct Answer: Bismuth Salicylate
Explanation: The correct answer is bismuth salicylate. A salicylate level was found to be elevated to 76 mg per deciliter (5.50 mmol per liter; normal value, <5 mg per deciliter [0.35 mmol per liter]) and a diagnosis of bismuth salicylate toxicity was made. Large quantities of bismuth appear radiopaque owing to the high density of the substance, which approaches that of lead. After treatment with sodium bicarbonate infusion and hemodialysis for salicylate poisoning, the patient's symptoms abated.

GPT-4V Answer

**Image Comprhension:** The image appears to be a non-contrast abdominal radiograph. It displays multiple, well-defined, radio-opaque densities scattered throughout the large bowel and in the area corresponding to the stomach. These dense areas can be indicative of the ingestion of certain substances or medications that appear radiopaque on X-ray.	**Annotation:** Partially correct<br>**Comment:** The X-ray findings show more of a diffuse opacity than 'well-defined' densities.
**Recall of Medical Knowledge:** Bismuth Salicylate - Commonly found in over-the-counter anti-diarrheal medications, bismuth subsalicylate can cause a characteristic radiopacity in the bowel if taken in large amounts. Toxicity from bismuth salicylate can lead to salicylate poisoning, which is associated with symptoms such as altered mental status, tachypnea (leading to respiratory alkalosis), and metabolic acidosis. Calcium Carbonate - An antacid. Ingestion can result in hypercalcemia but doesn't usually cause the depicted radiological findings. Ferrous Sulfate - An iron supplement. Overdose can cause gastrointestinal symptoms, but it does not produce the radiological findings shown in the image. Magnesium hydroxide - Used as a laxative and antacid. Excessive intake can result in hypermagnesemia with symptoms like muscle weakness, but it doesn't match the image findings. Simethicone - An anti-flatulent. It does not cause the depicted radiological findings.	**Annotation:** Correct<br>**Comment:** None
**Step-by-step Reasoning:** Considering the image and the clinical presentation:<br>The radiograph shows radio-opaque densities in the stomach and large bowel. The patient's clinical presentation, including altered mental status, primary anion-gap metabolic acidosis, and primary respiratory alkalosis, points towards a toxic ingestion that affects both metabolic and respiratory systems. From the provided options, bismuth salicylate toxicity matches both the clinical presentation (due to salicylate poisoning) and the radiological findings.	**Annotation:** Correct<br>**Comment:** None
**Final Answer:** Based on the above reasoning, the most likely cause of her acute symptoms and the findings seen in this non-contrast abdominal radiograph is A.Bismuth Salicylate.	**Annotation:** Correct

Q47. NEJM Image Challenge 11/17/22

A 45-year-old woman presented with a 6-week history of a painless upper-lip lesion. The lesion had rapidly increased in size over the previous 3 weeks and bled when lightly touched. Examination showed an erythematous, round, smooth, pedunculated mass that was friable and non-tender. What is the most likely diagnosis?

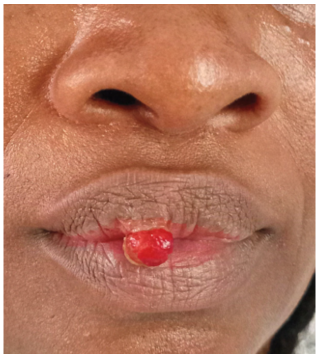

A. Arteriovenous malformation
B. Cherry hemangioma
C. Malignant melanoma
D. Pyogenic granuloma
E. Strawberry hemangioma

Correct Answer: Pyogenic granuloma
Explanation: The lesion was excised and histopathological examination showed proliferating vessels, fibroblasts, numerous inflammatory cells, and surface ulceration, findings consistent with pyogenic granuloma (also known as a lobular capillary hemangioma). At a 4-month follow-up visit, the lesion had not recurred.

GPT-4V Answer

**Image Comprhension:** The image depicts a close-up view of a person's upper lip area. The skin appears of a darker complexion, and the main feature of interest is a prominent red lesion on the upper lip. This lesion is erythematous (red in color) and has a pedunculated (stalk-like) appearance. The lesion looks smooth and round. No overt signs of necrosis, ulceration, or pigmentation are seen within this image.	**Annotation:** Correct<br>**Comment:** None
**Recall of Medical Knowledge:** Arteriovenous malformation (AVM): AVMs are tangled collections of blood vessels that can form anywhere in the body but most commonly in the brain or spine. They typically do not present as superficial skin lesions.<br>Cherry hemangioma: These are bright red, round or oval in shape, and small—usually ranging in size from a pinpoint to about one-fourth of an inch across. They can develop anywhere on the body, but most commonly appear on the trunk.<br>Malignant melanoma: This is a type of skin cancer that usually appears as a dark, irregular mole. It's often associated with an asymmetrical appearance, irregular borders, varied color, and increasing size.<br>Pyogenic granuloma: Also known as lobular capillary hemangioma, this is a benign vascular tumor that appears as a small, reddish-brown bump, often growing rapidly over a period of a few weeks to months. They are friable, meaning they can bleed easily.<br>Strawberry hemangioma: This is a type of infantile hemangioma that appears at birth or in the first or second week of life. It looks like a rubbery bump and is made up of extra blood vessels in the skin.	**Annotation:** Correct<br>**Comment:** None
**Step-by-step Reasoning:** Based on the description provided and the image comprehension:<br>The lesion is not described as dark or mole-like, which makes malignant melanoma less likely. The presentation doesn't fit the typical appearance or age of presentation for a strawberry hemangioma. Cherry hemangiomas are small and do not typically present as a rapidly growing pedunculated mass. AVMs are not typically superficial skin lesions. The most fitting description is that of a pyogenic granuloma. These lesions can grow rapidly and are known to bleed easily when touched. The presentation of a painless, rapidly growing, pedunculated, and friable lesion on the lip is most consistent with a pyogenic granuloma.	**Annotation:** Correct<br>**Comment:** None
**Final Answer:** Answer: D.Pyogenic granuloma.	**Annotation:** Correct

Q48. NEJM Image Challenge 11/10/22

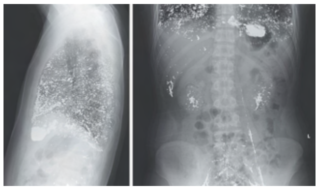

A previously healthy 25-year-old man presented to the general medicine clinic with a 3-month history of worsening fatigue, nausea, and dry cough following administration of a folk remedy for tinea cruris. Laboratory studies showed a hemoglobin level of 6.5 g per deciliter (reference range, 12.0 to 16.0), and normal renal function. Chest and abdominal radiographs were obtained. Which of the following is the appropriate treatment for this condition?

A. Chelation Therapy
B. Exchange Transfusion
C. Hemodialysis
D. Plasma Exchange Therapy
E. Therapeutic Phlebotomy

Correct Answer: Chelation Therapy
Explanation: The correct answer is chelation therapy. Further laboratory evaluation revealed a serum mercury level of 430 g per deciliter (21,440 nmol per liter; reference value, <1.5 g per deciliter [70 nmol per liter]) and a 24-hour urine mercury level of 29,792 g (148,510 nmol; reference value, <45 g [220 nmol]). A diagnosis of mercury poisoning was made. Inpatient chelation therapy was initiated. After 2 weeks of treatment, the patient's symptoms had abated and he was discharged to continue oral chelation therapy at home.

GPT-4V Answer	
**Image Comprhension:** The images provided appear to be radiographs of the chest and abdomen. There are multiple opacities scattered throughout the lung fields, which are suggestive of diffuse pulmonary infiltration. The abdominal radiograph shows dense radiopacities in a distribution consistent with ingestion and deposition within the gastrointestinal tract.	**Annotation:** Partially correct<br>**Comment:** Error: there appers to be diffuse deposition not just in the GI tract, including kidney, lung
**Recall of Medical Knowledge:** The clinical history and laboratory findings hint towards a case of poisoning or intoxication. The folk remedy for tinea cruris might have contained toxic metal or substances. Diffuse pulmonary infiltration can be seen in several conditions, but in the context of a possible poisoning, heavy metal intoxication such as lead poisoning is a strong consideration. Lead poisoning can manifest with gastrointestinal symptoms, anemia (low hemoglobin level), and diffuse pulmonary infiltration. Additionally, lead can be ingested and show up as radiopacities in the gastrointestinal tract. Treatment of lead poisoning includes removing the source of exposure and administering a chelating agent to enhance the excretion of the metal from the body.	**Annotation:** Partially correct<br>**Comment:** Error: the explanation focuses on lead, when the quesiton is about mercury, even though the treatment is similar. treatment of any metal poisoning typically start with chelation therapy. Most common type of metal poison is lead, mercury, arsenic.
**Step-by-step Reasoning:** The patient has been exposed to a potential toxic agent (folk remedy for tinea cruris). He has clinical manifestations consistent with heavy metal toxicity: fatigue, gastrointestinal symptoms, and anemia. The radiographs show findings suggestive of lead ingestion with its deposition in the gastrointestinal tract and also pulmonary manifestations. The most common treatment for significant lead poisoning is chelation therapy.	**Annotation:** Partially correct<br>**Comment:** Error: the question focuses on lead, and the question is about mercury. However there are not enough hints in the question to suggest that mercury is the cause.
**Final Answer:** Based on the above reasoning, the appropriate treatment for this condition is: A. Chelation Therapy.	**Annotation:** Correct

Q49. NEJM Image Challenge 11/3/22

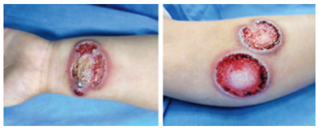

A 5-year-old boy from China with a history of IgA vasculitis at 2 years of age presented with a 1-month history of progressively painful skin ulcerations on his extremities. Two months prior, he had had an episode of nasal pustules that had not responded to antimicrobial therapy or surgical débridement, but had ultimately improved with 2 days of systemic glucocorticoid therapy. At the current presentation, laboratory and radiographic studies showed no signs of underlying systemic disease, such as cancer, inflammatory bowel disease, or autoimmune conditions. Biopsy samples from the lesions showed a dense neutrophilic infiltrate with negative tissue cultures. What is the diagnosis?

A. Cutaneous leishmaniasis
B. Cutaneous polyarteritis nodosa
C. Cutaneous tuberculosis
D. Ecthyma gangrenosum
E. Pyoderma gangrenosum

Correct Answer: Pyoderma gangrenosum
Explanation: The correct answer is pyoderma gangrenosum. These lesions have a classic appearance of sharply demarcated skin ulcerations with an erythematous base and purple border. Following 8 months of treatment with tapering doses of glucocorticoids, the lesions resolved completely.

GPT-4V Answer

**Image Comprhension:** The images depict two different views of a child's extremities. Both images show skin ulcerations with notable differences:<br>The first image on the left side shows a large oval-shaped ulceration on what appears to be the wrist region. The ulcer displays a deep red center with areas of yellowish crusting and is surrounded by an erythematous (reddened) border. The second image on the right side showcases two circular ulcerations located on the forearm. These ulcers are notably deep, showing a bright red center with a white border that seems to have a necrotic or crusty appearance.	**Annotation:** Correct<br>**Comment:** None
**Recall of Medical Knowledge:** Cutaneous leishmaniasis: This is caused by a protozoan parasite transmitted by the bite of sandflies. Lesions are typically nodular and can ulcerate, but they often have a raised border. Cutaneous polyarteritis nodosa (cPAN): A type of vasculitis affecting small and medium-sized arteries in the skin. It can lead to tender nodules, livedo reticularis, and ulcerations. Cutaneous tuberculosis: Caused by Mycobacterium tuberculosis, it leads to various skin manifestations, but the most common is lupus vulgaris which presents as reddish-brown plaques. Ecthyma gangrenosum: Typically associated with Pseudomonas aeruginosa bacteremia. It starts as a small erythematous macule that progresses to a necrotic ulcer with a surrounding erythematous halo. Pyoderma gangrenosum (PG): A rare inflammatory skin condition characterized by painful ulcers, typically with an irregular, violaceous, undermined border. Often associated with systemic diseases but can also appear idiopathically.	**Annotation:** Correct<br>**Comment:** None
**Step-by-step Reasoning:** The presence of nasal pustules in the patient's history, not responsive to antimicrobials or surgical intervention, but improving with glucocorticoids, suggests an inflammatory rather than infectious etiology. The biopsy showing a "dense neutrophilic infiltrate" with negative tissue cultures also leans towards an inflammatory condition. Cutaneous leishmaniasis and cutaneous tuberculosis are infectious diseases and are less likely given the biopsy findings and history. Ecthyma gangrenosum is primarily associated with Pseudomonas infection, and the biopsy did not show any signs of this infection. Cutaneous polyarteritis nodosa presents with nodules and livedo, which are not described here. Pyoderma gangrenosum, on the other hand, is an inflammatory skin condition with lesions that are painful and can start as pustules, which aligns with the patient's history. The lesions of PG can quickly progress to ulcers with an undermined border, which seems consistent with the images provided.	**Annotation:** Correct<br>**Comment:** None
**Final Answer:** Given the above, the diagnosis most consistent with the clinical presentation, biopsy findings, and the images provided is: E. Pyoderma gangrenosum.	**Annotation:** Correct

Q50. NEJM Image Challenge 10/27/22

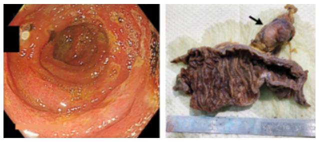

A 75-year-old man presented to the emergency department with a 6-month history of epigastric pain, watery diarrhea, and weight loss. Esophagogastroduodenoscopy revealed severe esophagitis, antral erosions, and duodenal ulcers (left). The gastric pH was below 2, and a fasting serum gastrin level was above 1000 pg per milliliter (reference range, 13-115). Cross-sectional imaging identified a single tumor along the distal duodenum that was resected (right). Histopathological analysis showed a well-differentiated neuroendocrine tumor that was positive for gastrin on immunohistochemical staining. The diagnosis in this case is most closely associated with which of the following syndromes?

A. Li–Fraumeni syndrome
B. Multiple endocrine neoplasia type
C. Multiple endocrine neoplasia type 2A
D. Multiple endocrine neoplasia type 2B
E. Von Hippel–Lindau syndrome

Correct Answer: Multiple endocrine neoplasia type 1
Explanation: The correct answer is multiple endocrine neoplasia type 1. The patient was diagnosed with Zollinger–Ellison syndrome, a syndrome of gastric acid hypersecretion resulting from a gastrin-producing neuroendocrine tumor. Multiple endocrine neoplasia type 1 — which this particular patient did not have — is characterized by pituitary adenomas, parathyroid hyperplasia and gastro-entero-pancreatic tract tumors.

GPT-4V Answer

Image Comprhension: The image is split into two sections. On the left side, it shows a close-up endoscopic view of a digestive tract, revealing a reddened and inflamed mucosal lining with multiple erosions. The right side of the image displays a resected tissue on a surface with a measuring ruler. The tissue appears to be from the digestive system, possibly the duodenum or a part of the stomach. It shows a dark, wrinkled appearance, and there's an arrow pointing to a particular raised portion or tumor on the tissue, suggesting it might be the site of interest or abnormality.

Annotation: Correct
Comment: None

Recall of Medical Knowledge: Multiple endocrine neoplasia (MEN) refers to a group of inherited disorders that can affect multiple endocrine organs, such as the thyroid, parathyroid, adrenal glands, and the neuroendocrine tissue of the gastrointestinal tract.
MEN type 1 (MEN1): Characterized by tumors of the parathyroid glands, the pancreas, and the pituitary gland. Gastrinomas (tumors that secrete gastrin, leading to the condition known as Zollinger-Ellison syndrome) are associated with MEN1. These tumors increase the secretion of gastric acid, which can result in peptic ulcers. MEN type 2A (MEN2A): This is characterized by medullary thyroid cancer, pheochromocytoma (tumors of the adrenal glands), and parathyroid tumors. MEN type 2B (MEN2B): Includes medullary thyroid cancer, pheochromocytoma, and characteristic physical features (like mucosal neuromas). It does not typically involve gastrointestinal neuroendocrine tumors. Li–Fraumeni syndrome: It is a rare hereditary disorder that increases the risk of developing several types of cancer, particularly in younger individuals. Von Hippel–Lindau syndrome: An inherited disorder characterized by the formation of tumors and cysts in many parts of the body, including the central nervous system.

Annotation: Correct
Comment: None

Step-by-step Reasoning: The presentation of severe esophagitis, antral erosions, duodenal ulcers, and a high fasting serum gastrin level suggests Zollinger-Ellison syndrome, which involves gastrin-secreting tumors (gastrinomas) that cause increased gastric acid production. Zollinger-Ellison syndrome is specifically associated with gastrin-secreting tumors of the duodenum or pancreas. Among the choices provided, only MEN1 is associated with gastrinomas and the development of Zollinger-Ellison syndrome. Thus, based on the presentation, imaging, and the associated syndrome with gastrinomas, the most closely associated syndrome in this case is MEN1.

Annotation: Correct
Comment: None

Final Answer: Answer: B. Multiple endocrine neoplasia type 1 (MEN1).

Annotation: Correct

Q51. NEJM Image Challenge 10/20/22

A 37-year-old man presented to the emergency department with a 1-week history of pain and swelling in the left upper arm that had started after blunt trauma during soccer practice. The heart rate was 120 beats per minute, the blood pressure 96/54 mm Hg, and the body temperature 37.9℃. Examination of the left upper arm was notable for swelling, tenderness, and crepitus. The overlying skin was red and warm to the touch. An initial radiograph is shown. Which of the following factors is most associated with increased mortality in this diagnosis?

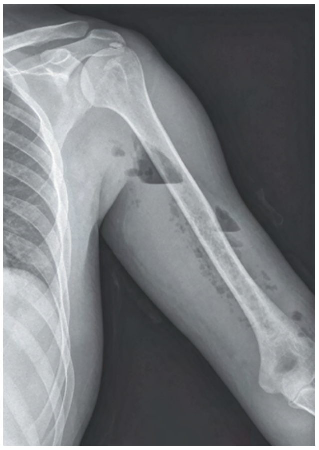

A. Delay in surgical intervention >24 hours
B. Involvement of the upper extremity
C. Negative Blood Cultures
D. Patient age <60 years
E. Presence of erythema

Correct Answer: Delay in surgical intervention >24 hours
Explanation: The correct answer is delay in surgical intervention >24 hours. The radiograph of the upper arm showed radiolucent areas with air–fluid levels in deep tissue, and subsequent computed tomography revealed pockets of gas in the muscles of the extensor compartment, raising concern about necrotizing myositis. This patient was taken to the OR for emergency surgical débridement, and the diagnosis was confirmed. After 3 weeks of broad-spectrum antimicrobial therapy, the patient regained function of his arm.

**GPT-4V Answer**	
**Image Comprhension:** The image depicts an X-ray radiograph of the upper left arm. Notably, there are areas of discontinuous bone (suggesting potential fractures), as well as signs of subcutaneous gas collection. The gas is indicated by the presence of dark, irregular shapes distributed within the tissues surrounding the bone. This is not a normal finding on a radiograph of an arm.	**Annotation:** Partially correct<br>**Comment:** The bone itself is not discontinuous; it is the subcutaneous gas that makes it look like so. There are, however, potentially some periosteum thickening.
**Recall of Medical Knowledge:** The clinical presentation of pain, swelling, warmth, redness, and crepitus, in combination with the observed subcutaneous gas on radiograph, is consistent with a diagnosis of necrotizing fasciitis (NF). NF is a severe bacterial infection that affects the fascia and can lead to the rapid destruction of muscle and other soft tissues. The presence of gas in the tissue suggests that the infection is caused by gas-producing bacteria, such as Clostridium perfringens or certain strains of Group A Streptococcus.<br>Key points about necrotizing fasciitis:<br>It is a rapidly progressing infection that requires urgent surgical intervention to remove the necrotic tissue. Delay in treatment is associated with a high rate of morbidity and mortality. The presence of systemic signs of infection, such as tachycardia and fever, suggest that the infection has become severe and is affecting the whole body. Mortality rates for necrotizing fasciitis can be high, especially when diagnosis and treatment are delayed.	**Annotation:** Correct<br>**Comment:** None
**Step-by-step Reasoning:** Based on the presentation and the image, the diagnosis of necrotizing fasciitis (NF) is highly suggestive. Of the options provided, delay in surgical intervention is a well-known factor that significantly impacts the prognosis of NF. Rapid surgical debridement is crucial in the management of this condition. Erythema, which is redness of the skin, is a sign of inflammation, but it is not necessarily a predictor of mortality in this context. Many patients with NF will present with erythema. The involvement of the upper extremity in itself is not the most significant factor for mortality in NF. Negative blood cultures may indicate that the bacteria haven't spread into the bloodstream or that the bacteria present aren't easily cultured. However, it is not the most significant factor for mortality when compared to delay in treatment. Age less than 60 years is generally associated with better outcomes in many conditions, but in the context of NF, the delay in treatment is a much more significant factor in determining outcomes.	**Annotation:** Correct<br>**Comment:** None
**Final Answer:** Answer: A. Delay in surgical intervention >24 hours.	**Annotation:** Correct

Q52. NEJM Image Challenge 10/13/22

A 19-year-old woman presented with a 6-month history of a slowly growing asymptomatic dark spot on her left palm. Examination was notable for a nonscaling, nonpalpable brown patch (left). Dermatoscopy revealed pigmented spicules, and palmar skin scrapings were prepared (right). What is the most likely diagnosis?

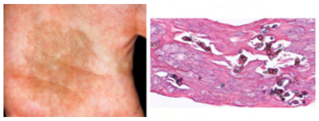

A. Acral lentiginous melanoma
B. Lichen planus
C. Secondary syphilis
D. Tinea nigra
E. Tinea versicolor

Correct Answer: Tinea nigra
Explanation: The correct answer is tinea nigra. Tinea nigra is a dematiaceous fungal infection typically seen in tropical climates. It most commonly manifests as a hyperpigmented patch on the palm of the hand and may be mistaken for a melanocytic lesion, such as melanoma. After treatment with topical butenafine, the patient's symptoms had abated at 3 months of follow-up.

GPT-4V Answer

**Image Comprhension:** The provided image is a two-part visual representation. On the left, we see a close-up of a portion of the palm of a hand, which displays a non-scaling, non-palpable brownish patch. The skin around it appears normal without any signs of inflammation or other evident pathology. The image on the right appears to be a microscopic view of a skin scraping, showing a pinkish background with multiple darkly pigmented structures interspersed. These structures are of various shapes and sizes, some being elongated and spiculate.	**Annotation:** Correct<br>**Comment:** Good recognition of both clinical and pathology images
**Recall of Medical Knowledge:** Acral lentiginous melanoma: A subtype of melanoma that occurs on the palms, soles, and under the nails. It can present as a slowly enlarging dark patch. Under the microscope, atypical melanocytes are seen invading the epidermis and dermis. Lichen planus: A skin condition that can cause purplish, itchy, flat-topped bumps. However, it doesn't typically manifest as a single dark patch on the palm. Secondary syphilis: Can present with a variety of skin manifestations, including a generalized rash that can involve the palms. Microscopically, spirochetes can be seen. Tinea nigra: A superficial fungal infection that results in dark, non-scaling patches primarily on the palms and soles. Under the microscope, brownish septate hyphae and spores are observed. Tinea versicolor: A superficial fungal infection caused by Malassezia species. It usually presents as hypo- or hyperpigmented patches on the trunk and proximal extremities but is uncommon on the palms. Microscopy would show "spaghetti and meatballs" appearance due to hyphae and yeast cells.	**Annotation:** Correct<br>**Comment:** None
**Step-by-step Reasoning:** The clinical presentation of a slowly growing, asymptomatic dark patch on the palm, along with the dermatoscopic finding of pigmented spicules, leads us to consider conditions that typically manifest in this way. Lichen planus and tinea versicolor are less likely given the palm location and the absence of the typical clinical or microscopic features associated with these conditions. While secondary syphilis can involve the palms, the dermatoscopic and microscopic features shown don't suggest the presence of spirochetes. Acral lentiginous melanoma is a serious consideration for any dark patch on acral surfaces, but the microscopic image does not show atypical melanocytes. Tinea nigra is known to cause asymptomatic, dark, non-scaling patches on the palms and soles. The microscopic image is consistent with the presence of brownish septate hyphae and spores characteristic of this condition.	**Annotation:** Correct<br>**Comment:** None
**Final Answer:** Based on the above reasoning, the most likely diagnosis is D. Tinea nigra.	**Annotation:** Correct

Q53. NEJM Image Challenge 10/6/22

A 34-year-old woman presented with a 3-year history of changes in skin color. Examination was notable for hypopigmented macules on a background of hyperpigmentation, creating a raindrop-like appearance on the chest and back. Neighbors who drank water from the same well had similar skin changes. What is the most likely diagnosis?

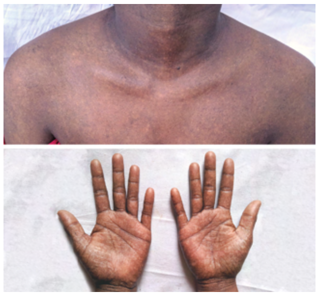

A. Arsenic toxicity
B. Cadmium toxicity
C. Copper toxicity
D. Lead toxicity
E. Mercury toxicity

Correct Answer: Arsenic toxicity
Explanation: Laboratory testing revealed elevated levels of arsenic in the patient's fingernails and hair, as well as in her well water. The patient was advised to find an alternative water source, and the contaminated well was sealed. At a 3-month follow-up visit, her skin changes had abated.

GPT-4V Answer

**Image Comprhension:** The image displays the upper chest area of an individual and a close-up of their hands. The skin on the chest reveals hypopigmented spots (lighter patches) on a hyperpigmented background (darker skin tone), resembling raindrop patterns. The hands exhibit a rough texture and discoloration.	**Annotation:** Correct<br>**Comment:** None
**Recall of Medical Knowledge:** Arsenic toxicity, also known as arsenicosis, occurs when a person ingests, breathes, or absorbs elevated levels of arsenic. Chronic arsenic exposure can result in a variety of skin manifestations, including hyperpigmentation and hypopigmentation, giving a "raindrop" appearance on the skin. In severe cases, rough, wart-like growths can appear on the palms and soles. The source of arsenic exposure can often be contaminated groundwater, used for drinking and cooking.<br>The other toxins listed, such as cadmium, copper, lead, and mercury, can cause toxicity and various symptoms when ingested in large amounts. However, their clinical presentations don't typically match the described raindrop appearance of hyperpigmentation with hypopigmented macules on the skin. Furthermore, widespread similar skin changes among neighbors who drank water from the same well point towards a contaminant present in the groundwater.	**Annotation:** Correct<br>**Comment:** None
**Step-by-step Reasoning:** The raindrop appearance of hyperpigmentation with hypopigmented macules on the skin, as depicted in the image and described, is characteristic of chronic arsenic exposure. Arsenic can contaminate groundwater, which when consumed, leads to arsenic toxicity. Given that multiple neighbors who drank from the same well exhibit similar skin changes, it indicates that the well water is likely the source of exposure. Combining the characteristic skin presentation and the shared water source among affected individuals, arsenic in the groundwater is the most plausible culprit.	**Annotation:** Correct<br>**Comment:** None
**Final Answer:** Based on the information provided and the reasoning above, the most likely diagnosis is: A. Arsenic toxicity.	**Annotation:** Correct

Q54. NEJM Image Challenge 9/29/22

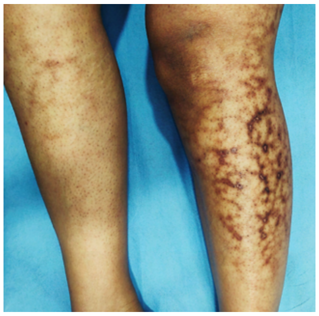

A 24-year-old woman presented with a 2-month history of rash on her shins. The rash was asymptomatic and was more prominent in the left leg. It had first appeared during the winter, at which time she had been sitting by an electric heater that was closer to her left leg. Exam was without palpable purpura, subcutaneous nodules, or areas of gangrene. What is the diagnosis?

A. Cryoglobulinemia
B. Cutaneous polyarteritis nodosa
C. Erythema ab igne
D. Livedo reticularis
E. Livedoid vasculitis

Correct Answer: Erythema ab igne
Explanation: The correct answer is erythema ab igne. Erythema ab igne is a dermatosis that results from long-term heat exposure. It manifests as an erythematous or hyperpigmented reticulation that can occur anywhere on the body, depending on the location of the external heat source. With removal of direct heat exposure, the hyperpigmentation improved.

GPT-4V Answer

**Image Comprhension:** The image depicts two shins against a blue background. The right shin shows relatively clear skin with very faint mottling or pigmentation. The left shin, however, has noticeable reticulated or net-like hyperpigmentation, which is more concentrated and darker in color compared to the right shin.	**Annotation:** Correct<br>**Comment:** None
**Recall of Medical Knowledge:** Cryoglobulinemia: Characterized by the presence of cryoglobulins in the blood, it can cause a variety of symptoms including skin manifestations like purpura, which are not noted in the exam. Cutaneous polyarteritis nodosa: A form of vasculitis affecting small and medium-sized arteries in the skin. It can present as nodules, ulcers, or livedo reticularis. Erythema ab igne: A skin condition characterized by localized areas of reticulated erythema and hyperpigmentation due to chronic exposure to infrared radiation (such as from heating pads, heaters, etc.). The pattern is often net-like. Livedo reticularis: A mottled reticulated vascular pattern on the skin that can be caused by various conditions, not just localized heat exposure. Livedoid vasculitis: A chronic vascular condition with ulcerations and irregular scars.	**Annotation:** Correct<br>**Comment:** None
**Step-by-step Reasoning:** Given the history of sitting by an electric heater with the rash being more prominent on the leg that was closer to the heater, it's suggestive of a condition caused by prolonged heat exposure. The image does not show palpable purpura, nodules, or ulcers. This rules out cryoglobulinemia, cutaneous polyarteritis nodosa, and livedoid vasculitis. The hyperpigmentation pattern is reticulated and localized, which is in line with erythema ab igne, especially given the heat exposure history. Though livedo reticularis can have a similar appearance, it is typically not localized to one area and is not related to heat exposure.	**Annotation:** Correct<br>**Comment:** None
**Final Answer:** Based on the image, history, and recalled medical knowledge, the diagnosis is most likely: C. Erythema ab igne.	**Annotation:** Correct

Q55. NEJM Image Challenge 9/22/22

A 40-year-old man presented to the dermatology clinic with a 1-year history of painless swelling of his earlobes. He also reported nasal congestion and intermittent nosebleeds. Which of the following is the most likely diagnosis?

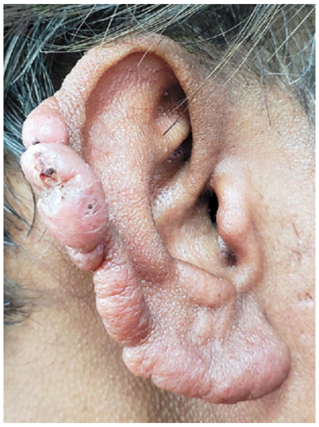

A. Cauliflower ear
B. Eosinophilic Granulomatosis and Polyangiitis
C. Lepromatous Leprosy
D. Malignant Otitis Externa
E. Relapsing Polychondritis

Correct Answer: Lepromatous Leprosy
Explanation: The correct answer is lepromatous leprosy. Dermal fluid was obtained from an incision in the earlobe for a skin smear, which was stained with the use of the modified Ziehl–Neelsen method and examined under an oil-immersion lens. The skin smear showed innumerable acid-fast bacilli. On examination, thickening of the greater auricular nerve and patchy loss of sensation in the distal limbs were also noted. A diagnosis of lepromatous leprosy, with involvement of the skin, nerves, and nasal mucosa, was made.

GPT-4V Answer

Image Comprhension: The image shows a close-up view of an ear with significant and noticeable swelling of the earlobes. There appear to be nodular changes in the earlobe with some areas having a somewhat shiny and smooth appearance. Additionally, there are some whitish and scaly patches present on the skin surrounding the ear. There's no visible sign of trauma, piercing, or injury to the ear.

Annotation: Correct
Comment: Noticing lack of sparing of ear lobes (rules out polychondritis)

Recall of Medical Knowledge: Cauliflower ear is usually a result of trauma to the ear, especially seen in wrestlers or boxers. It leads to a deformity of the outer ear due to clot formation which, if not treated, can lead to fibrosis and the classic "cauliflower" appearance. The condition is generally painful. Eosinophilic Granulomatosis and Polyangiitis (EGPA) is a systemic condition characterized by asthma, sinusitis, and an elevated eosinophil count. Skin manifestations are also present, but the specific swelling of earlobes is not typical. Lepromatous Leprosy is a form of leprosy characterized by nodular skin lesions, nasal congestion, and sometimes limb deformities. The skin lesions can affect the earlobes and cause them to swell. Nosebleeds and nasal congestion are also consistent with nasal involvement in leprosy. Malignant Otitis Externa is an aggressive but rare infection of the external ear canal. This would typically cause pain, drainage, and could potentially affect the cranial nerves. The earlobe swelling is not a common manifestation. Relapsing Polychondritis is an autoimmune condition that affects cartilaginous structures in the body, including the ears, nose, and respiratory tract. Patients can present with recurrent episodes of inflammation in these cartilaginous structures, leading to pain and swelling.

Annotation: Correct
Comment: None

Step-by-step Reasoning: Given the presentation, the main clues are painless swelling of the earlobes, nasal congestion, and intermittent nosebleeds.
The absence of trauma and the specific appearance of the ear rules out Cauliflower ear. EGPA does not specifically cause earlobe swelling as described. Malignant Otitis Externa is typically painful and does not cause isolated earlobe swelling. While Relapsing Polychondritis can cause ear symptoms, it's typically painful, which is not consistent with the painless swelling described. This leaves us with Lepromatous Leprosy. The patient's symptoms of painless earlobe swelling, nasal congestion, and intermittent nosebleeds, combined with the image showing swollen earlobes with nodular changes, are consistent with Lepromatous Leprosy.

Annotation: Correct
Comment: None

Final Answer: Answer: C. Lepromatous Leprosy.

Annotation: Correct

Q56. NEJM Image Challenge 9/15/22

A 58-year-old man with coronary artery disease presented to the emergency department with a 1-day history of intermittent chest pain at rest. An ECG was obtained on arrival, at which time the patient reported no chest pain. Which of the following is the most likely site of the culprit lesion?

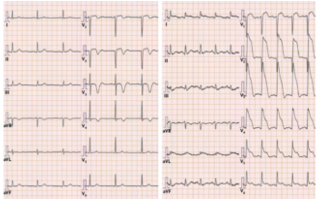

A. Right coronary artery
B. Left circumflex artery
C. Left coronary artery
D. Posterior descending artery
E. Left anterior descending artery

Correct Answer: Left anterior descending artery
Explanation: The correct answer is left anterior descending artery. The ECG raised concern for Wellens' syndrome, a pain-free period after spontaneous reperfusion of an occluded left anterior descending artery identified by biphasic or deep precordial T-wave inversions, particularly in leads V2 and V3. Shortly after being examined, the patient began having chest pain. Emergency cardiac catheterization identified a complete occlusion of the proximal left anterior descending artery.

GPT-4V Answer

**Image Comprhension:** The image provided is an electrocardiogram (ECG) that displays multiple leads of cardiac electrical activity. Observing the tracings, we can note the following:<br>There are ST-segment elevations in leads V1 to V4. There is no apparent ST-segment depression or T-wave inversion in other leads. The rhythm appears regular, and there are no evident arrhythmias on this tracing.	**Annotation:** Correct<br>**Comment:** All correct
**Recall of Medical Knowledge:** The ECG provides a graphical representation of the heart's electrical activity. Different leads "view" different parts of the heart, and changes in those leads can suggest which part of the heart might be compromised. ST-segment elevations suggest myocardial injury, commonly due to coronary artery occlusion. The leads V1 to V4 primarily represent the anterior and septal walls of the left ventricle. The blood supply to the anterior and septal walls of the left ventricle is primarily provided by the left anterior descending (LAD) artery, a major branch of the left coronary artery.	**Annotation:** Correct<br>**Comment:** All correct
**Step-by-step Reasoning:** Given the ST-segment elevations in leads V1 to V4, we suspect myocardial injury in the anterior and septal walls of the left ventricle. The area supplied by the Left Anterior Descending (LAD) artery corresponds to these ECG leads. There is no ECG evidence suggesting injury or ischemia in territories supplied by other coronary arteries. Therefore, the culprit lesion is most likely in the LAD artery, which corresponds to the anterior and septal myocardium.	**Annotation:** Correct<br>**Comment:** All correct
**Final Answer:** Answer: E. Left anterior descending artery.	**Annotation:** Correct

Q57. NEJM Image Challenge 9/8/22

A 79-year-old woman with a history of prior stroke was referred for an abnormal X-ray finding along the left heart border, first noticed 6 years prior. In the absence of symptoms, the patient was monitored with serial radiographs which showed a gradual increase in size of the finding. Which of the following is the most likely diagnosis?

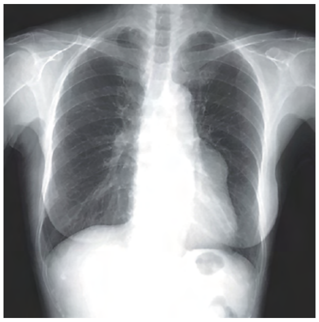

A. Giant Coronary Aneurysm
B. Left Atrial Appendage Thrombus
C. Mediastinal Hematoma
D. Pericardial Cyst
E. Teratoma

Correct Answer: Giant Coronary Aneurysm
Explanation: The correct answer is giant coronary aneurysm. Subsequent coronary computed tomography revealed an aneurysm measuring 45 mm in the greatest dimension arising from the left coronary artery, consistent with a diagnosis of coronary artery aneurysm. Following cardiac catheterization, which revealed an additional fistula, the patient underwent cardiac surgery and was discharged 16 days later.

GPT-4V Answer	
**Image Comprhension:** The image appears to be a chest X-ray showing the thoracic region, including the lungs, heart border, and rib cage. There is an abnormal finding along the left heart border, which is rounded, well-circumscribed, and has a distinct boundary. This finding is consistent with some form of a mass or growth.	**Annotation:** Correct<br>**Comment:** All correct
**Recall of Medical Knowledge:** Giant Coronary Aneurysm: This is a dilatation of a segment of a coronary artery that is more than 1.5 times its normal size. It may or may not produce symptoms. When it does, it's often chest pain or a heart attack.<br>Left Atrial Appendage Thrombus: This is a blood clot in the left atrial appendage. Patients with atrial fibrillation are at increased risk for this condition. It can lead to strokes.<br>Mediastinal Hematoma: This is a collection of blood in the mediastinum, which is the space between the lungs. It usually results from trauma or surgery.<br>Pericardial Cyst: This is a rare, benign condition. It's a fluid-filled sac that develops in the pericardium, which is the thin sac-like membrane surrounding the heart. They are usually found incidentally on imaging and are typically asymptomatic.<br>Teratoma: This is a type of germ cell tumor that can contain several different types of tissue, such as hair, muscle, or bone. They are most often found in the ovaries in women and the testicles in men but can occur in other parts of the body.	**Annotation:** Correct<br>**Comment:** All correct
**Step-by-step Reasoning:** The described growth has been present for 6 years and has been increasing in size. This duration of stability argues against an acute process like a hematoma or thrombus.<br>Given the lack of symptoms, a benign lesion is more probable. Mediastinal hematomas and thrombi would be expected to produce symptoms over such a long duration.<br>A coronary aneurysm would be extremely rare to reach a size where it's visible as a distinct mass on a chest X-ray and would be expected to produce cardiac symptoms.<br>Teratomas in the mediastinum are usually anterior and not along the heart border.<br>This leaves us with a Pericardial Cyst. They are often discovered incidentally on imaging, can grow over time, and may not produce symptoms. They are located where this mass appears to be, along the heart border.	**Annotation:** Correct<br>**Comment:** All correct
**Final Answer:** Answer: D.Pericardial Cyst.	**Annotation:** Correct

Q58. NEJM Image Challenge 9/1/22

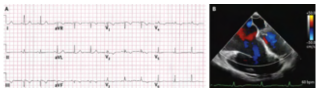

A 59-year-old woman was admitted to the hospital with unheralded syncope. She had lost a son to sudden cardiac death when he was 29 years of age. On hospital day 2, the patient developed sustained monomorphic ventricular tachycardia treated with synchronized cardioversion and intravenous amiodarone. An electrocardiogram was performed after the event (Panel A). A transesophageal echocardiogram showed a severely dilated right ventricle (Panel B). Cardiac magnetic resonance imaging showed a right ventricular ejection fraction of 27

A. Arrhythmogenic right ventricular cardiomyopathy
B. Brugada Syndrome
C. Cardiac sarcoidosis
D. Inferior Myocardial Infarction
E. Pulmonary Embolism

Correct Answer: Arrhythmogenic right ventricular cardiomyopathy
Explanation: A diagnosis of arrhythmogenic right ventricular cardiomyopathy (ARVC) was made. The ECG showed epsilon waves (discrete small amplitude potentials occurring after the QRS complex has returned to the iso-electric line), which are a major diagnostic criterion in the diagnosis of this condition. An implantable cardioverter-defibrillator was placed, and treatment with metoprolol and anti-arrhythmic medication was initiated.

GPT-4V Answer

**Image Comprhension:** Panel A depicts an electrocardiogram (ECG) with 12 leads. The ECG shows certain rhythms and patterns that are used to diagnose various heart conditions. From an initial glance, there are no evident ST-segment elevations or depressions. However, there are some anomalies in the T-waves, and certain leads may suggest right ventricular involvement.<br>Panel B shows a transesophageal echocardiogram of the heart. The image reveals a dilated right ventricle with color flow Doppler indicating blood flow direction and velocity. The blue and red colors represent the flow of blood, with each color indicating the direction of flow. There are no evident clots, masses, or other anomalies visible in this image.	**Annotation:** Correct<br>**Comment:** All correct
**Recall of Medical Knowledge:** Arrhythmogenic right ventricular cardiomyopathy (ARVC): This is a heart muscle disorder that causes fibrofatty replacement of the right ventricular myocardium. It is a genetic condition and can lead to ventricular arrhythmias and sudden cardiac death, especially in young individuals. A dilated right ventricle with regional akinesis and a familial history of sudden cardiac death are suggestive of this diagnosis.<br>Brugada Syndrome: This is an inherited condition that can cause a disruption in the heart's normal rhythm. It is associated with characteristic ECG changes, but not with dilation of the right ventricle.<br>Cardiac sarcoidosis: It is an inflammatory disease that can affect any organ, including the heart. When it affects the heart, it can lead to arrhythmias, heart block, or heart failure. However, it would typically show additional findings on imaging.<br>Inferior Myocardial Infarction: This would typically show ST elevations in the inferior leads on an ECG.<br>Pulmonary Embolism: This is a blockage in one of the pulmonary arteries in the lungs. It can strain the right side of the heart and cause right heart dilation, but there are other specific clinical and ECG findings associated with it, such as S1Q3T3 pattern.	**Annotation:** Correct<br>**Comment:** All correct
**Step-by-step Reasoning:** The clinical history mentions unheralded syncope and a family history of sudden cardiac death at a young age, both of which can be associated with ARVC. The ECG (Panel A) doesn't show the typical signs of Brugada Syndrome, Inferior Myocardial Infarction, or Pulmonary Embolism. The transesophageal echocardiogram (Panel B) clearly shows dilation of the right ventricle, which aligns with the features of ARVC. Given the combination of a dilated right ventricle, regional akinesis, and a familial history of sudden cardiac death, ARVC is the most consistent diagnosis with the provided information.	**Annotation:** Correct<br>**Comment:** All correct
**Final Answer:** Answer: A. Arrhythmogenic right ventricular cardiomyopathy.	**Annotation:** Correct

Q59. NEJM Image Challenge 8/25/22

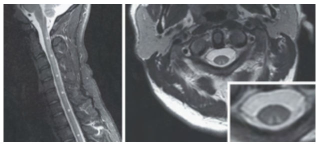

A 32-year-old man presented with a 6-week history of tingling in his arms and legs and a 2-week history of inability to walk. A positive Romberg test, sensory ataxia, impaired proprioception and vibratory sensation, and preserved nociception were noted. Magnetic resonance imaging of the whole spine showed hyperintensity in the posterior spinal cord from C1 to T12 and hyperintense lesions in the dorsal column on T2-weighted images. A vitamin B12 level was 107 pg per ml (reference value, >231) without macrocytic anemia. Toxicity of which of the following is the most likely cause of this presentation?

A. Copper
B. Nitrous oxide
C. Organophosphates
D. Tetrodotoxin (pufferfish poisoning)
E. Thallium

Correct Answer: Nitrous oxide
Explanation: The correct answer is nitrous oxide. A diagnosis of subacute combined degeneration associated with nitrous oxide use was made. Two months before presentation, the patient had begun inhaling nitrous oxide — also known as “whippets” or “laughing gas” — daily. Treatment included cessation of nitrous oxide use and cyanocobalamin replacement therapy. At 4-week follow-up, he was able to walk independently.

GPT-4V Answer	
**Image Comprhension:** The provided image appears to be a set of MRI scans (T2-weighted) showing the spinal cord. There are hyperintense (brighter) areas observed in the posterior (back) region of the spinal cord stretching from the cervical region (C1) down to the thoracic region (T12). These hyperintense areas are particularly noticeable in the dorsal column of the spinal cord.	**Annotation:** Partially correct **Comment:** The description is correct, although it says that the hyperintense lesion extends to T12 which is not visible in the image. It goes only as far as T1 or T2
**Recall of Medical Knowledge:** The dorsal column of the spinal cord is primarily responsible for transmitting proprioceptive and vibratory sensory information. Vitamin B12 deficiency can cause damage to the myelin sheath of neurons, particularly in the dorsal column, leading to sensory neuropathy. Several substances and toxins can lead to neuropathy or damage to the spinal cord. Copper: Essential for the function of many enzymes; deficiency can cause neurological symptoms but typically presents with a different pattern. Nitrous oxide (N2O): Abused recreationally and can cause oxidation of vitamin B12, rendering it inactive, leading to a deficiency state even with normal serum levels. Organophosphates: These are pesticides that can cause an acute cholinergic syndrome with symptoms like miosis, salivation, and confusion. Tetrodotoxin: Found in pufferfish and blocks sodium channels, leading to paralysis. Thallium: Causes peripheral neuropathy but has a different mechanism of action and does not typically present in this manner.	**Annotation:** Partially correct **Comment:** Neuropathy indicates injury to peripheral nerves, not spine which is indicated here. The subacute degeneration of the spine would be a sensory myelopathy. The statement "neuropathy or damage to the spinal cord" is incorrect. Myelopathy is damage to the spinal cord. Neuropathy is damage to peripheral nerves.
**Step-by-step Reasoning:** The MRI findings and clinical presentation of sensory ataxia, impaired proprioception and vibratory sensation, and preserved nociception correspond to damage in the dorsal columns of the spinal cord. Given that the patient’s vitamin B12 level is significantly below the reference range, there’s clear evidence of a B12 deficiency which is known to cause such damage. Out of the listed options, only nitrous oxide is associated with causing vitamin B12 deficiency by rendering it inactive. This would fit the described clinical and radiological findings. Thus, a history of nitrous oxide abuse or exposure might explain the clinical scenario in the context of the low vitamin B12 levels.	**Annotation:** Correct **Comment:** I would have liked to have seen the mention of the condition "subacute combined degeneration of the spinal cord" which this is. But the answer is still fully correct.
**Final Answer:** Answer: B.Nitrous oxide.	**Annotation:** Correct

Q60. NEJM Image Challenge 8/18/22

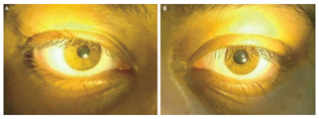

A 13-year-old girl presented with new onset blurry vision in the left eye associated with painful eye movements. Three weeks earlier, weakness and paresthesias had developed in her left leg that spontaneously resolved. When a light was swung from the normal right eye (Panel A) to the affected left eye, the left pupil dilated (Panel B) – a finding known as a relative afferent pupillary defect. Fundoscopic examination of both eyes was normal. Examination of the brain and whole spine by means of magnetic resonance imaging showed multiple, oblong hyperintense lesions on T2-weighted images. What is the most likely etiology?

A. Acute disseminated encephalomyelitis
B. Leber hereditary optic neuropathy
C. Mononeuritis multiplex
D. Multiple sclerosis
E. Retinal detachment

Correct Answer: Multiple sclerosis
Explanation: The correct answer is multiple sclerosis. Serum tests for aquaporin-4 and myelin oligodendrocyte glycoprotein antibodies were negative. Oligoclonal bands were present in the cerebrospinal fluid. Symptoms resolved after the initiation of steroids followed by maintenance therapy with interferon beta-1a

---

GPT-4V Answer

**Image Comprhension:** The image presents two panels, A and B, showing close-up views of human eyes. Panel A displays an eye (presumably the right eye) that appears normal with a constricted pupil. Panel B shows a different eye (presumably the left eye) with a dilated pupil, suggesting a relative afferent pupillary defect when light is swung from the right eye to the left eye.	**Annotation:** Correct<br>**Comment:** None
**Recall of Medical Knowledge:** A relative afferent pupillary defect (RAPD) is indicative of a problem with the optic nerve's function. The pupil dilates instead of constricting when light is shone into it because the affected eye doesn't send the same number of signals to the brain as the healthy eye. Acute disseminated encephalomyelitis (ADEM) is an inflammatory demyelinating disease that predominantly affects the white matter of the brain and spinal cord. Leber hereditary optic neuropathy (LHON) is a maternally inherited condition leading to vision loss. Mononeuritis multiplex involves isolated damage to at least two separate nerve areas. Multiple sclerosis (MS) is an autoimmune disorder characterized by inflammation, demyelination, and axonal damage. Symptoms can be intermittent, and MRI often shows hyperintense lesions on T2-weighted images. Retinal detachment involves the separation of the retina from the underlying layer.	**Annotation:** Correct<br>**Comment:** This is interesting. It is different from prior demonstrations of medical knowledge recall where it had listed information about all the choices. Instead, it is describing information about the findings on imaging combined with the information from the history as it relates to manganese toxicity.
**Step-by-step Reasoning:** Considering the MRI findings of multiple, oblong hyperintense lesions on T2-weighted images, the clues point towards a demyelinating process. The history of weakness and paresthesias in her left leg that spontaneously resolved indicates an intermittent nature to the symptoms. LHON typically doesn't present with MRI changes and doesn't cause RAPD. Mononeuritis multiplex primarily affects peripheral nerves, not the central nervous system, and wouldn't cause the MRI findings mentioned. ADEM usually follows an infection and often has a more fulminant and widespread clinical presentation than described. Retinal detachment would not be associated with neurological symptoms or MRI findings of hyperintense lesions. Given the relative afferent pupillary defect, the neurological symptoms, and the MRI findings, Multiple sclerosis (MS) fits best.	**Annotation:** Correct<br>**Comment:** None
**Final Answer:** D. Multiple sclerosis.	**Annotation:** Correct

Q61. NEJM Image Challenge 8/11/22

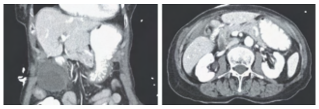

An 86-year-old woman presented with 1 day of nausea, vomiting, and right-sided abdominal pain. Computed tomography of the abdomen and pelvis revealed a distended gallbladder with a thickened wall outside the gallbladder fossa. There was also swirling of the cystic artery and duct but no dilatation of the common bile duct. Which of the following is most likely diagnosis?

A. Acalculous cholecystitis
B. Cholangiocarcinoma
C. Cholangitis
D. Ectopic gallbladder
E. Gallbladder volvulus

Correct Answer: Gallbladder volvulus
Explanation: Gallbladder volvulus was confirmed on laparoscopy when a free-floating, necrotic gallbladder was observed encircling the cystic artery and duct. The surgery was converted to an open procedure, and detorsion and resection of the gallbladder were successfully performed.

GPT-4V Answer

**Image Comprhension:** The provided images appear to be computed tomography (CT) scans of the abdomen. From the description and the scans:<br>The gallbladder looks distended and is located outside its usual anatomical fossa. The wall of the gallbladder appears thickened. There's swirling of the cystic artery and duct, indicating a potential torsion or twist. There is no noted dilatation of the common bile duct.	**Annotation:** Correct<br>**Comment:** None
**Recall of Medical Knowledge:** Acalculous cholecystitis is an inflammation of the gallbladder without the presence of gallstones. It typically presents with right upper quadrant pain, fever, and leukocytosis. A thickened gallbladder wall and pericholecystic fluid can be seen on imaging. Cholangiocarcinoma is a malignancy of the bile ducts. On imaging, it can present as a mass or stricture in the bile ducts, often with bile duct dilation. Cholangitis is an infection of the bile ducts, often due to an obstructing gallstone. It typically presents with the Charcot's triad of fever, jaundice, and right upper quadrant pain. Imaging would typically show dilated bile ducts. Ectopic gallbladder is a congenital anomaly where the gallbladder is located outside its normal anatomical position. However, it doesn't typically present acutely in the elderly. Gallbladder volvulus or torsion is a rare condition where the gallbladder rotates on its mesentery, leading to obstruction of the cystic duct and artery. This can cause ischemia and necrosis. It presents acutely with symptoms similar to cholecystitis. The swirling of the cystic duct and artery on imaging is suggestive of torsion.	**Annotation:** Correct<br>**Comment:** None
**Step-by-step Reasoning:** The patient presents acutely with nausea, vomiting, and right-sided abdominal pain. This presentation can fit several of the conditions listed. The imaging shows a distended gallbladder with a thickened wall located outside the gallbladder fossa. This can be seen in both acalculous cholecystitis and gallbladder volvulus. The absence of dilatation of the common bile duct makes cholangitis and cholangiocarcinoma less likely. The key distinguishing feature here is the swirling of the cystic artery and duct, which is indicative of torsion or twisting, making gallbladder volvulus the most likely diagnosis.	**Annotation:** Correct<br>**Comment:** None
**Final Answer:** Answer: E. Gallbladder volvulus.	**Annotation:** Correct

Q62. NEJM Image Challenge 8/4/22

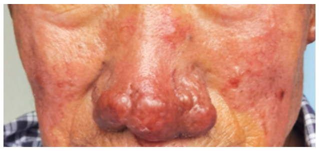

A 71-year-old man presented with an 8-year history of enlarging nodules on his nose. On physical examination, he had painless, violaceous, indurated nodules on his nose, ears, fingers, and toes. Computed tomography of the chest revealed hilar and mediastinal lymphadenopathy. Skin biopsy showed non-caseating granulomas. What is the most likely diagnosis?

A. Entomophthoramycosis
B. Leprosy
C. Lupus pernio
D. Lupus vulgaris
E. Rhinophyma

Correct Answer: Lupus pernio
Explanation: A diagnosis of sarcoidosis with lupus pernio was made. Treatment with systemic glucocorticoid therapy was initiated, and at a follow-up visit 6 months after presentation, the nodules had abated.

GPT-4V Answer

**Image Comprhension:** The image displays a close-up view of what appears to be a section of an elderly person's face, specifically the nose. The skin on the nose shows pronounced, reddish-purple, raised nodules that are variably sized. The skin surrounding these nodules appears to be slightly thickened and discolored, with a reddish hue. No other distinct facial features can be identified from the image.	**Annotation:** Correct<br>**Comment:** None
**Recall of Medical Knowledge:** Entomophthoramycosis: A rare fungal infection usually involving the nasal sinuses, orbit, and brain. The skin can be involved but is not typically characterized by violaceous nodules. Leprosy: A chronic infectious disease caused by Mycobacterium leprae, it affects the skin and peripheral nerves. Depending on the form, skin manifestations can range from hypopigmented patches to nodular lesions, but the presentation does not typically match the described violaceous nodules on multiple sites, such as fingers and toes. Lupus pernio: A form of cutaneous sarcoidosis characterized by indurated, purple or violaceous nodules and plaques predominantly on the face, particularly the nose, ears, lips, and cheeks. It is not infectious. Sarcoidosis can also manifest with non-caseating granulomas on biopsy and lymphadenopathy on imaging. Lupus vulgaris: A chronic and progressive form of cutaneous tuberculosis. It manifests as reddish-brown nodules but is generally limited to the skin without causing systemic symptoms. Rhinophyma: A subtype of rosacea leading to thickening of the skin of the nose, primarily affecting the sebaceous glands. It does not have systemic manifestations.	**Annotation:** Correct<br>**Comment:** None
**Step-by-step Reasoning:** Given the clinical picture:<br>The patient has violaceous, indurated nodules on his nose, ears, fingers, and toes. CT shows hilar and mediastinal lymphadenopathy, suggesting a systemic process. Skin biopsy shows non-caseating granulomas. Based on the above points and the recalled medical knowledge:<br>The non-caseating granulomas on biopsy, combined with the violaceous nodules on the nose, ears, fingers, and toes, and the hilar and mediastinal lymphadenopathy on CT imaging, are characteristic of sarcoidosis. The skin manifestation of sarcoidosis described in the scenario is Lupus pernio.	**Annotation:** Correct<br>**Comment:** None
**Final Answer:** Hence, the most likely diagnosis for this patient is C. Lupus pernio.	**Annotation:** Correct

Q63. NEJM Image Challenge 7/28/22

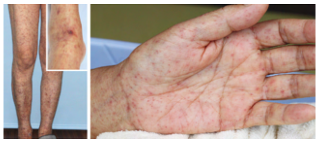

A 68-year-old woman living in Japan presented to the clinic with a 5-day history of fevers, myalgias, and petechial rash spreading on her arms, trunk, palms, and soles. On exam, there was also an eschar on the right medial ankle. Laboratory studies showed thrombocytopenia, elevated inflammatory markers, and elevated liver transaminases. Which of the following is the most appropriate treatment?

A. Antihistamine
B. Glucocorticoid
C. Observation
D. Tetracycline
E. Valacyclovir

Correct Answer: Tetracycline
Explanation: Polymerase-chain-reaction testing of a biopsy specimen of the eschar detected Rickettsia japonica. A diagnosis of Japanese spotted fever was made. After the patient completed a 2-week course of minocycline therapy, her symptoms resolved.

GPT-4V Answer	
**Image Comprhension:** The image displays areas of petechial rash on the lower legs and the hands of an individual. Petechiae are small, round, red or purple spots that appear as a result of bleeding under the skin. The rash seems to be more pronounced on the palms and fingers. There's also an enlarged view of an area on the leg, possibly the ankle, that appears to show an eschar, which is a dry, dark scab or falling away of dead skin.	**Annotation:** Correct<br>**Comment:** None
**Recall of Medical Knowledge:** Petechial rash, especially on the palms and soles, is commonly associated with certain infections like Rocky Mountain spotted fever, secondary syphilis, or certain viral infections.<br>An eschar is typically associated with certain infections like anthrax or scrub typhus (also known as Orientia tsutsugamushi infection). Scrub typhus is common in the Asia-Pacific region, including Japan. The disease is transmitted by the bite of an infected chigger (larval mite). The bite often leaves an eschar.<br>The clinical symptoms mentioned, along with the laboratory findings, are suggestive of an infectious process, possibly scrub typhus given the presence of an eschar and the geographical location (Japan).<br>Treatment for scrub typhus involves the use of antibiotics, specifically tetracycline or doxycycline. It's important to initiate treatment early to prevent complications.	**Annotation:** Partially correct<br>**Comment:** Rickettsia japonica and orientia tsutsugamushi can cause very similar clinical syndromes and they are related; based on the clinical presentation it is hard to distinguish those two. Treatment is the same.
**Step-by-step Reasoning:** The patient's presentation of fever, myalgia, petechial rash on the palms and soles, and an eschar is suggestive of scrub typhus, especially given her location in Japan. The laboratory findings, including thrombocytopenia, elevated inflammatory markers, and elevated liver transaminases, further support this diagnosis. Based on the knowledge that scrub typhus is treated with antibiotics, specifically tetracycline or doxycycline, the appropriate treatment in this scenario would be tetracycline.	**Annotation:** Partially correct<br>**Comment:** As above, Rickettsia japonica and orientia tsutsugamushi are very similar.
**Final Answer:** Answer: D.Tetracycline.	**Annotation:** Correct

Q64. NEJM Image Challenge 7/21/22

A 23-year-old man presented with a 1-month history of double vision and right eyelid drooping that worsened at the end of the day. On examination, manual raising of the ptotic right eyelid resulted in dropping of the left eyelid. What is the most likely diagnosis?

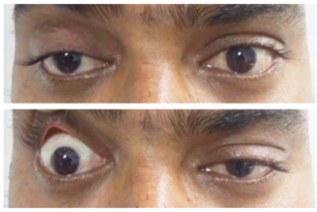

A. Botulism
B. Horner's syndrome
C. Miller Fisher syndrome
D. Multiple sclerosis
E. Myasthenia gravis

Correct Answer: Myasthenia gravis
Explanation: This finding – known as the curtain sign, or enhanced ptosis – is characteristically seen in myasthenia gravis. Repetitive nerve stimulation showed a decremental response, and acetylcholine receptor antibody levels were elevated. Treatment with pyridostigmine and prednisolone was initiated.

GPT-4V Answer

**Image Comprhension:** The image displays two close-up shots of a man's eyes. In the first image, the right eye appears to have a drooping eyelid (ptosis), while the left eye appears normal. In the second image, when the right eyelid is manually elevated, the left eyelid droops.	**Annotation:** Correct<br>**Comment:** None
**Recall of Medical Knowledge:** Botulism: Typically presents with descending paralysis starting with cranial nerve deficits. Double vision can occur due to paralysis of the muscles controlling the eye. However, there is usually a history of ingestion of contaminated food. Horner's syndrome: Presents with miosis (constricted pupil), ptosis (drooping eyelid), and anhidrosis (lack of sweating). It does not typically present with symptoms in both eyes or with the symptom of one eyelid drooping when the other is raised. Miller Fisher syndrome: A variant of Guillain-Barre syndrome that presents with ophthalmoplegia (eye muscle weakness), ataxia (lack of muscle control), and areflexia (absence of reflexes). Double vision can occur due to the ophthalmoplegia. Multiple sclerosis: A demyelinating disease that can present with a myriad of neurological symptoms. Optic neuritis is a common eye-related symptom, but the presentation does not match closely with the given case. Myasthenia gravis: An autoimmune disorder that results in muscle weakness. Ocular symptoms can include ptosis and double vision. The weakness tends to worsen with muscle use and can improve with rest. A hallmark sign is the "curtain sign" or "peek sign" where raising one droopy eyelid results in the dropping of the other eyelid.	**Annotation:** Correct<br>**Comment:** None
**Step-by-step Reasoning:** Given the patient's symptoms of double vision, right eyelid drooping that worsens throughout the day, and the "curtain sign" depicted in the image, the diagnosis that most closely matches this presentation is Myasthenia gravis. The other options, although they can present with some of the symptoms, do not align as closely with the entire clinical picture provided.	**Annotation:** Correct<br>**Comment:** It could have elaborated more on why the other options didn't align as closely with the entire clinical picture like how the previous answer provided information about each option. But it is nonetheless correct.
**Final Answer:** Answer: E. Myasthenia gravis.	**Annotation:** Correct

Q65. NEJM Image Challenge 7/14/22

A 26-year-old woman with recurrent tonsillitis presents with progressive sore throat and pain with swallowing. She was febrile and had a white-cell count of 19,450 cells per cubic millimeter. CT scan of the neck is shown. What is the diagnosis?

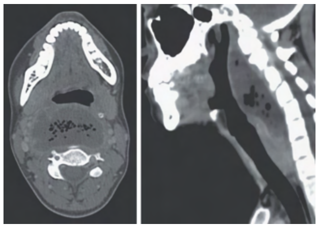

A. Epiglottitis
B. Peritonsillar abscess
C. Retropharyngeal abscess
D. Septic thrombophlebitis of the internal jugular vein (Lemierre's syndrome)
E. Submandibular space infection (Ludwig's angina)

Correct Answer: Retropharyngeal abscess
Explanation: The correct answer is retropharyngeal abscess. She underwent urgent surgical drainage and received treatment with intravenous broad-spectrum antimicrobials given the life-threatening nature of this condition. Fluid cultures grew Fusobacterium necrophorum. Her symptoms abated and she was discharged on oral antimicrobial agents

GPT-4V Answer	
**Image Comprhension:** The CT scan images of the neck show thickening and swelling in the tissue around the pharynx. There appears to be a fluid-filled space or abscess posterior to the pharynx. There is also asymmetry between the left and right sides of the oropharyngeal region.	**Annotation:** Correct<br>**Comment:** None
**Recall of Medical Knowledge:** Epiglottitis is inflammation of the epiglottis. CT would show a thickened, "thumb-like" epiglottis. It is primarily a pediatric disease, although it can be seen in adults. A peritonsillar abscess is a collection of pus between the tonsil and the pharyngeal wall. It manifests as a unilateral swelling with deviation of the uvula to the opposite side. A retropharyngeal abscess is a deep neck space infection that affects the space behind the pharynx. CT would show thickening and fluid collection in the retropharyngeal space. Septic thrombophlebitis of the internal jugular vein, also known as Lemierre's syndrome, is typically associated with a preceding pharyngeal infection. Fusobacterium necrophorum is the primary pathogen. It leads to septicemia and can be complicated by septic pulmonary emboli. CT may show an enlarged, thrombosed internal jugular vein. Ludwig's angina is an infection of the submandibular space, characterized by bilateral swelling of the submandibular and sublingual spaces. This swelling can be life-threatening if it impinges on the airway. CT would show swelling in the aforementioned spaces.	**Annotation:** Correct<br>**Comment:** None
**Step-by-step Reasoning:** The "thumb-like" epiglottis typical of epiglottitis is not clearly visible, so A is less likely. There's no clear deviation of the uvula or a distinct abscess formation lateral to the tonsils, making option B less likely. The image does display swelling and possible fluid collection in the retropharyngeal space, which is consistent with a retropharyngeal abscess. This aligns with the patient's clinical presentation of a sore throat, pain with swallowing, and elevated white blood cell count. There is no clear evidence of thrombosis in the internal jugular vein or septic pulmonary emboli to suggest Lemierre's syndrome, making option D less probable. Ludwig's angina would typically present with more pronounced bilateral swelling in the submandibular region, which is not evident in the image, making option E unlikely.	**Annotation:** Correct<br>**Comment:** None
**Final Answer:** Given the findings and reasoning, the most probable diagnosis based on the CT scan and the clinical information provided is: C. Retropharyngeal abscess.	**Annotation:** Correct

Q66. NEJM Image Challenge 7/7/22

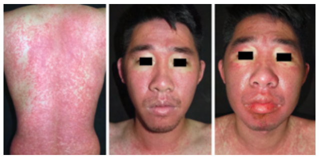

A 30-year-old man was admitted to the hospital with 2 weeks of rash and fever that had started one month after taking a course of trimethoprim–sulfamethoxazole for the treatment of folliculitis. Physical examination was notable for fever, a diffuse morbilliform rash, submandibular lymphadenopathy, and facial erythema with periorbital sparing. Four days after admission, facial edema developed. Labs showed elevated absolute eosinophils and aminotransferase levels. What's the most likely diagnosis?

A. Acute generalized exanthematous pustulosis
B. Drug reaction with eosinophilia and systemic symptoms
C. Erythroderma
D. Hypereosinophilic syndrome
E. Stevens-Johnson syndrome/toxic epidermal necrolysis

Correct Answer: Drug reaction with eosinophilia and systemic symptoms
Explanation: The correct answer is drug reaction with eosinophilia and systemic symptoms. He was treated with systemic glucocorticoids and cyclosporine with improvement of his symptoms and was advised to avoid sulfonamide-containing antibiotics in the future.

GPT-4V Answer

Image Comprhension: The images provided depict an individual with widespread skin manifestations. The features observed include:
A diffuse morbilliform rash evident on the back. The rash is erythematous (red), with areas of confluent redness and patchy distribution. Facial images show prominent facial erythema, especially involving the cheeks and forehead. The erythema appears to have spared the area around the eyes, commonly referred to as "periorbital sparing." The facial images also display the development of facial edema, with the lips appearing swollen, and the presence of serous exudate.

Annotation: Correct
Comment: None

Recall of Medical Knowledge: Acute generalized exanthematous pustulosis (AGEP): This is a rare severe cutaneous adverse reaction characterized by the rapid appearance of numerous small, non-follicular, sterile pustules arising on a background of generalized edema and erythema. Fever and neutrophilia are common.
Drug reaction with eosinophilia and systemic symptoms (DRESS): It is a severe drug hypersensitivity reaction characterized by a prolonged latency period, followed by fever, rash, eosinophilia, and systemic symptoms, including lymphadenopathy, facial edema, and internal organ involvement. Some common culprits include antiepileptics, allopurinol, and sulfa drugs, including trimethoprim-sulfamethoxazole.
Erythroderma: This refers to a generalized redness and scaling of the skin. It is not a diagnosis in itself but rather a reaction pattern seen in various conditions like psoriasis, eczema, and drug reactions.
Hypereosinophilic syndrome: A group of conditions characterized by a persistently increased number of eosinophils in the blood, associated with damage to organs. The skin, heart, and nervous system are the most commonly affected organs.
Stevens-Johnson syndrome/toxic epidermal necrolysis (SJS/TEN): This is a severe, life-threatening mucocutaneous reaction, most commonly triggered by medications, which is characterized by widespread epidermal necrosis and detachment.

Annotation: Correct
Comment: None

Step-by-step Reasoning: Given the provided clinical details and the image comprehension:
The patient was recently exposed to trimethoprim-sulfamethoxazole, a known drug that can cause hypersensitivity reactions. The morbilliform rash, facial erythema with periorbital sparing, facial edema, and the labs indicating eosinophilia and elevated aminotransferase levels are all characteristic of DRESS. Additionally, the latency period (rash starting one month after drug exposure) also fits DRESS. The absence of numerous small pustules makes AGEP less likely. The rash doesn't resemble the severe skin sloughing seen in SJS/TEN. Erythroderma is a descriptive term, and given the other symptoms, it's more likely to be a manifestation of another condition (like DRESS) rather than the primary diagnosis. There's no mention of other organ systems being affected in a way that would suggest hypereosinophilic syndrome.

Annotation: Correct
Comment: None

Final Answer: Based on the above reasoning, the most likely diagnosis is: B. Drug reaction with eosinophilia and systemic symptoms (DRESS).

Annotation: Correct

Q67. NEJM Image Challenge 6/30/22

A 28-year-old woman with a history of ulcerative colitis and pulmonary embolism presented with a 3-day history of dry cough and pleuritic chest pain. A chest radiograph was obtained. What is the name of this imaging finding?

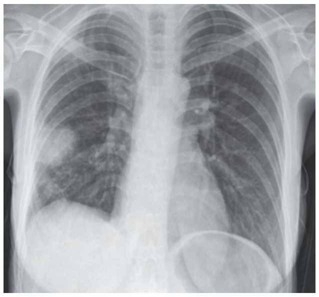

A. Fleischner sign
B. Hampton's hump
C. Palla sign
D. Spine sign
E. Westermark sign

Correct Answer: Hampton's hump
Explanation: The chest radiograph showed a dome-shaped, pleural-based opacity in the right lung, consistent with Hampton's hump. Computed tomographic pulmonary angiography revealed filling defects in the right lobar pulmonary artery and a peripheral wedge-shaped consolidation, confirming pulmonary embolism with distal pulmonary infarction. The patient started anticoagulation therapy.

GPT-4V Answer

**Image Comprhension:** The image presented is a chest radiograph, commonly referred to as a chest X-ray. It provides an anterior-posterior view of the thorax. Within the right lung, there appears to be a wedge-shaped opacity, its base towards the pleura and its apex pointing towards the hilum.	**Annotation:** Partially correct<br>**Comment:** It could be a postero-anterior view.
**Recall of Medical Knowledge:** Fleischner sign: This is the enlargement of the pulmonary artery in the upper lobe in cases of pulmonary embolism. Hampton's hump: A wedge-shaped opacity in the lung with its base on the pleura, typically seen in cases of pulmonary embolism where there is an infarct in the lung. Palla sign: Enlargement of the right descending pulmonary artery seen in patients with acute pulmonary embolism. Spine sign: On a lateral chest X-ray, it's the increased opacity of the lower thoracic spine relative to the upper thoracic spine, indicative of a lower lobe pneumonia. Westermark sign: A focal oligemia (decreased blood volume) in the lung, which is seen in pulmonary embolism.	**Annotation:** Correct<br>**Comment:** None
**Step-by-step Reasoning:** Given the clinical history and the appearance of the chest X-ray:<br>The woman has a history of pulmonary embolism, which increases the likelihood of another episode. The wedge-shaped opacity in the lung with its base on the pleura is consistent with Hampton's hump. This is a sign seen in cases of pulmonary embolism where there is an infarct in the lung tissue due to a blockage in the blood supply, often caused by a blood clot. The other signs listed, such as Fleischner, Palla, Spine, and Westermark, are not evident in this image.	**Annotation:** Correct<br>**Comment:** None
**Final Answer:** Answer: The imaging finding is B. Hampton's hump.	**Annotation:** Correct

Q68. NEJM Image Challenge 6/23/22

An 8-month-old girl presented with strabismus and developmental delay. On examination, she was noted to have macrocephaly, intermittent exotropia in the left eye, and hypotonia. Fundoscopic exam results are shown in the image. MRI of the brain revealed inadequate myelination diffusely and a thin corpus callosum. What is the diagnosis?

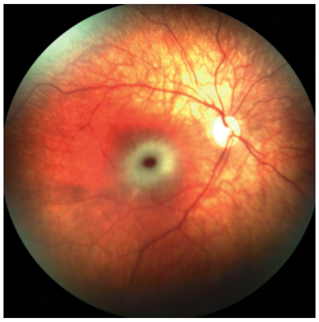

A. Myotonic dystrophy
B. Pompe disease
C. Prader-Willi syndrome
D. Spinal muscular atrophy
E. Tay-Sachs disease

Correct Answer: Tay-Sachs disease
Explanation: The correct answer is Tay-Sachs disease. Tay-Sachs disease is an autosomal recessive lysosomal storage disorder that results in progressive neurodegeneration. The cherry-red spot of the macula is a characteristic finding of this disease, caused by abnormally whitened, lipid-laden neurons surrounding and accentuating the color of the normal fovea.

GPT-4V Answer

**Image Comprhension:** The image provided appears to be a fundoscopic (retinal) examination of an eye. There is a cherry-red spot at the macula (central region of the retina) which is surrounded by a pale, whitish area. The surrounding retina displays the typical blood vessels branching out.	**Annotation:** Correct<br>**Comment:** None
**Recall of Medical Knowledge:** A cherry-red spot at the macula is a characteristic finding seen in a few metabolic and genetic disorders, including Tay-Sachs disease. Tay-Sachs disease is a rare inherited disorder that progressively destroys nerve cells (neurons) in the brain and spinal cord. It results from the absence of a specific enzyme called hexosaminidase-A. Macrocephaly, developmental delay, and hypotonia are common findings in many neurodevelopmental disorders, but the combination of these with a cherry-red spot narrows down the differential. The other listed diseases (Myotonic dystrophy, Pompe disease, Prader-Willi syndrome, Spinal muscular atrophy) have characteristic clinical, genetic, and radiologic features, but a cherry-red spot is not typical for them.	**Annotation:** Correct<br>**Comment:** None
**Step-by-step Reasoning:** Given the described clinical presentation and the fundoscopic findings: The key finding in the image is the cherry-red spot at the macula. Among the choices provided, Tay-Sachs disease is the one associated with a cherry-red spot. The presence of macrocephaly, developmental delay, hypotonia, and findings from the MRI of the brain further support the diagnosis of Tay-Sachs disease. Therefore, based on the information provided and the image's findings, the diagnosis for the 8-month-old girl would be Tay-Sachs disease.	**Annotation:** Correct<br>**Comment:** None
**Final Answer:** Answer: E.Tay-Sachs disease.	**Annotation:** Correct

Q69. NEJM Image Challenge 6/16/22

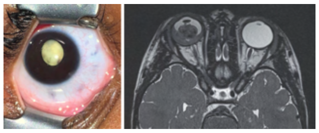

A 3-year-old girl was brought to the emergency department with a 2-month history of a white pupil and a 1-day history of redness and pain in the right eye. An eye examination showed leukocoria, as well as iris neovascularization and a white, nodular mass in the posterior chamber. The left eye was normal. B-scan ultrasonography showed calcification of the mass and vitreous seeding in the affected eye. Which of the following is the most likely diagnosis?

A. Cataract
B. Coats' disease
C. Ocular toxocariasis
D. Retinal detachment
E. Retinoblastoma

Correct Answer: Retinoblastoma
Explanation: A diagnosis of retinoblastoma was made. Magnetic resonance imaging of the head showed retinal and choroidal invasion without extraocular extension. Given the high-risk clinical features, the eye was enucleated the next day. Histopathological examination confirmed the diagnosis. Leukocoria requires urgent evaluation by an ophthalmologist in order to quickly identify life-threatening causes such as retinoblastoma.

GPT-4V Answer

**Image Comprhension:** The image contains two distinct visual representations. The first is a close-up image of an eye. The eye exhibits a white opacity or mass in the pupil area, referred to as leukocoria. Surrounding tissues, including the sclera, appear reddened or inflamed. The second part of the image appears to be an MRI or another type of radiological scan, showing cross-sectional images of the brain and potentially the orbits of the eyes. However, specific abnormalities in this part are not overtly discernible from the image alone.	**Annotation:** Incorrect<br>**Comment:** The GPT explanation states, "Specific abnormalities in this part are not overtly discernible from the image alone." That is false - the MRI image clearly demonstrates an abnormal opacification in the right eye (the gray/dark signal where we would expect it to be white).
**Recall of Medical Knowledge:** Leukocoria: This term refers to an abnormal white reflection from the retina. Causes include retinoblastoma, cataract, persistent fetal vasculature, Coats' disease, and retinal detachment among others. Retinoblastoma: This is a malignant tumor of the retina, primarily affecting young children. Symptoms can include leukocoria, strabismus, and inflammation. On imaging, calcification of the tumor is often noted. Cataract: It refers to the clouding of the lens, which can result in leukocoria in children. However, it typically doesn't cause redness or pain. Coats' disease: This is a non-hereditary condition where blood vessels develop abnormally in the eyes. It can lead to leukocoria but typically lacks calcification. Ocular toxocariasis: Caused by an infection with the parasite Toxocara. Symptoms can be inflammation and leukocoria but without calcification. Retinal detachment: This refers to the separation of the retina from its attachments. It can result in leukocoria but lacks other symptoms like pain and redness in most cases. Iris neovascularization: Refers to the growth of new blood vessels on the iris. This can be a result of many conditions, including retinoblastoma.	**Annotation:** Partially correct<br>**Comment:** "Abnormal white reflection from the retina" is not quite precisely correct. It should be an "abnormal white light reflex seen in the pupil," which can be derived from abnormalities in the retina but can also be a result of abnormalities elsewhere in the eye as well.
**Step-by-step Reasoning:** The presence of leukocoria, iris neovascularization, a nodular mass in the posterior chamber, calcification of the mass on ultrasonography, and vitreous seeding are all indicative of retinoblastoma. While some of the other options can cause leukocoria, the combination of symptoms and findings, especially calcification, points toward retinoblastoma. Cataract can lead to leukocoria but doesn't typically present with pain, redness, or calcification. Coats' disease might present with leukocoria but lacks the calcification seen on imaging. Ocular toxocariasis can cause inflammation but typically doesn't have calcifications. Retinal detachment can lead to leukocoria, but the presence of pain, redness, and especially calcification doesn't align with this diagnosis.	**Annotation:** Correct<br>**Comment:** None
**Final Answer:** Answer: The most likely diagnosis is E. Retinoblastoma.	**Annotation:** Correct

Q70. NEJM Image Challenge 6/9/22

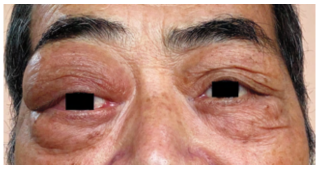

A 67-year-old man presented with a 1-year history of bilateral eye swelling and right eye protrusion and a 2-week history of blurry vision. There was no swelling of the parotid or submandibular glands. MRI of the orbits showed bilateral lacrimal gland and lateral rectus muscle swelling as well as mass lesions surrounding the optic nerves. He was initiated on daily prednisone with improvement in his symptoms. What is the diagnosis?

A. IgG4-related ophthalmic disease
B. Non-Hodgkin's lymphoma
C. Sjogren's syndrome
D. Tuberculosis-related dacryoadenitis
E. Thyroid eye disease

Correct Answer: IgG4-related ophthalmic disease
Explanation: The correct answer is IgG4-related ophthalmic disease. Serum IgG4 levels were elevated and biopsy specimen of the right lacrimal gland showed lymphoplasmacytic infiltrate with IgG4-positive plasma cells.

GPT-4V Answer

**Image Comprhension:** The image depicts the close-up view of an elderly man's face, particularly focusing on the region around the eyes. The individual displays bilateral eye swelling, with the right eye appearing more prominently protruded than the left. The eyes appear reddish with some fluid accumulation. The skin around the eyes is characterized by age-related changes such as wrinkles and some pigmentation. Black bars have been placed over the eyes, presumably to protect the patient's privacy.	**Annotation:** Correct<br>**Comment:** None
**Recall of Medical Knowledge:** IgG4-related ophthalmic disease is an immune-mediated condition that can lead to enlargement of the lacrimal glands and other tissues in the orbit. Symptoms can include eyelid swelling, double vision, and vision loss. MRI can show lacrimal gland enlargement. Non-Hodgkin's lymphoma can involve the orbit and manifest as mass lesions. Symptoms might vary but can include swelling and vision changes. However, bilateral involvement of specific structures like the lacrimal gland and muscles is less common. Sjogren's syndrome is an autoimmune disease that primarily affects the lacrimal and salivary glands leading to dry eyes and mouth. While it can cause eye symptoms, it typically does not lead to such prominent lacrimal gland and muscle involvement as seen on MRI. Tuberculosis-related dacryoadenitis is an inflammation of the lacrimal gland due to TB. It might be accompanied by systemic TB symptoms. The presentation on MRI would be distinct, and TB elsewhere in the body would likely be identified. Thyroid eye disease (TED), also known as Graves' ophthalmopathy, is an autoimmune condition where the eye muscles and fatty tissue behind the eye become inflamed. This can lead to proptosis (eye bulging), swelling, and vision changes. It's often associated with thyroid abnormalities.	**Annotation:** Correct<br>**Comment:** None
**Step-by-step Reasoning:** Given the information provided and the MRI findings:<br>The lack of parotid or submandibular gland swelling makes Sjogren's less likely. Tuberculosis-related dacryoadenitis would typically present with more systemic symptoms of TB. The bilateral involvement of specific structures like the lacrimal gland, lateral rectus muscle, and presence of mass lesions surrounding the optic nerves makes Non-Hodgkin's lymphoma less probable. Thyroid eye disease typically presents with more specific signs of thyroid dysfunction and does not usually involve the lacrimal gland in the manner described.	**Annotation:** Correct<br>**Comment:** None
**Final Answer:** Considering the bilateral lacrimal gland and lateral rectus muscle swelling on MRI, along with the patient's presentation and response to steroids, IgG4-related ophthalmic disease (Option A) seems the most likely diagnosis.	**Annotation:** Correct

Q71. NEJM Image Challenge 6/2/22

A 4-year-old boy was brought to the orthopedic clinic with a 2-day history of pain in the right hip and limping. There had been no preceding trauma or fever. Physical exam showed normal hip range of motion and an antalgic gait favoring the right side. A radiograph of the pelvis was performed. What is the most likely diagnosis?

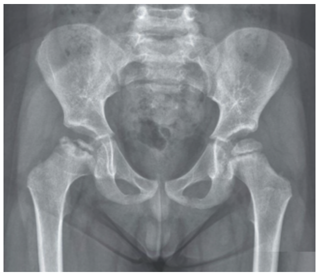

A. Legg–Calvé–Perthes disease
B. Juvenile idiopathic arthritis
C. Osteoid osteoma
D. Septic arthritis
E. Slipped capital femoral epiphysis

Correct Answer: Legg–Calvé–Perthes disease
Explanation: The correct answer is Legg–Calvé–Perthes disease. Treatment with short-term bracing was initiated to maintain hip abduction within a range of approximately 30 degrees to contain the femoral head within the acetabulum. The patient was also advised to avoid high-impact physical activity and was reassured with regard to an expected favorable diagnosis.

GPT-4V Answer

**Image Comprhension:** The provided image appears to be an X-ray radiograph of the pelvis, focusing on both hips. Upon examination, there's evidence of increased radiolucency in the right femoral head which suggests avascular necrosis. There's no obvious joint effusion, periarticular erosions, or abnormal bony growths. The left hip appears normal in this radiograph.	**Annotation:** Correct<br>**Comment:** None
**Recall of Medical Knowledge:** Legg–Calvé–Perthes disease (LCPD): A condition where there's a temporary loss of blood supply to the femoral head leading to avascular necrosis. It typically affects children between ages 4 to 8. Symptoms include pain and limping, which might be intermittent. Juvenile idiopathic arthritis (JIA): Chronic arthritis in children. Radiographically, it would show joint space narrowing, erosions, and in some cases joint effusion. Osteoid osteoma: A benign bone tumor that can cause pain. On radiographs, it presents as a radiolucent nidus with surrounding sclerosis, often in the diaphysis or metaphysis of long bones. Septic arthritis: Infection in the joint. In early stages, it may show only joint effusion, but as it progresses, can lead to rapid joint destruction. Slipped capital femoral epiphysis (SCFE): The femoral head slips off the neck along the growth plate. Common in adolescents and presents with pain and limping. Radiographically, the "ice cream" slips off the "cone".	**Annotation:** Correct<br>**Comment:** None
**Step-by-step Reasoning:** The image does not show joint effusion, erosions, or joint space narrowing, which rules out Septic arthritis and Juvenile idiopathic arthritis. There's no evident tumor-like lesion or surrounding sclerosis, ruling out Osteoid osteoma. The "ice cream" has not slipped off the "cone", making Slipped capital femoral epiphysis less likely. The primary finding is increased radiolucency in the right femoral head, indicative of avascular necrosis, which is characteristic of Legg–Calvé–Perthes disease.	**Annotation:** Correct<br>**Comment:** None
**Final Answer:** Based on the above, the most likely diagnosis for this 4-year-old boy is A.Legg–Calvé–Perthes disease.	**Annotation:** Correct

Q72. NEJM Image Challenge 5/26/22

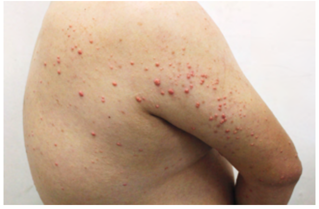

A 27-year-old man with a history of obesity presented to the dermatology clinic with an asymptomatic rash on his back, arms, and hands that had developed 1 week earlier. On physical examination, scattered pink-yellow papules were present on the upper back, extensor surfaces of the upper arms, and dorsa of the hands. A fasting blood sample was grossly lipemic. Which of the following is the most likely diagnosis?

A. Eruptive xanthomas
B. Generalized eruptive histiocytoma
C. Granuloma annulare
D. Molluscum contagiosum
E. Sebaceous hyperplasia

Correct Answer: Eruptive xanthomas
Explanation: A diagnosis of eruptive xanthomas due to severe hypertriglyceridemia was made. Eruptive xanthomas should be suspected in patients with risk factors suggestive of dyslipidemias. The skin lesions abated after treatment with fenofibrate, alcohol cessation, exercise, and a low-fat diet

---

GPT-4V Answer

**Image Comprhension:** The image displays a close-up of a skin surface, presumably the back or arm. The skin exhibits multiple scattered pink-yellow papules. These papules are well-circumscribed, raised lesions that appear consistent with the description given in the clinical scenario.	**Annotation:** Correct<br>**Comment:** None
**Recall of Medical Knowledge:** Eruptive xanthomas: These are lesions that are caused by the accumulation of cholesterol-rich lipoprotein within macrophages in the skin, resulting in the formation of foam cells. They are typically associated with high levels of triglycerides in the blood and can manifest as pink-yellow papules. They are common in conditions with hyperlipidemia. Generalized eruptive histiocytoma: A rare cutaneous disorder characterized by the sudden onset of widespread skin lesions. Granuloma annulare: This presents as annular (ring-shaped) lesions and is often more flesh-colored or reddish. Molluscum contagiosum: Caused by a viral infection, these lesions are usually pearly, dome-shaped papules with a central dimple. Sebaceous hyperplasia: This is the overgrowth of sebaceous glands leading to small, benign bumps on the skin, usually on the face. They are not related to lipid or cholesterol levels in the blood.	**Annotation:** Correct<br>**Comment:** None
**Step-by-step Reasoning:** The description and the image provided show pink-yellow papules which are consistent with eruptive xanthomas. The fasting blood sample was described as "grossly lipemic", indicating high levels of lipids in the blood. Eruptive xanthomas are associated with high triglyceride levels. The other options either do not match the clinical picture or the image (e.g., molluscum contagiosum would be expected to have a central dimple and granuloma annulare would present as annular lesions). Given the hyperlipidemia and the appearance of the rash, eruptive xanthomas are the most likely diagnosis.	**Annotation:** Correct<br>**Comment:** None
**Final Answer:** Answer: A. Eruptive xanthomas.	**Annotation:** Correct

Q73. NEJM Image Challenge 5/19/22

A 40-year-old man presented with a 2-week history of scaly foot rash, joint swelling, penile rash, knee swelling, tongue changes, red eyes, and low back pain. He had had diarrhea and urethral discharge 7 days before the onset of symptoms. On examination, yellow pustules with hyperkeratosis were seen on his plantar feet. What is the name of this physical examination sign?

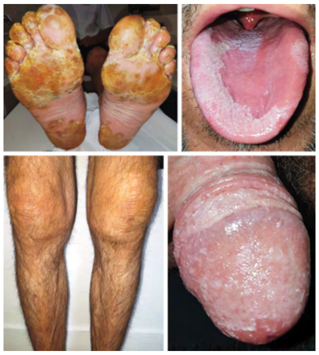

A. Keratoderma blenorrhagicum
B. Palmoplantar keratoderma
C. Pitted keratolysis
D. Porokeratosis
E. Pustular psoriasis

Correct Answer: Keratoderma blenorrhagicum
Explanation: The correct answer is keratoderma blenorrhagicum. The C-reactive protein was elevated, urinalysis showed sterile pyuria, and tests for infectious agents and rheumatoid factor were negative. Analysis of left knee synovial fluid was consistent with noninfectious inflammatory arthritis. A diagnosis of reactive arthritis was made. The patient was treated with nonsteroid anti-inflammatory agents, systemic glucocorticoids, and sulfasalazine.

GPT-4V Answer

Image Comprhension: The image shows several close-up views of different parts of a man's body.
The first view displays the soles of both feet with extensive yellow pustules and scaly hyperkeratosis. The pustules are more profound and concentrated on pressure points of the feet. The second view exhibits a tongue that looks swollen with a smooth and glossy appearance, possibly suggesting glossitis. The third view shows the frontal aspect of both knees. The knees appear swollen and slightly erythematous. The fourth view gives a closer look at a rash on the penile shaft. The rash appears erythematous with some whitish scaling on its surface.

Annotation: Correct
Comment: None

Recall of Medical Knowledge: Keratoderma blenorrhagicum: It is a skin manifestation often associated with Reactive arthritis, a triad of conjunctivitis, urethritis, and arthritis. The skin lesions appear as brown, scaly patches, primarily on the soles and palms.
Palmoplantar keratoderma: It refers to a group of disorders characterized by thickening of the skin on the palms and soles.
Pitted keratolysis: It is a bacterial skin infection that affects the soles of the feet and, less commonly, the palms of the hands. It is characterized by tiny pits in the skin.
Porokeratosis: It is a skin condition that manifests as annular plaques with a distinctive raised border.
Pustular psoriasis: This is a type of psoriasis characterized by white pustules surrounded by red skin. It can be localized to certain areas, such as the hands and feet, or covering most of the body.

Annotation: Correct
Comment: None

Step-by-step Reasoning: Given the provided history and the image:
The foot rash with yellow pustules and hyperkeratosis is most consistent with Keratoderma blenorrhagicum. The rash's appearance fits the description, especially when considering its association with symptoms such as urethral discharge, joint swelling, and red eyes.
The presence of diarrhea and urethral discharge before the onset of these symptoms, along with the triad of conjunctivitis (red eyes), urethritis (urethral discharge), and arthritis (joint swelling), strongly suggests Reactive arthritis.
Out of the provided options, Keratoderma blenorrhagicum is the only one associated with Reactive arthritis.

Annotation: Correct
Comment: None

Final Answer: Therefore, based on the information given and the visual representation, the physical examination sign displayed in the image is A. Keratoderma blenorrhagicum.

Annotation: Correct

Q74. NEJM Image Challenge 5/12/22

A 73-year-old man presented with 1-month history of fatigue and diffuse bone pain. Laboratory studies showed pancytopenia and a whole-body MRI showed osteonecrosis of the right humerus and right femur. A bone marrow biopsy showed macrophages with a "wrinkled tissue paper" appearance in the cytoplasm. What is the diagnosis?

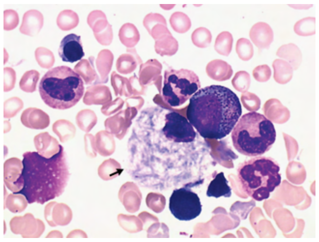

A. Chronic lymphocytic leukemia
B. Gaucher's disease
C. Mucopolysaccharidosis type
D. Niemann-Pick disease
E. Non-Hodgkin's lymphoma

Correct Answer: Gaucher's disease
Explanation: The correct answer is Gaucher's disease. In type 1 Gaucher's disease, some patients do not present with symptoms until late adulthood. Clinical features include fatigue, hepatosplenomegaly, pancytopenia, bone pain, and osteonecrosis. With enzyme-replacement therapy, the patient's symptoms abated and cell counts improved.

GPT-4V Answer	
**Image Comprhension:** The image provided appears to be a light microscopic view of a bone marrow smear. It displays a variety of cells, including some with what can be described as a "wrinkled tissue paper" appearance in the cytoplasm. These are likely macrophages. These macrophages exhibit a characteristic appearance where their cytoplasm contains crumpled or wrinkled structures. This appearance is distinct and not commonly seen in many hematologic or other disorders.	**Annotation:** Correct<br>**Comment:** None
**Recall of Medical Knowledge:** "Wrinkled tissue paper" appearance of the macrophages is a classical description associated with Gaucher cells. Gaucher's disease is a lysosomal storage disorder caused by a deficiency in the enzyme glucocerebrosidase. This deficiency leads to the accumulation of glucocerebroside in macrophages, which transform into Gaucher cells. Gaucher's disease is characterized by hepatosplenomegaly, bone involvement (pain, fractures, osteonecrosis), thrombocytopenia, and anemia. The other listed diagnoses: Chronic lymphocytic leukemia typically presents with lymphocytosis and has characteristic small, mature lymphocytes on smear. Mucopolysaccharidosis type refers to a group of metabolic disorders due to the absence or malfunctioning of lysosomal enzymes needed to break down molecules called glycosaminoglycans. Niemann-Pick disease, another lysosomal storage disease, affects the metabolism of lipid molecules and does not display the "wrinkled tissue paper" appearance. Non-Hodgkin's lymphoma would present with atypical lymphoid cells in the marrow, not macrophages with a "wrinkled tissue paper" appearance.	**Annotation:** Correct<br>**Comment:** None
**Step-by-step Reasoning:** Given the image and the description of the "wrinkled tissue paper" appearance of the macrophages, this is a classic characteristic of Gaucher cells. With the patient's presentation of fatigue, diffuse bone pain, pancytopenia, osteonecrosis, and the biopsy findings, the most consistent diagnosis is Gaucher's disease. Comparing this with other listed diagnoses, Gaucher's disease stands out due to its unique cellular appearance and clinical features.	**Annotation:** Correct<br>**Comment:** None
**Final Answer:** Based on the information and reasoning provided, the correct answer is B. Gaucher's disease.	**Annotation:** Correct

Q75. NEJM Image Challenge 5/5/22

A 74-year-old man with coronary artery disease presented to the emergency department with 3 days of anorexia and weakness. Computed tomography of the chest was performed, and blood cultures were drawn. What is the most likely diagnosis?

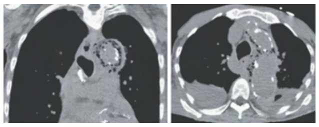

A. Emphysematous aortitis due to Clostridium septicum
B. Granulomatosis with polyangiitis
C. Pyogenic aortitis due to Salmonella enteritidis
D. Syphilitic aortitis due to Treponema pallidum
E. Tuberculous aortitis

Correct Answer: Emphysematous aortitis due to Clostridium septicum
Explanation: The correct answer is emphysematous aortitis due to Clostridium septicum. Abdominal imaging showed no overt signs of cancer. Intravenous penicillin G and clindamycin were administered. He was evaluated for surgery but the level of risk was determined to be unacceptable, and he died on hospital day 7 while receiving comfort care.

GPT-4V Answer

Image Comprhension: The images presented appear to be cross-sectional CT scans of the thorax, possibly in the region of the aorta. There are some abnormal findings:
There are large areas of low attenuation (dark areas) within the walls of the aorta. The structure of the aorta appears disrupted, with irregular thickening and luminal irregularities. The dark areas within the aortic wall suggest possible presence of gas or air.

Annotation: Correct
Comment: All correct

Recall of Medical Knowledge: Emphysematous aortitis due to Clostridium septicum: This is a rare but severe condition where Clostridium septicum bacteria infect the aorta. One of the defining features of this condition on imaging is the presence of gas or air within the aortic wall, which would present as dark areas on a CT scan.
Granulomatosis with polyangiitis: Previously known as Wegener's granulomatosis, this is a form of vasculitis that affects both small and large vessels. It can involve the aorta, but it doesn't typically cause gas formation.
Pyogenic aortitis due to Salmonella enteritidis: Salmonella is a known cause of infectious aortitis, but it doesn't typically cause gas formation within the aortic wall.
Syphilitic aortitis due to Treponema pallidum: Syphilis can cause aortitis during its tertiary stage. This would usually manifest as wall thickening or aneurysm formation, but not gas formation.
Tuberculous aortitis: Tuberculosis can infect the aorta, but like syphilis, it would cause wall thickening and not gas formation.

Annotation: Correct
Comment: All correct

Step-by-step Reasoning: Given the images and the presence of dark areas suggesting gas within the aortic wall:
The presence of gas within the aorta's wall is highly suggestive of emphysematous aortitis. The most common organism associated with emphysematous aortitis is Clostridium septicum. None of the other options listed are typically associated with gas formation within the aortic wall.

Annotation: Correct
Comment: All correct

Final Answer: Based on the image findings and the recalled medical knowledge, the most likely diagnosis is:
A. Emphysematous aortitis due to Clostridium septicum.

Annotation: Correct

Q76. NEJM Image Challenge 4/28/22

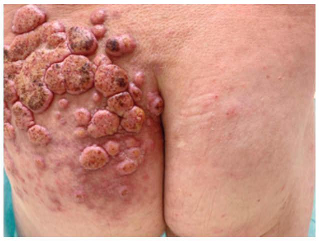

A 53-year-old landscaper presented to the dermatology clinic with a 4-month history of red, raised, itchy skin lesions on his left lower back and buttock. Physical examination showed numerous verrucous nodules and plaques with overlying crusting and surrounding erythema on the left lower back and buttock. Grocott-Gomori methenamine silver staining showed broad-based budding organisms. Chest imaging showed no abnormalities. Which of the following is the best treatment?

A. Clarithromycin and amikacin
B. Itraconazole
C. Penicillin
D. Rifampicin, isoniazid, pyrazinamide, and ethambutol
E. Trimethoprim-sulfamethoxazole

Correct Answer: Itraconazole
Explanation: A diagnosis of primary cutaneous blastomycosis was made. The result of a urine antigen test for blastomyces was also positive. Primary cutaneous blastomycosis is caused by blastomyces species, dimorphic fungi found in the eastern half of the United States that typically grow in soil and detritus from wooded areas. Cutaneous manifestations usually indicate disseminated disease but rarely occur by means of primary inoculation, as in this case. Itraconazole therapy was prescribed, and after 6 months the skin lesions had resolved.

GPT-4V Answer

**Image Comprhension:** The image depicts a close-up view of the skin on what appears to be the lower back and buttock area. There are numerous verrucous (wart-like) nodules and plaques present on the skin. These skin lesions are red and raised, and there is overlying crusting. Surrounding the nodules and plaques is a noticeable area of erythema (redness). The overall appearance suggests a significant dermatological condition that is likely chronic and inflammatory.	**Annotation:** Correct<br>**Comment:** None
**Recall of Medical Knowledge:** Grocott-Gomori methenamine silver staining is a special fungal stain that is used to identify fungi in tissue samples. The presence of broad-based budding organisms suggests a fungal infection. The clinical presentation along with the positive fungal stain suggests the possibility of chromoblastomycosis, a chronic fungal infection of the skin and subcutaneous tissue. This condition is caused by various pigmented fungi found in soil and plant material. It is commonly seen in individuals who have frequent contact with soil and plants, such as landscapers or farmers. Chromoblastomycosis typically presents as verrucous nodules and plaques and can sometimes resemble other dermatological conditions such as squamous cell carcinoma or psoriasis. Itraconazole is an antifungal medication that is frequently used as the first-line treatment for chromoblastomycosis. Other treatments can include surgical excision, cryotherapy, and other antifungal agents.	**Annotation:** Correct<br>**Comment:** None
**Step-by-step Reasoning:** The patient's presentation and occupation (landscaper) make him susceptible to fungal infections from soil and plant material. The appearance of the skin lesions, combined with the positive Grocott-Gomori methenamine silver staining showing broad-based budding organisms, is consistent with chromoblastomycosis. Out of the given treatment options, itraconazole is the first-line antifungal treatment for chromoblastomycosis. Thus, the best treatment option for this patient, based on the provided information, is itraconazole.	**Annotation:** Partially correct<br>**Comment:** Correct diagnosis is blastomycosis infection.
**Final Answer:** Answer: B. Itraconazole.	**Annotation:** Correct

Q77. NEJM Image Challenge 4/21/22

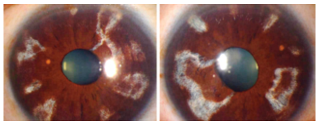

A 56-year-old man with sickle hemoglobin C disease and proliferative sickle cell retinopathy presented for a routine eye examination. He was asymptomatic. What is seen on this examination of the anterior segment of the eye?

A. Bilateral acute depigmentation of the iris (BADI)
B. Cornea verticillata
C. Iris atrophy
D. Mutton-fat keratic precipitates
E. Sunflower cataract

Correct Answer: Iris atrophy
Explanation: Extensive iris atrophy was noted in the anterior segments of both eyes. Iris atrophy is thought to be related to vaso-occlusion with infarction, and this finding on examination is closely associated with proliferative sickle cell retinopathy in the same eye. At a 6-month follow-up visit, the patient remained asymptomatic.

GPT-4V Answer

**Image Comprhension:** The image displays close-up views of two eyes, showing the anterior segment, specifically focusing on the iris. Both irises have a brownish color, with multiple irregular patches of lighter, depigmented areas scattered throughout, giving a patchy appearance. The structure of the iris seems to be intact with no obvious growths, nodules, or opacities.	**Annotation:** Correct<br>**Comment:** None
**Recall of Medical Knowledge:** Bilateral acute depigmentation of the iris (BADI): This refers to the bilateral and sudden loss of iris pigmentation. Patients often present with redness, discomfort, and photophobia, but the anterior chamber remains quiet without inflammation.<br>Cornea verticillata: Refers to a vortex-shaped pattern of fine white or gray opacities in the cornea, typically seen in Fabry disease or as a drug-induced change.<br>Iris atrophy: Iris atrophy involves the thinning of the iris, which can lead to the appearance of holes or defects in the iris. It can be a result of aging, trauma, or certain inflammatory eye conditions.<br>Mutton-fat keratic precipitates: These are large, greasy-appearing deposits on the corneal endothelium. They are typically seen in uveitis, especially granulomatous uveitis.<br>Sunflower cataract: This type of cataract displays a central disc of brown or yellowish-brown opacities. It's associated with Wilson's disease, a rare inherited disorder causing copper to accumulate in vital organs.	**Annotation:** Correct<br>**Comment:** None
**Step-by-step Reasoning:** The image does not show the cornea, so Cornea verticillata can be excluded. There is no presence of large, greasy-appearing deposits indicative of mutton-fat keratic precipitates, so this option can also be excluded. Sunflower cataract affects the lens, not the iris. Since the lens is not the focus here and no such cataract is visible, this option is ruled out. The iris does not seem to be thin with holes or defects, making Iris atrophy unlikely. The most consistent finding with the image is the scattered and patchy loss of pigmentation on the iris. This matches the description of Bilateral acute depigmentation of the iris (BADI).	**Annotation:** Incorrect<br>**Comment:** Technically the cornea is shown in the picture, just not in cross-section. Cornea verticillata are excluded not because the cornea isn't shown, but rather because the verticillata are not present. Also, at the end the reasoning specifically states that there is "scattered and patchy loss of pigmentation on the iris" which is consistent with iris atrophy, but the GPT incorrectly reasons that this is BADI.
**Final Answer:** Based on the above reasoning, the answer is: A. Bilateral acute depigmentation of the iris (BADI).	**Annotation:** Incorrect

Q78. NEJM Image Challenge 4/14/22

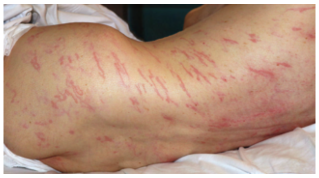

A 64-year-old man with metastatic lung adenocarcinoma who had recently started high-dose steroids for malignant spinal cord compression developed an itchy rash and diarrhea. He had a 3-year history of intermittent peripheral eosinophilia of unknown cause. What is the most likely diagnosis?

A. Cutaneous larva migrans
B. Cutaneous schistosomiasis
C. Larva currens from Strongyloides
D. Lichen striatus
E. Scabies

Correct Answer: Larva currens from Strongyloides
Explanation: Rhabditiform and filariform larvae of the species Strongyloides stercoralis were seen on stool examination, and he was diagnosed with strongyloides hyperinfection syndrome with larva currens. After treatment with oral ivermectin, the patient's rash and diarrhea abated.

GPT-4V Answer

**Image Comprhension:** The image displays a close-up view of a person's skin, showing a rash characterized by red, linear, serpiginous (snake-like) tracks. These tracks appear to be raised and are irregularly distributed across the skin. No other context or identifiable features are provided in the image.	**Annotation:** Correct<br>**Comment:** None
**Recall of Medical Knowledge:** Cutaneous larva migrans: This is a skin disease in humans, caused by the larvae of various nematode parasites. It presents as a red, itchy rash caused by the larvae moving under the skin. The most common cause is the dog and cat hookworm. The rash is usually self-limiting, resolving in weeks to months.<br>Cutaneous schistosomiasis: Caused by the cercarial form of schistosoma parasites. It results in a rash or itchy skin. It is known as "swimmer's itch" when seen in non-endemic areas.<br>Larva currens from Strongyloides: Strongyloides stercoralis can cause a unique form of cutaneous larva migrans known as larva currens. It is characterized by rapidly migrating, serpiginous urticarial tracks. It can be associated with eosinophilia and is seen in immunocompromised patients, especially those on steroids.<br>Lichen striatus: A benign skin condition that appears as a line of grouped, pink, raised spots that can be located anywhere on the body. The cause is unknown.<br>Scabies: Caused by the Sarcoptes scabiei mite. It typically presents as an intensely itchy rash with burrows, especially in web spaces of hands and feet.	**Annotation:** Correct<br>**Comment:** None
**Step-by-step Reasoning:** Given the serpiginous, raised rash seen in the image and the provided history:<br>The patient is immunocompromised due to steroids and has peripheral eosinophilia. The rash resembles the serpiginous tracks associated with larva currens from Strongyloides stercoralis. Of the choices given, Strongyloides is also known to be associated with eosinophilia. The other options are less consistent with the presentation. For instance, cutaneous larva migrans would move more slowly and wouldn't match the rapid migration of larva currens. Scabies usually presents with burrows, which aren't visible in the image. Lichen striatus and cutaneous schistosomiasis don't fit the clinical picture as well.	**Annotation:** Correct<br>**Comment:** None
**Final Answer:** Based on the above reasoning, the most likely diagnosis is: C. Larva currens from Strongyloides.	**Annotation:** Correct

Q79. NEJM Image Challenge 4/7/22

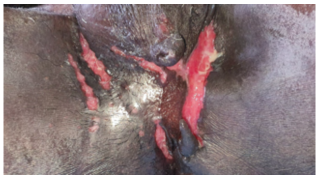

A 30-year-old man with Crohn's disease presented to the dermatology clinic with multiple painful ulcers in his groin and perineal area, as well as an ongoing Crohn's disease flare with fatigue, abdominal pain, and bloody diarrhea. Physical examination of the skin showed deep, linear erosions across the perineum and inguinal folds, on the scrotum and penile shaft, and in the intergluteal cleft. Which of the following is the most likely etiology underlying the genital ulcers?

A. Ecthyma gangrenosum
B. Knife-cut ulcers from Crohn's disease
C. Lichen sclerosus
D. Linear IgA disease
E. Pemphigus foliaceus

Correct Answer: Knife-cut ulcers from Crohn's disease
Explanation: Knife-cut ulcers are a cutaneous manifestation of Crohn's disease in which non-caseating, granulomatous inflammation is observed on skin biopsy that is similar to that observed on gastrointestinal biopsy.

GPT-4V Answer

Image Comprhension: The image presents a close-up view of what appears to be a person's groin and perineal area. There are several deep, linear ulcers and erosions that can be seen across the surface. The skin around these ulcers appears inflamed and slightly swollen. These erosions extend across different parts of the groin, including the scrotum, penile shaft, and possibly the intergluteal cleft.

Annotation: Correct
Comment: None

Recall of Medical Knowledge: Ecthyma gangrenosum: This is a skin manifestation that results from an underlying infection, commonly Pseudomonas aeruginosa. It usually starts as a small red macule which quickly becomes a vesicle, then a hemorrhagic bulla, and finally a necrotic ulcer with a surrounding erythematous halo. It is typically associated with sepsis.
Knife-cut ulcers from Crohn's disease: Crohn's disease is an inflammatory bowel disease that can affect any part of the gastrointestinal tract. Extra-intestinal manifestations can also occur, including skin ulcers, especially in the perineal area. These ulcers are called "knife-cut" because of their deep, linear appearance.
Lichen sclerosus: A chronic inflammatory skin condition that often affects the genital and perianal areas. It usually presents as white, thin, wrinkled patches of skin that can tear easily.
Linear IgA disease: An autoimmune blistering disorder where the person produces antibodies against their own skin leading to the formation of blisters. It's called "linear" because of the line of IgA deposits found at the dermal-epidermal junction on immunofluorescence.
Pemphigus foliaceus: Another autoimmune blistering disorder, but in this condition, the antibodies target the upper layer of the skin. It usually presents as superficial blisters that rupture easily, leading to crusting erosions.

Annotation: Correct
Comment: None

Step-by-step Reasoning: The patient has Crohn's disease and is experiencing a flare-up with systemic symptoms. The description of the ulcers as "deep, linear erosions" across multiple areas including the perineum, inguinal folds, scrotum, penile shaft, and intergluteal cleft matches the characteristic appearance of knife-cut ulcers seen in Crohn's disease. Ecthyma gangrenosum is often associated with sepsis and would typically not be isolated to the genital region. Lichen sclerosus presents as thin white patches, which is not consistent with the image provided. Both Linear IgA disease and Pemphigus foliaceus present as blisters, which is different from the deep ulcers shown in the image.

Annotation: Correct
Comment: None

Final Answer: Given the clinical history and the image provided, the most likely etiology underlying the genital ulcers in this patient is: B. Knife-cut ulcers from Crohn's disease.

Annotation: Correct

Q80. NEJM Image Challenge 3/31/22
A 53-year-old man presented with several hours of right eye itching after gardening near a horse and sheep farm. A physical exam was performed. What is the most likely diagnosis?

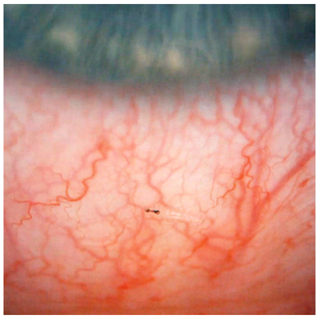

A. Allergic conjunctivitis
B. Anterior uveitis
C. Bacterial conjunctivitis
D. Ophthalmomyiasis
E. Superficial punctate keratitis

Correct Answer: Allergic conjunctivitis
Explanation: The answer is ophthalmomyiasis. More than a dozen mobile, translucent larvae were observed on the cornea, bulbar conjunctiva, and upper and lower conjunctival fornices. The patient underwent mechanical removal of the organisms with forceps with improvement in his symptoms.

GPT-4V Answer	
**Image Comprhension:** The image depicts a close-up view of what appears to be the conjunctiva of an eye. It shows an intricate network of blood vessels with a pale background, indicating inflammation or irritation. Moreover, there seems to be a tiny, elongated foreign body (potentially an insect larva) attached to the conjunctiva.	**Annotation:** Correct<br>**Comment:** None
**Recall of Medical Knowledge:** Allergic conjunctivitis: Often presents with itching, redness, and tearing, typically in both eyes. It can be triggered by allergens like pollen, animal dander, or dust mites. Anterior uveitis: Inflammation of the uvea in the eye, leading to eye redness, pain, light sensitivity, and blurred vision. Usually affects one eye. Bacterial conjunctivitis: Presents with a red eye, thick yellow discharge, and may involve one or both eyes. Itching is not a predominant symptom. Ophthalmomyiasis: Infestation of the eye by fly larvae, commonly associated with exposure to livestock or unsanitary conditions. Can lead to redness, itching, and foreign body sensation. Superficial punctate keratitis: Inflammation of the corneal surface, which can cause symptoms like tearing, redness, and light sensitivity.	**Annotation:** Partially correct<br>**Comment:** Anterior uveitis can often be bilateral and not just unilateral ("usually affects one eye" in the GPT explanation.
**Step-by-step Reasoning:** Given the image and the accompanying history:<br>The image doesn't show the bilateral redness and discharge typical of bacterial or allergic conjunctivitis. The absence of corneal involvement makes superficial punctate keratitis less likely. Anterior uveitis usually presents with pain and light sensitivity, which aren't mentioned in the case. The presence of a likely larva and the history of gardening near a farm make ophthalmomyiasis a strong contender.	**Annotation:** Partially correct<br>**Comment:** The image does show redness and injection, so this could still potentially be bacterial or allergic conjunctivitis. Also, you can't definitely comment on corneal involvement or not, since the cornea is not pictured in the image. The main element of the reasoning here is not the LACK of other things, but rather the PRESENCE of the main finding, which is the larvae on the conjunctiva.
**Final Answer:** Considering the provided options and correlating with the image and history, the most likely diagnosis is: D. Ophthalmomyiasis.	**Annotation:** Correct

Q81. NEJM Image Challenge 3/24/22

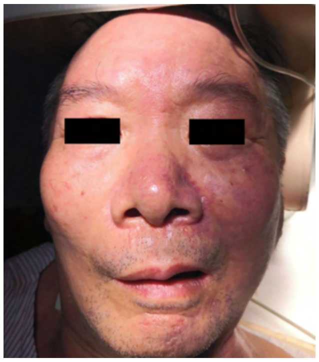

A 63-year-old man developed purplish discoloration of his face after he underwent stenting and balloon dilation of the left common carotid artery. Cholesterol embolization syndrome was diagnosed. What is the name of this physical exam finding?

A. Cutis marmorata
B. Harlequin syndrome
C. Livedo racemosa
D. Livedo reticularis
E. Telangiectasia

Correct Answer: Livedo reticularis
Explanation: The correct answer is livedo reticularis. He was stabilized in the ICU, and the livedo reticularis resolved 1 week after the event.

GPT-4V Answer

**Image Comprhension:** The image displays a close-up view of a man's face with notable features. The man's eyes are blacked out, possibly to protect his privacy. His facial skin displays areas of purplish discoloration, particularly noticeable on the cheeks and near the nose. There are some scattered red markings on the skin which appear like minor abrasions or small contusions.	**Annotation:** Partially correct<br>**Comment:** The red markings represent cherry angiomas and not abrasions
**Recall of Medical Knowledge:** Cholesterol embolization syndrome occurs when cholesterol plaques get dislodged, typically from the aorta or other major arteries, and travel to smaller vessels where they can cause occlusion. When this occurs, there can be ischemic damage to the skin and other organs. One of the cutaneous manifestations of cholesterol embolization syndrome is Livedo racemosa.<br>Cutis marmorata is a transient mottled appearance of the skin and is typically seen in infants. Harlequin syndrome is characterized by unilateral facial flushing and sweating. Livedo racemosa is a pattern of the skin showing reddish-blue discoloration, irregular, broken, branched, and non-continuous. Livedo reticularis is a net-like pattern of reddish-blue skin discoloration. Telangiectasia refers to small dilated blood vessels on the skin.	**Annotation:** Incorrect<br>**Comment:** None
**Step-by-step Reasoning:** Given the description and the image:<br>The skin discoloration does not appear mottled as in cutis marmorata, so option A is ruled out. There is no unilateral facial flushing or sweating indicative of Harlequin syndrome, ruling out option B. The discoloration pattern is irregular, broken, branched, and noncontinuous, which matches the description of Livedo racemosa, making option C a strong candidate. The pattern does not appear as a simple net-like pattern that would be typical of livedo reticularis, so option D is less likely. There are no small dilated blood vessels on the skin, ruling out option E. Based on the image comprehension and the recall of medical knowledge, the most	**Annotation:** Correct<br>**Comment:** Both GPT and I prefer Livedo racemosa to livedo reticularis to describe cutaneous findings here
**Final Answer:** Based on the image comprehension and the recall of medical knowledge, the most likely physical exam finding in this patient with a history of cholesterol embolization syndrome is C. Livedo racemosa.	**Annotation:** Incorrect

Q82. NEJM Image Challenge 3/17/22

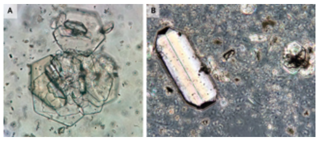

A 37-year-old man presented early in life with nephrolithiasis and pyelonephritis. Two weeks before this encounter, he was treated for a urinary tract infection. Microscopic examination of a spun urine specimen is shown, demonstrating two crystal types. What are these crystals, demonstrated in panels A and B, respectively?

A. Calcium oxalate and triple phosphate (struvite) crystals
B. Cholesterol and acyclovir crystals
C. Cystine and triple phosphate (struvite) crystals
D. Sulfonamide and cystine crystals
E. Uric acid and calcium oxalate crystals

Correct Answer: Cystine and triple phosphate (struvite) crystals
Explanation: The correct answer is cystine and triple phosphate (struvite) crystals in panels A and B, respectively. In this patient, the hexagonal cystine crystals were seen concomitantly with the rhombohedral triple phosphate, or struvite, crystals. The patient had received a diagnosis of cystinuria at the age of 1 and was treated with urinary alkalinization, high fluid intake, and tiopronin. Though the causative organism of his recently treated urinary tract infection is unknown, triple phosphate crystals are associated with urease-producing organisms.

GPT-4V Answer

**Image Comprhension:** The provided images showcase two distinct types of urinary crystals under microscopic examination.<br>Panel A displays irregular, envelope-like, and somewhat star-shaped crystals. They seem to have distinct edges, and the overall form resembles angular geometric shapes.<br>Panel B shows a larger, coffin-shaped crystal which is surrounded by a background of other smaller crystals and cellular debris. The larger crystal's shape is elongated with somewhat parallel sides.	**Annotation:** Partially correct<br>**Comment:** Error: Envelope-like is a classic description for different type of stone - calcium oxalate stone. Cystine stone is classically described as hexagon shaped, and they can be overlaid on top of each other.
**Recall of Medical Knowledge:** Calcium oxalate crystals are the most common type of crystals seen in urine. They have two main forms:<br>Dihydrate: which is envelope or wedge-shaped Monohydrate: which can appear dumbbell-shaped or like elongated ovals Triple phosphate (struvite) crystals are often seen in alkaline urine and are associated with urinary tract infections, especially with urease-producing bacteria. They often have a characteristic "coffin-lid" appearance.<br>Cystine crystals have a characteristic hexagonal shape and are seen in conditions like cystinuria, a genetic disorder that results in increased urinary cystine levels.<br>Cholesterol crystals are not common in urine but when present, they can have a rectangular or notched appearance.<br>Acyclovir crystals are associated with acyclovir therapy and have a variety of shapes, but they don't match the given images.<br>Sulfonamide crystals are associated with sulfonamide medications and can have a variety of shapes.<br>Uric acid crystals can appear as rhomboid or rosettes and are more frequently seen in acidic urine.	**Annotation:** Correct<br>**Comment:** None
**Step-by-step Reasoning:** Panel A's crystals have the distinctive envelope-like appearance, which matches the description of calcium oxalate dihydrate crystals.<br>Panel B's coffin-shaped crystal matches the description of triple phosphate (struvite) crystals.	**Annotation:** Partially correct<br>**Comment:** Error: wrong picture analysis, and therefore wrong logic was used to pick the answer
**Final Answer:** Given these observations, the answer to the question is: A. Calcium oxalate and triple phosphate (struvite) crystals.	**Annotation:** Incorrect

Q83. NEJM Image Challenge 3/10/22

A 42-year-old man with human immunodeficiency virus (HIV) infection presented with painful skin ulcerations and systemic symptoms. Physical exam showed large ulcers with overlying keratosis and crusting scattered across his scalp, face, perineum, and limbs. Laboratory studies showed a CD4 count of 399 cells/mm3. Skin biopsy showed superficial dermal infiltration of lymphocytes, histiocytes, and plasma cells in a lichenoid pattern with psoriasiform epidermal hyperplasia. What is the most likely diagnosis?

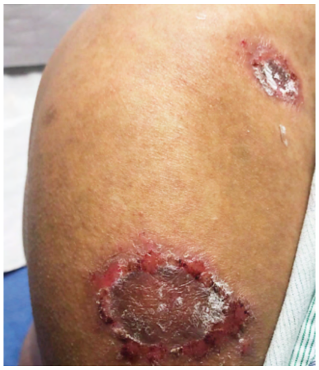

A. Bacillary angiomatosis
B. Disseminated histoplasmosis
C. Lupus vulgaris (tuberculosis luposa)
D. Malignant syphilis
E. Norwegian scabies

Correct Answer: Malignant syphilis
Explanation: Immunohistochemical analysis revealed the presence of spirochetes. A rapid plasmin reagin titer was 1:512. The patient received a diagnosis of malignant syphilis, an uncommon form of secondary syphilis characterized by systemic symptoms and a papulopustular eruption that evolves into painful, necrotic ulcers. The patient's symptoms were reduced with the administration of intravenous penicillin G.

GPT-4V Answer

**Image Comprhension:** The image shows a section of skin, presumably from the patient's limb, which has multiple, irregularly shaped, well-defined skin ulcerations. These ulcers exhibit crusting, overlying keratosis, and reddish discoloration. The surrounding skin looks relatively normal, but the specific regions with the ulcers appear inflamed and possibly infected.	**Annotation:** Partially correct<br>**Comment:** Would not described these lesions as ulcers but rather scaly plaques
**Recall of Medical Knowledge:** Bacillary angiomatosis: This is a vascular proliferative disease caused by Bartonella species. It is commonly found in patients with AIDS and presents as reddish-purple papules that may become nodular or ulcerative. Disseminated histoplasmosis: This is a fungal infection caused by Histoplasma capsulatum. It commonly presents as systemic symptoms with lung involvement, but can lead to cutaneous manifestations in the form of papules, nodules, or ulcers. Lupus vulgaris (tuberculosis luposa): This is a form of cutaneous tuberculosis. It presents as reddish-brown nodules which gradually enlarge and form plaques. Malignant syphilis: Also known as lues maligna, this is a rare form of secondary syphilis. It presents as painful skin ulcerations, which can be accompanied by systemic symptoms in patients with HIV/AIDS. Norwegian scabies: This is a severe form of scabies infestation, common in immunocompromised individuals. It presents as thick crusts and scales, often with underlying erythroderma.	**Annotation:** Correct<br>**Comment:** None
**Step-by-step Reasoning:** Given the presented details and the image:<br>The skin findings, combined with the biopsy findings of a lichenoid pattern and psoriasiform epidermal hyperplasia, do not typically align with the presentation of bacillary angiomatosis or disseminated histoplasmosis. Lupus vulgaris usually presents as plaques rather than the ulcerative lesions seen in the image. Norwegian scabies would typically manifest with more extensive erythroderma and thick crusting, which is not clearly visible in the image. Malignant syphilis presents with painful skin ulcerations and can manifest with systemic symptoms, especially in patients with HIV/AIDS, aligning well with the image and provided clinical details.	**Annotation:** Partially correct<br>**Comment:** The most important diagnostic clue in this case is from path: plasma infiltrates with lichenoid pattern, characteristic of syphilis infection. Lupus vulgaris would be a reasonable alternative based on clinical picture, but we usually see granulomatous inflammation on pathology.
**Final Answer:** Based on the above, the most likely diagnosis is: D. Malignant syphilis.	**Annotation:** Correct

Q84. NEJM Image Challenge 3/3/22

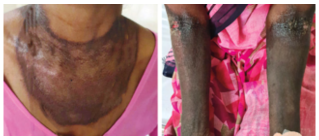

A 48-year-old woman who lived in rural India presented with diarrhea, weight loss, and a rash. Physical examination showed well-demarcated areas of hyperpigmentation on the sun-exposed areas of her neck, chest, and forearms. What is the most likely diagnosis?

A. Cobalamin (vitamin B12) deficiency
B. Pellagra
C. Phytophotodermatitis
D. Porphyria cutanea tarda
E. Zinc deficiency

Correct Answer: Pellagra
Explanation: A diagnosis of pellagra, or niacin deficiency, was made on the basis of the patient's diarrhea and symmetric, photosensitive dermatitis, including a typical Casal's necklace rash. After the patient was treated with vitamin B complex supplementation, the diarrhea resolved and the skin hyperpigmentation was reduced.

GPT-4V Answer	
**Image Comprhension:** The image displays a woman's neck, chest, and forearms. On the neck and chest, there are well-demarcated areas of hyperpigmentation. The pigmentation is more prominent on sun-exposed areas. The forearms, on the other hand, display a different pattern of skin changes. There is a visible wound on one forearm, surrounded by areas of dark hyperpigmentation. The skin abnormalities seem to be localized mainly to areas exposed to sunlight.	**Annotation:** Correct<br>**Comment:** None
**Recall of Medical Knowledge:** Cobalamin (vitamin B12) deficiency: This typically leads to megaloblastic anemia and neurologic manifestations such as numbness, tingling, and gait disturbances. Skin hyperpigmentation isn't a hallmark feature. Pellagra: Caused by a deficiency in niacin (vitamin B3). Its classical symptoms are diarrhea, dermatitis, and dementia. The dermatitis seen in pellagra often affects sun-exposed areas and is described as rough, scaly, and hyperpigmented. Phytophotodermatitis: This is a skin condition that occurs after contact with certain plants followed by exposure to sunlight. It leads to an irregular rash, blisters, and post-inflammatory hyperpigmentation. Porphyria cutanea tarda: A group of disorders resulting from a buildup of natural chemicals that produce porphyrin in the body. It can cause blistering, itching, and swelling of the skin after sun exposure. Zinc deficiency: Can lead to a characteristic rash known as acrodermatitis enteropathica, which often involves the face, hands, and feet. Diarrhea is also a common symptom.	**Annotation:** Correct<br>**Comment:** None
**Step-by-step Reasoning:** Cobalamin (vitamin B12) deficiency can be ruled out as the symptoms don't align with the image and the clinical presentation. Phytophotodermatitis typically presents with more of an acute blistering rash after contact with specific plants and subsequent sun exposure, so this is less likely. Porphyria cutanea tarda leads to blistering on sun-exposed areas, but the pattern of pigmentation and clinical presentation in the image isn't typical for this condition. Zinc deficiency can lead to a rash, but it typically affects the face, hands, and feet and has a different appearance. Pellagra fits the clinical picture the most. The patient has diarrhea and the skin findings of dermatitis on sun-exposed areas. Pellagra is common in populations where maize (corn) is a staple food and is not prepared in a way that allows for absorption of niacin.	**Annotation:** Correct<br>**Comment:** None
**Final Answer:** Answer: Based on the image and the provided clinical information, the most likely diagnosis is B.Pellagra.	**Annotation:** Correct

Q85. NEJM Image Challenge 2/24/22

A 31-year-old man presented with 10 years of progressive fingertip and toe enlargement and intermittent aches of his distal forearms and lower legs. Physical exam was notable for digital clubbing, proximal nail fold hyperpigmentation, and forearm and lower leg tenderness of palpation. Plain radiographs of the ankles showed increased periosteal bone formation at the distal tibia and fibula. Serum laboratory tests, echocardiogram, and a whole-body PET-CT were normal. What is the most likely diagnosis?

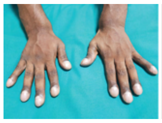

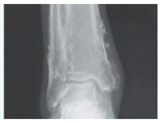

A. Acromegaly
B. Osteopetrosis
C. Paget's disease
D. Primary hypertrophic osteoarthropathy
E. Scleromyxedema

Correct Answer: Primary hypertrophic osteoarthropathy
Explanation: The patient was diagnosed with primary hypertrophic osteoarthropathy, a hereditary syndrome characterized by clubbing, periosteal bone proliferation, and (not seen in this case) facial skin thickening and hyperhidrosis. The patient was treated with antiinflammatories, and later zoledronic acid, with improvement in his pain.

GPT-4V Answer

**Image Comprhension:** The image provided consists of two parts. On the left, there is a clear photograph of a person's hands, which show thickened, bulbous fingertips indicating digital clubbing. The skin around the nails, especially the proximal nail folds, appears to be hyperpigmented. On the right, there's an X-ray radiograph of what appears to be an ankle. The X-ray shows irregular periosteal thickening of the distal ends of the tibia and fibula, a sign of increased periosteal bone formation.	**Annotation:** Correct<br>**Comment:** None
**Recall of Medical Knowledge:** Digital Clubbing: A thickening of the skin at the base of the fingernails or toenails, often associated with certain lung diseases, heart diseases, liver disease, and gastrointestinal conditions.<br>Acromegaly: Caused by excess growth hormone, it leads to enlarged hands, feet, and facial features, but not necessarily digital clubbing or periosteal bone formation.<br>Osteopetrosis: A rare inherited disorder that makes bones densify, becoming abnormally thick. It doesn't cause digital clubbing.<br>Paget's disease: A bone disorder where bones are broken down and rebuilt at an increased rate. It's localized and doesn't usually cause digital clubbing.<br>Primary hypertrophic osteoarthropathy (PHO): A rare genetic disorder characterized by digital clubbing, periosteal bone formation, and joint pain. Also known as pachydermoperiostosis.<br>Scleromyxedema: A rare skin disorder characterized by lumps, skin thickening, and hardening. It doesn't cause digital clubbing or periosteal bone formation.	**Annotation:** Correct<br>**Comment:** None
**Step-by-step Reasoning:** Starting with the image comprehension, we identify digital clubbing and periosteal bone formation, which are key clinical features. Given the patient's presentation and the features on the radiograph:<br>Acromegaly may present with enlarged hands but not with clubbing or periosteal bone formation.<br>Osteopetrosis leads to dense bones but not digital clubbing.<br>Paget's disease affects bone remodeling but does not cause digital clubbing.<br>Scleromyxedema affects the skin but not the bones in the described manner.<br>Thus, the only diagnosis that aligns with the patient's presentation of digital clubbing, periosteal bone formation, and the provided images is Primary hypertrophic osteoarthropathy (PHO).	**Annotation:** Correct<br>**Comment:** None
**Final Answer:** Answer: D.Primary hypertrophic osteoarthropathy.	**Annotation:** Correct

Q86. NEJM Image Challenge 2/17/22

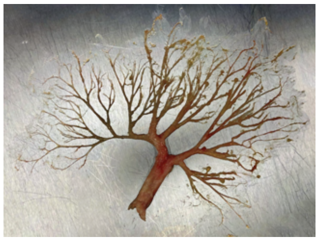

A 50-year-old man with a history of several months of productive cough presented to the emergency department with stupor and hypercarbic respiratory failure. He was intubated. Chest radiograph several days later revealed complete collapse of the left lung. On bronchoscopy, several casts, as shown, were retrieved. Follow-up CT of the chest showed only bibasilar atelectasis. What is the etiology of his casts?

A. Asthma
B. Bronchiectasis
C. Cystic fibrosis
D. Plastic bronchitis
E. Tuberculosis

Correct Answer: Plastic bronchitis
Explanation: The correct answer is plastic bronchitis, a rare condition that may be associated with thoracic-duct trauma, sickle cell disease, and other systemic conditions. Serial casts were removed via bronchoscopy, and he was initiated on treatment including bronchodilators, mucolytic agents, and aerosolized tissue plasminogen activator. He was ultimately extubated and referred for further diagnostic testing.

GPT-4V Answer

**Image Comprhension:** The image shows a structure that resembles the branching of bronchi and bronchioles. It appears to be a bronchial cast, which is a mucus or cellular material that has taken the shape of the bronchial tree. This cast was retrieved from the patient's airways during bronchoscopy.	**Annotation:** Correct<br>**Comment:** None
**Recall of Medical Knowledge:** Bronchial casts are typically associated with conditions where there's excessive production of mucus or inflammation leading to shedding of the bronchial lining. Here's a brief overview of the conditions mentioned:<br>A. Asthma: A chronic inflammatory disease of the airways that leads to intermittent bronchoconstriction. Patients often produce thick mucus, but bronchial casts are not a common feature. B. Bronchiectasis: A chronic condition characterized by irreversible dilatation of bronchi. Patients often produce copious amounts of purulent sputum, and occasionally, bronchial casts. C. Cystic fibrosis: A genetic disorder leading to thick mucus production throughout the body, including in the lungs. Patients often develop bronchiectasis and can produce bronchial casts, but it usually presents earlier in life. D. Plastic bronchitis: A rare condition where patients produce large, branching bronchial casts made of lymphatic or inflammatory material. These casts can cause bronchial obstruction, leading to atelectasis or lung collapse. E. Tuberculosis: A bacterial infection primarily affecting the lungs. It does not typically produce bronchial casts.	**Annotation:** Correct<br>**Comment:** None
**Step-by-step Reasoning:** The patient's age and presentation do not fit with Cystic fibrosis as it usually presents in younger patients. Tuberculosis is not commonly associated with bronchial casts, and there's no mention of classic symptoms such as night sweats, fever, and weight loss. Asthma can lead to mucus plugging, but complete lung collapse and the formation of casts as extensive as shown is uncommon. Bronchiectasis can produce mucus plugs and occasionally bronchial casts, but the clinical picture doesn't align well with this patient's acute presentation. Plastic bronchitis is consistent with the finding of large bronchial casts causing lung collapse. Additionally, the patient's acute presentation with respiratory failure and stupor suggests a significant airway obstruction, which fits well with the sudden occlusion that such casts can cause.	**Annotation:** Correct<br>**Comment:** None
**Final Answer:** The most likely etiology of the casts in this patient is D. Plastic bronchitis.	**Annotation:** Correct

Q87. NEJM Image Challenge 2/10/22

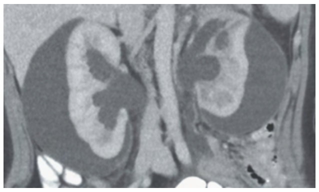

A 21-year-old woman with a history of surgically corrected Hirschsprung's disease presented with 6-week history of abdominal pain and constipation. Her blood pressure was 162/108 mm Hg. She had an elevated creatinine and CT showed large subcapsular urinomas and hydronephrosis caused by distal ureteral obstruction from a rectal mass. What is the likely mechanism of her hypertension?

A. Atherosclerotic renal artery stenosis
B. Fibromuscular dysplasia
C. Medullary sponge kidney
D. Page kidney
E. Polycystic kidney disease

Correct Answer: Page kidney
Explanation: The correct answer is Page kidney. Page kidney refers to the phenomenon whereby external compression of the renal parenchyma impedes renal blood flow, resulting in increased renin secretion and hypertension. The patient underwent percutaneous drainage of the urinomas with normalization of her blood pressure followed by ureteral stenting and biopsy of the rectal mass, which showed adenocarcinoma

---

GPT-4V Answer

**Image Comprhension:** The provided image appears to be a computed tomography (CT) scan of the abdominal area, focusing on the kidneys. Both kidneys seem enlarged and show evidence of hydronephrosis, indicated by the dilated renal pelvis and calyces. Additionally, there are darker, fluid-filled regions adjacent to the kidneys, which can be interpreted as subcapsular urinomas. The kidneys appear compressed, especially on the right side.	**Annotation:** Correct<br>**Comment:** None
**Recall of Medical Knowledge:** Hypertension Mechanisms in Renal Diseases: Hypertension can result from renal parenchymal disease and renal vascular disease. Obstructive uropathy can lead to hypertension due to fluid overload and activation of the renin-angiotensin-aldosterone system (RAAS). Specific Conditions: Atherosclerotic renal artery stenosis: It is a condition in which the renal arteries narrow due to atherosclerosis. It causes hypertension through ischemia and activation of RAAS. Fibromuscular dysplasia: A non-atherosclerotic disease causing renal artery stenosis. It leads to hypertension via similar mechanisms as atherosclerotic stenosis. Medullary sponge kidney: A congenital disorder characterized by cystic dilatation of the collecting tubules in one or both kidneys. It is not typically associated with hypertension. Page kidney: Refers to the external compression of a kidney, usually due to a hematoma or urinoma, which leads to ischemia and activation of RAAS, causing hypertension. Polycystic kidney disease: A genetic disorder causing multiple cysts in the kidneys, leading to decreased kidney function and hypertension.	**Annotation:** Correct<br>**Comment:** None
**Step-by-step Reasoning:** Given the CT scan and clinical history, there is evidence of hydronephrosis and subcapsular urinomas compressing the kidneys externally.<br>Among the choices provided:<br>Atherosclerotic renal artery stenosis and fibromuscular dysplasia lead to hypertension primarily through renal artery stenosis, which is not mentioned or depicted. Medullary sponge kidney, as recalled, is not typically associated with hypertension. Polycystic kidney disease results in multiple cysts throughout the kidneys, which doesn't match the imaging findings. Therefore, the most fitting explanation for the hypertension is Page kidney, where external compression (in this case, from the urinomas) leads to renal ischemia and subsequent activation of RAAS.	**Annotation:** Correct<br>**Comment:** None
**Final Answer:** Answer: D. Page kidney.	**Annotation:** Correct

Q88. NEJM Image Challenge 2/3/22

A 60-year-old man with HIV presented to the ophthalmology clinic with eyelid and facial lesions. His CD4 cell count was 20 per cubic millimeter and his HIV viral load was 120,000 copies/ml. Physical exam showed numerous dome-shaped papules with central umbilication. What is the most likely diagnosis?

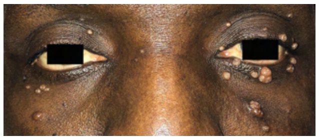

A. Basal-cell carcinoma
B. Cryptococcosis
C. Histoplasmosis
D. Molluscum contagiosum
E. Verruca vulgaris

Correct Answer: Molluscum contagiosum
Explanation: Incision and curettage of the lesions were performed, and histopathological assessment confirmed molluscum contagiosum. The patient was referred for HIV treatment, and at follow-up visit 6 months later, he had had no recurrence of the lesions.

GPT-4V Answer

**Image Comprhension:** The image showcases a close-up view of a person's face, focusing on the region surrounding the eyes. Both eyelids and parts of the face contain multiple, small, raised lesions. These lesions appear as dome-shaped papules, many of which have a central depression or umbilication. The eyes have been blacked out to preserve anonymity.	**Annotation:** Correct<br>**Comment:** None
**Recall of Medical Knowledge:** Basal-cell carcinoma is a type of skin cancer that usually appears as a bump or growth that often has a pearly appearance. It tends to grow slowly and rarely metastasizes. Cryptococcosis is a fungal infection caused by Cryptococcus species, often affecting the lungs and brain, especially in immunocompromised individuals. Histoplasmosis is another fungal infection, caused by the fungus Histoplasma capsulatum. It primarily affects the lungs but can disseminate to other parts of the body. Molluscum contagiosum is a viral skin infection that results in round, firm, painless bumps. These bumps or papules often have a characteristic central umbilication. Verruca vulgaris, commonly known as common warts, is caused by the human papillomavirus (HPV). It typically manifests as rough, cauliflower-like growths.	**Annotation:** Correct<br>**Comment:** None
**Step-by-step Reasoning:** Given the patient's presentation, the physical exam findings, and the provided options:<br>The lesions do not resemble the typical pearly appearance of basal-cell carcinoma. Neither cryptococcosis nor histoplasmosis typically present as dome-shaped papules with central umbilication on the skin. The presentation is consistent with molluscum contagiosum, which often presents as dome-shaped papules with a characteristic central depression or umbilication. While verruca vulgaris is also a viral skin infection, it typically presents as rough growths, which is not the case here.	**Annotation:** Correct<br>**Comment:** None
**Final Answer:** Based on the above, the most likely diagnosis is: D. Molluscum contagiosum.	**Annotation:** Correct

Q89. NEJM Image Challenge 1/27/22

A 40-year-old man with neurofibromatosis type 1 presented for a yearly eye exam and was found to have multiple well-defined, light-brown nodules elevated above the iris bilaterally. What is the most likely diagnosis?

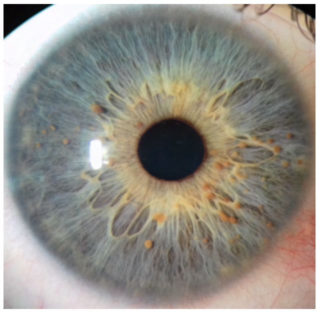

A. Brushfield spots
B. Iris melanoma
C. Lisch nodules
D. Multiple iris nevi
E. Sarcoid-associated uveitis

Correct Answer: Lisch nodules
Explanation: Lisch nodules are melanocytic hamartomas that are common in patients with NF1. Unlike iris nevi, which are flat or minimally elevated with blurred margins, Lisch nodules are elevated above the surface of the iris, have well-defined borders, and occur in multiples. They are not known to result in any ophthalmologic symptoms or complications and no specific treatment is required.

GPT-4V Answer

**Image Comprhension:** The image displays a close-up view of an eye, focusing on the iris. The iris exhibits multiple light-brown, well-defined nodules that appear to be elevated above the iris surface. These nodules are dispersed across the entire span of the iris.	**Annotation:** Correct<br>**Comment:** None
**Recall of Medical Knowledge:** Brushfield spots: These are small, white or light-colored spots often found on the periphery of the iris in individuals with Down syndrome. They are usually associated with light-colored irises and are not typically raised.<br>Iris melanoma: This is a type of cancer that originates from the melanocytes in the iris. It often presents as a pigmented, growing lesion on the iris. It can be singular or multifocal.<br>Lisch nodules: These are benign hamartomas (non-cancerous growths) that appear as small, elevated, dome-shaped, clear or pigmented bumps on the surface of the iris. They are commonly associated with neurofibromatosis type 1 (NF1).<br>Multiple iris nevi: Nevi are benign melanocytic tumors, similar to moles on the skin. An iris nevus is a flat or minimally elevated pigmented spot on the iris.<br>Sarcoid-associated uveitis: Sarcoidosis is a multisystem inflammatory disease that can affect the eyes. Uveitis refers to inflammation of the uvea, which includes the iris, ciliary body, and choroid. Sarcoid-associated uveitis can present with a variety of signs, but not typically with nodules on the iris.	**Annotation:** Incorrect<br>**Comment:** This statement, "Sarcoid-associated uveitis can present with a variety of signs, but not typically with nodules on the iris" is false. Iris nodules are actually a relatively common manifestation of sarcoid-associated uveitis. There are actually named iris nodules (e.g. Koeppe and Busacca nodules) that are frequently tested on boards exams and can be associated with sarcoidosis.
**Step-by-step Reasoning:** Brushfield spots are usually white or light-colored and not typically raised, so option A is unlikely.<br>While the nodules in the image are pigmented, the presence of multiple nodules distributed throughout the iris makes a single malignancy such as iris melanoma (option B) less likely.<br>The patient's history of neurofibromatosis type 1 (NF1) combined with the appearance of multiple raised, light-brown nodules on the iris is consistent with Lisch nodules (option C).<br>Multiple iris nevi (option D) are typically not raised, so this option is less likely.<br>The image doesn't show signs of inflammation that would be consistent with sarcoid-associated uveitis (option E).	**Annotation:** Correct<br>**Comment:** None
**Final Answer:** Conclusion: Based on the patient's history of NF1 and the appearance of the iris in the image, the most likely diagnosis is C. Lisch nodules.	**Annotation:** Correct

Q90. NEJM Image Challenge 1/20/22

A 48-year-old woman with advanced cirrhosis presented with fatigue, jaundice, and abdominal distention. Laboratory studies showed a hemoglobin level of 5.8 g per deciliter (reference range, 12.0 to 15.5), a lactate dehydrogenase level of 1219 U per liter (reference range, 125 to 220), a low haptoglobin level, and an elevated reticulocyte count. Coombs' antiglobulin testing was normal. The result of a peripheral-blood smear is shown. What is the diagnosis?

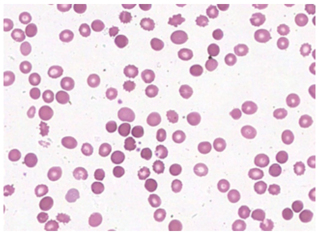

A. Autoimmune hemolytic anemia
B. Babesiosis
C. Hypersplenism
D. Microangiopathic hemolytic anemia
E. Spur-cell hemolytic anemia

Correct Answer: Spur-cell hemolytic anemia
Explanation: The correct answer is spur-cell hemolytic anemia. The peripheral-blood smear as shown demonstrated acanthocytes, or spur cells, with irregularly distributed surface projections and lacking central pallor. Spur-cell hemolytic anemia is a type of nonimmune hemolysis that occurs in patients with advanced liver disease due to excess membrane cholesterol in erythrocytes.

GPT-4V Answer

Image Comprhension: The image appears to be a peripheral-blood smear, which shows cells that are predominantly erythrocytes (red blood cells). A majority of the erythrocytes are of normal morphology, but there are also some abnormally shaped cells. These abnormally shaped erythrocytes have a spiky or irregular contour, giving them a "spur-like" appearance. These specific cell types are termed "spur cells" or "acanthocytes".

Annotation: Correct
Comment: None

Recall of Medical Knowledge: Erythrocytes (red blood cells) normally have a biconcave disc shape. Spur cells or acanthocytes are a type of abnormally shaped erythrocyte characterized by multiple irregular spiky projections. Spur-cell hemolytic anemia is a condition associated with liver disease, especially cirrhosis. It is characterized by the presence of spur cells in the blood. The cholesterol content of the red blood cell membrane is altered in liver disease, leading to the formation of spur cells, which are fragile and undergo hemolysis in the bloodstream. The other options have distinct clinical and laboratory features: Autoimmune hemolytic anemia (AIHA) would usually have a positive Coombs' test (antiglobulin test). Babesiosis is a parasitic infection that would show intraerythrocytic parasites in the blood smear. Hypersplenism results in the sequestration and destruction of blood cells in the spleen but doesn't typically cause a specific change in red cell morphology like spur cells. Microangiopathic hemolytic anemia (MAHA) is characterized by the presence of schistocytes (fragmented red blood cells) on the smear, which is a result of red blood cells passing through damaged small blood vessels.

Annotation: Correct
Comment: None

Step-by-step Reasoning: We observe spur cells in the peripheral-blood smear. The patient has advanced cirrhosis, which is consistent with the etiology for spur-cell hemolytic anemia. The patient's laboratory findings (low hemoglobin, elevated lactate dehydrogenase, low haptoglobin, elevated reticulocyte count) are indicative of hemolysis. Coombs' antiglobulin testing is normal, which rules out autoimmune hemolytic anemia (Option A). No intraerythrocytic parasites are seen, which rules out babesiosis (Option B). There is a specific morphological change (spur cells) seen on the smear, making the generic diagnosis of hypersplenism (Option C) less likely in this context. There are no fragmented red blood cells (schistocytes) observed, which rules out microangiopathic hemolytic anemia (Option D).

Annotation: Correct
Comment: None

Final Answer: Based on the above reasoning, the most appropriate diagnosis for this patient is E. Spur-cell hemolytic anemia.

Annotation: Correct

Q91. NEJM Image Challenge 1/13/22

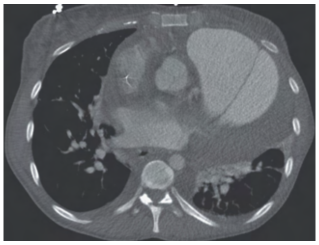

A 35-year-old woman with idiopathic pulmonary arterial hypertension and a pulmonary aneurysm presented with chest pain. Computed tomography (CT) of the chest is shown. What is the most likely diagnosis?

A. Aortic dissection
B. Aortic pseudoaneurysm
C. Intramural hematoma
D. Pulmonary-artery dissection
E. Pulmonary-artery rupture

Correct Answer: Pulmonary-artery dissection
Explanation: The patient was diagnosed with a pulmonary-artery dissection. She was admitted to the hospital, underwent venoarterial extracorporeal membrane oxygenation therapy, and received urgent bilateral lung transplantation with replacement of the main pulmonary artery and pulmonary valve.

GPT-4V Answer

**Image Comprhension:** The provided image appears to be an axial view of a chest CT scan. We can observe the following key features: There is a dilated structure, most likely the pulmonary artery, which is prominently displayed in the middle of the image. Within this dilated structure, there is a distinct luminal line separating it into two compartments. This line is suggestive of a dissection flap. There are no obvious signs of extravasation of contrast outside the vessel, indicating no active bleeding. No evident abnormalities of the aorta or other adjacent structures.	**Annotation:** Correct<br>**Comment:** All correct
**Recall of Medical Knowledge:** Idiopathic pulmonary arterial hypertension (IPAH) can lead to increased pressure in the pulmonary artery. Over time, this can result in dilation and stretching of the vessel walls, which makes them prone to injuries like dissections or ruptures. Aortic dissection involves a tear in the inner layer of the aorta, leading to blood flow between layers and forming a false lumen. It typically presents with sharp, tearing chest pain. Aortic pseudoaneurysm refers to a contained rupture of the aorta with a persistent connection to the true lumen. It's less common than true aneurysms. Intramural hematoma is caused by bleeding within the wall of the aorta without an intimal tear, commonly due to a rupture of the vasa vasorum. Pulmonary-artery dissection involves a tear in the pulmonary artery, leading to a false lumen, similar to an aortic dissection but involving the pulmonary artery instead. Pulmonary-artery rupture is a life-threatening condition where the pulmonary artery wall breaks open, often leading to massive bleeding.	**Annotation:** Correct<br>**Comment:** All correct
**Step-by-step Reasoning:** Given the dilated pulmonary artery and the clear luminal line separating it into two compartments, this is suggestive of a dissection within the pulmonary artery. The absence of obvious extravasation of contrast rules out active bleeding, making pulmonary-artery rupture less likely. The aorta appears unremarkable, making aortic dissection, aortic pseudoaneurysm, and intramural hematoma less likely.	**Annotation:** Correct<br>**Comment:** All correct
**Final Answer:** Based on the image comprehension and the recalled medical knowledge, the most likely diagnosis for the findings in the CT scan is: D. Pulmonary-artery dissection.	**Annotation:** Correct

Q92. NEJM Image Challenge 1/6/22

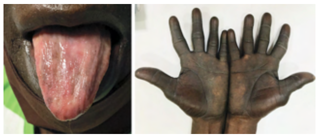

A 59-year-old tile layer presented with fatigue and one year of progressive skin darkening on his palms and soles. Physical examination showed a smooth tongue with patchy areas of mucosal darkening, as well as hyperpigmented palms and soles. Laboratory studies showed a hemoglobin level of 9.4 g per deciliter (reference range, 14 to 18), a mean corpuscular volume of 117 femtoliters (reference range, 80 to 94), and mild leukopenia and thrombocytopenia. Which of the following is the most likely diagnosis?

A. Adrenal insufficiency
B. Contact dermatitis
C. Hyperthyroidism
D. Melanoma
E. Vitamin B12 deficiency

Correct Answer: Vitamin B12 deficiency
Explanation: The patient's vitamin B12 level was 40 pg per milliliter (reference range, 200 to 1100) and a serum intrinsic factor antibody was greater than 200 reference units per milliliter (reference value, <18), consistent with a diagnosis of vitamin B12 deficiency due to autoimmune gastritis. Skin hyperpigmentation occurs in vitamin B12 deficiency due to increased melanin synthesis and is seen more frequently in individuals with darker skin. The hyperpigmentation typically reverses with treatment, as did this patient's.

GPT-4V Answer

Image Comprhension: The image displays two distinct parts:
A close-up of a tongue showing patchy areas of mucosal darkening. The tongue appears smooth, and there are visible areas where the coloration is darker than the typical pinkish hue of a healthy tongue. Two hands, palms up, displaying hyperpigmentation. The skin on the palms appears significantly darker than what would be typically expected.

Annotation: Correct
Comment: None

Recall of Medical Knowledge: Adrenal insufficiency: This condition, also known as Addison's disease, can lead to symptoms like fatigue, weight loss, and skin hyperpigmentation, especially in the folds, palms, and oral mucosa. It happens due to a decreased production of cortisol and aldosterone. Contact dermatitis: This is a localized reaction caused by contact with an irritant or allergen. It usually presents with redness, itching, and inflammation. Chronic exposure can lead to thickening of the skin but not typically hyperpigmentation. Hyperthyroidism: While it affects metabolism and can cause symptoms such as weight loss, sweating, and anxiety, it doesn't usually lead to hyperpigmentation. Melanoma: It's a type of skin cancer, and while it can lead to darkened patches of skin, it doesn't typically present as generalized hyperpigmentation on palms and soles. Vitamin B12 deficiency: A deficiency in vitamin B12 can lead to pernicious anemia. Clinical features include fatigue, pale or jaundiced skin, and sometimes neurological symptoms. It is also associated with a smooth tongue and hyperpigmentation, especially in people with darker skin tones. This deficiency can cause a rise in mean corpuscular volume (MCV) and can sometimes lead to leukopenia and thrombocytopenia.

Annotation: Correct
Comment: None

Step-by-step Reasoning: The patient presents with fatigue, which is common in both adrenal insufficiency and vitamin B12 deficiency. The specific presentation of a smooth tongue with patchy areas of mucosal darkening and hyperpigmented palms and soles narrows our differential. The lab studies further specify the condition: The raised MCV indicates macrocytosis, which is characteristic of vitamin B12 deficiency. Leukopenia and thrombocytopenia are also consistent with vitamin B12 deficiency. Given the combination of clinical presentation, image findings, and laboratory results, the most likely diagnosis is Vitamin B12 deficiency.

Annotation: Correct
Comment: None

Final Answer: Answer: E.Vitamin B12 deficiency.

Annotation: Correct

Q93. NEJM Image Challenge 12/30/21

A 48-year-old woman presented to the emergency department with a 1-week history of fatigue and painful, swollen gums. On examination, she was febrile and tachycardic with a well-demarcated region of gingival infiltration and whitening and submandibular lymphadenopathy. A white-cell count was 225,000 per cubic millimeter. What is the diagnosis?

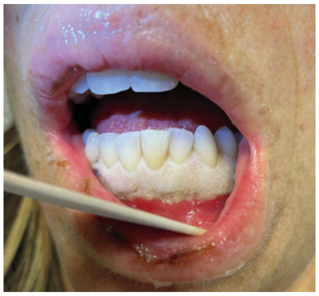

A. Gingival candidiasis
B. Leukemic infiltration of the gingiva
C. Oral lichen planus
D. Proliferative verrucous leukoplakia
E. White sponge nevus

Correct Answer: Leukemic infiltration of the gingiva
Explanation: The correct answer is leukemic infiltration of the gingiva. A bone marrow biopsy showed 89

GPT-4V Answer

**Image Comprhension:** The image shows a close-up view of an individual's open mouth, focusing on the lower gum area. The gums appear swollen, reddened, and infiltrated. There is a noticeable white coloration on the gums, particularly around the teeth, which appears abnormal. Additionally, there seems to be some frothy saliva present, and a foreign object (possibly a swab or stick) is in contact with the lower gum. No other lesions or abnormalities can be observed within the provided view of the mouth.	**Annotation:** Correct<br>**Comment:** None
**Recall of Medical Knowledge:** Gingival candidiasis: Typically presents with white patches on the oral mucosa that can be wiped off, often accompanied by redness and inflammation. Leukemic infiltration of the gingiva: Acute leukemia can manifest with leukemic cell infiltration into the gingiva, leading to gingival enlargement and discoloration. A very high white-cell count, such as the one mentioned, is supportive of a hematologic malignancy. Oral lichen planus: Presents with white, lacy patches on the oral mucosa, often accompanied by redness and inflammation. It doesn't usually present with the extreme swelling seen in the image. Proliferative verrucous leukoplakia: A progressive form of leukoplakia that has a high potential for malignancy. It presents as thick white patches that can't be scraped off. White sponge nevus: A genetic condition causing thick, white, foldable patches of oral mucosa. It's not associated with pain, swelling, or a high white-cell count.	**Annotation:** Correct<br>**Comment:** None
**Step-by-step Reasoning:** Given the symptoms presented:<br>A 1-week history of fatigue: This is a nonspecific symptom but can be seen in hematologic malignancies. Painful, swollen gums: This can be consistent with leukemic infiltration or other conditions. Febrile and tachycardic: Systemic symptoms which might suggest an acute, systemic condition. Well-demarcated region of gingival infiltration and whitening: The image supports this, showing swollen, infiltrated gums with abnormal white patches. Submandibular lymphadenopathy: Can be seen in various conditions including infections and malignancies. A white-cell count of 225,000 per cubic millimeter: This is significantly elevated and strongly suggestive of a hematologic malignancy. Given the clinical presentation and the image, the most likely diagnosis is leukemic infiltration of the gingiva. While other conditions like candidiasis or lichen planus can cause white patches in the mouth, the extremely high white-cell count and systemic symptoms set leukemia apart.	**Annotation:** Correct<br>**Comment:** None
**Final Answer:** Answer: B. Leukemic infiltration of the gingiva.	**Annotation:** Correct

Q94. NEJM Image Challenge 12/23/21

A 65-year-old man presented to the dermatology clinic with a 7-month history of painless skin thickening over the left side of his chest and on his left arm. Physical examination showed sclerotic skin over his left chest and nipple, multiple erythematous nodules over the left arm, axilla, and posterior trunk, left axillary adenopathy, and left arm lymphedema. A skin biopsy showed metastatic carcinoma suggestive of primary breast cancer. What is the diagnosis?

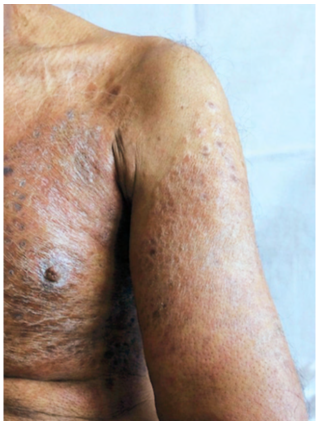

A. Carcinoma en cuirasse
B. Carcinoma erysipeloides
C. Inflammatory breast cancer
D. Morphea
E. Paget's disease

Correct Answer: Carcinoma en cuirasse
Explanation: The patient's skin findings were attributed to carcinoma en cuirasse, a rare form of cutaneous breast cancer metastasis that results in extensive fibrosis of the skin and subcutaneous tissue of the chest wall. The name derives from the resemblance of the condition to a breastplate of armor. The patient was referred to the oncology clinic, and palliative chemotherapy was started.

GPT-4V Answer

**Image Comprhension:** The image showcases a close-up view of an older individual's chest and arm. The skin on the chest and arm appears thickened with patches of discoloration. There are noticeable erythematous (reddish) nodules spread across the left arm and what seems to be the left chest area. The skin also exhibits sclerotic changes, giving it a hardened appearance. In addition, there's evident swelling, or lymphedema, of the left arm.	**Annotation:** Correct<br>**Comment:** None
**Recall of Medical Knowledge:** Carcinoma en cuirasse: A rare form of cutaneous metastasis from breast cancer. It manifests as hardened, sclerotic skin, resembling the cuirass (a piece of armor).<br>Carcinoma erysipeloides: Presents as an erythematous, inflamed patch of skin that looks like cellulitis (a bacterial skin infection). It's another form of cutaneous metastasis from underlying carcinoma, especially breast cancer.<br>Inflammatory breast cancer: A rare and aggressive form of breast cancer that presents with skin changes making the breast appear inflamed - red, warm, and swollen. The skin may also have an 'orange peel' texture.<br>Morphea: A localized form of scleroderma which leads to patches of hard, sclerotic skin, but it's not associated with internal organ involvement or malignancy.<br>Paget's disease: A rare type of cancer involving the nipple and areola. It presents with eczematous changes, itching, burning, and sometimes discharge from the nipple.	**Annotation:** Correct<br>**Comment:** None
**Step-by-step Reasoning:** The presentation of sclerotic skin, especially over the left chest and nipple, combined with erythematous nodules on the left arm and axillary adenopathy, is highly suggestive of metastatic spread of an underlying malignancy.<br>The skin changes described, especially the hardened, sclerotic skin over the chest (cuirass-like appearance), aligns closely with the presentation of Carcinoma en cuirasse.<br>The erythematous nodules could be mistaken for Carcinoma erysipeloides, but the more defining feature of the cuirass-like appearance of the skin shifts the diagnosis towards Carcinoma en cuirasse.<br>While Inflammatory breast cancer can cause skin changes, it typically manifests as an inflamed breast with 'orange peel' texture rather than the hardened, sclerotic skin and nodules seen here.<br>Morphea can cause sclerotic skin but would not explain the nodules, lymphedema, or axillary adenopathy, which are signs of metastatic spread.<br>Paget's disease affects the nipple and areola with eczematous changes, which is not consistent with the image or the described presentation.	**Annotation:** Correct<br>**Comment:** None
**Final Answer:** Based on the above reasoning, the diagnosis is most consistent with: A. Carcinoma en cuirasse.	**Annotation:** Correct

Q95. NEJM Image Challenge 12/16/21

A 37-year-old man presented to the neurosurgery clinic with blurry vision, headache, and difficulty looking upward. On exam, he had diplopia, upward gaze palsy, and vertical misalignment of the eyes. His pupils only minimally constricted to light but constricted to near objects. What is the name of this pupillary exam finding?

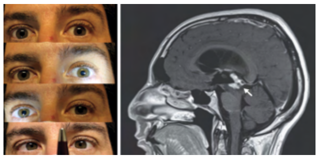

A. Adie’s pupil
B. Anisocoria
C. Argyll Robertson pupil
D. Light-near dissociation
E. Relative afferent pupillary defect (e.g., Marcus Gunn pupil)

Correct Answer: Light-near dissociation
Explanation: Magnetic resonance imaging of the patient’s brain showed an enhancing dorsal midbrain lesion; biopsy later confirmed nonsecreting germinoma. Pupillary light-near dissociation is characterized by the absence of constriction to light with an intact accommodation reflex. Causes of light-near dissociation include dorsal rostral midbrain lesions, as in this case, and neurosyphilis, though the Argyll Robertson pupils associated with syphilis are also typically miotic and irregular at baseline.

GPT-4V Answer

Image Comprhension: The image consists of two parts. On the left side, there are multiple close-up photographs of a person’s eyes. In these photographs, the pupils show variations in constriction and dilation. The pupils appear to be different in size in some images, and in others, the eye appears to have difficulty looking in certain directions, particularly upwards. The images depict the clinical findings mentioned: blurry vision, difficulty in upward gaze, and possibly the pupillary response to light and near objects.
On the right side, there is an MRI scan of what appears to be a sagittal section of a head. There is an arrow pointing to a specific region within the brain, suggesting some kind of lesion or abnormality in that area.

Annotation: Incorrect
Comment: The key to the image on the left is the lack of constriction to light but constriction to near objects. GPT referred to the vignette, but failed to mention where the text explicitly described the findings: "His pupils only minimally constricted to light but constricted to near objects." The interpretation of the left images incorrectly claim to depict: 1. Blurry vision - this is a subjective complaint and cannot be demonstrated on image unless it shows us a blurry image. 2. Difficulty with upward gaze - None of the images demonstrate that the subject is attempting upgaze; 3. GPT states POSSIBLY the pupillary response to light and near objects - This does not describe a finding at all, whether it is abnormal or not. Regarding the right image (the saggital view of the brain MRI, iit is correct that is it a sagittal section of the head, although we typically say MRI’s are of the brain. It is correct there is an arrow pointing to a specific region of the brain, but it does not state what the location is. And it says the arrow suggests there is some kind of lesion or abnormality in that (undefined) region. There is, in fact, an enhancing lesion in the dorsal midbrain.

**Recall of Medical Knowledge:** Adie's pupil: A neurological disorder characterized by a tonically dilated pupil that reacts slowly to light but shows a more normal response to accommodation (i.e., looking at near objects). Anisocoria: A condition characterized by an unequal size of the eye's pupils. Argyll Robertson pupil: Pupils that are small, irregular in shape, and bilaterally constricted. They do not react to direct light but constrict with accommodation. Light-near dissociation: The pupils do not react or react minimally to direct light but constrict when the eye focuses on a near object. This is seen in conditions such as neurosyphilis and third nerve palsy, among others. Relative afferent pupillary defect (Marcus Gunn pupil): A condition in which the direct light reflex is decreased or absent in one eye when a light is shone into it, while the consensual reflex (response of the other eye) is normal.	**Annotation:** Partially correct<br>**Comment:** Its description of Relative afferent pupillary defect is not correct. It is close, but it should be that when light is shown in the affected eye, there is no or slight constriction of both pupils. When shown in the unaffected eye, there is normal constriction of both pupils. So when performing the swinging lamp test, the pupils will appear to dilate when swinging the light to the affected side and constrict when swinging the light to the unaffected side.
**Step-by-step Reasoning:** From the image and description, the person's pupils only minimally constrict to light but constrict to near objects. This pattern of response is consistent with light-near dissociation. Given the other symptoms, including difficulty looking upward and vertical misalignment, it suggests a pathology that could be associated with third nerve palsy or other neurological issues. The MRI might show a lesion or abnormality causing these symptoms, but the specific clinical findings regarding the pupils align with light-near dissociation.	**Annotation:** Partially correct<br>**Comment:** It correctly interpreted the pupillary findings which was explicitly described in the text. Yes, this represents light near dissociation. The trick with this question is that Argyll Robertson Pupil is also a form of light near dissociation and one that many clinicians associate with the clinical findings. However, it is specific to an etiology of neurosyphilis. However this imaging finding showing a dorsal midbrain enhancing lesion can also cause the same pupilary findings (light near dissociation) as Argyll Robertson pupil, but when associated with a lesion due to something other than neurosyphilis, which in this case we can assum is due to a mass lesion, it is referred to as the more broad light-near dissociation. GPT was still uncertain about the presence of a lesion causing these symptoms.
**Final Answer:** Answer: D. Light-near dissociation.	**Annotation:** Correct

Q96. NEJM Image Challenge 12/9/21

A 74-year-old woman presented with a 3-day history of headaches and tongue swelling and several hours of blindness in the right eye. On examination, the tongue was discolored and ulcerated. What is the diagnosis?

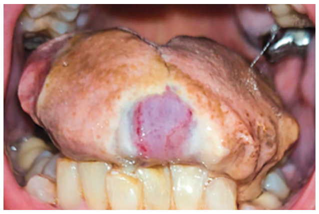

A. Carcinoma of the tongue
B. Carotid artery stenosis
C. Giant-cell arteritis
D. Oral lichen planus
E. Thromboembolic disease

Correct Answer: Giant-cell arteritis
Explanation: The correct answer is giant-cell arteritis. Lingual infarction is a rare complication of giant-cell arteritis. Her vision and tongue necrosis improved with high-dose glucocorticoids and tocilizumab.

GPT-4V Answer	
**Image Comprhension:** The image depicts an up-close view of a person's tongue. The tongue appears swollen with discoloration that is unevenly distributed. Notably, there is a prominent ulcerated region that is reddish-purple in color, surrounded by areas of paler discoloration. This particular ulceration stands out due to its size and distinct coloration. Additionally, there's a dental instrument present, suggesting the image was taken during a clinical examination.	**Annotation:** Correct<br>**Comment:** All correct
**Recall of Medical Knowledge:** Carcinoma of the tongue: This refers to a malignant tumor of the tongue. Signs include persistent tongue pain, a lump or sore on the tongue that doesn't heal, and difficulty chewing or swallowing. Ulceration can be a feature of tongue cancer. Carotid artery stenosis: This involves narrowing of the carotid arteries, the primary arteries supplying blood to the brain. Symptoms might include sudden numbness or weakness, difficulty speaking, vision problems, and dizziness. However, it does not typically cause tongue ulceration. Giant-cell arteritis (GCA): An inflammatory condition affecting the lining of your arteries, especially the arteries in the temples. Symptoms include headaches, jaw pain, vision problems, and sometimes tongue pain. If left untreated, GCA can lead to blindness. Oral lichen planus: This is an inflammatory condition that can affect the mucous membranes inside the mouth. Oral lichen planus can cause white patches, redness, and ulcers. Thromboembolic disease: This refers to a condition where blood clots form in the bloodstream and block blood flow. Depending on the location, it could lead to various symptoms. While it might explain the blindness if a clot were to affect the blood supply to the eye, it doesn't directly correlate with tongue symptoms.	**Annotation:** Correct<br>**Comment:** All correct
**Step-by-step Reasoning:** Given the patient's presentation with a 3-day history of headaches, tongue swelling, and several hours of blindness in the right eye, and considering the image of the ulcerated and discolored tongue, we can proceed with the following reasoning:<br>The ulceration on the tongue doesn't immediately point to carotid artery stenosis or thromboembolic disease as the primary cause. Carcinoma of the tongue could account for the ulceration, but it doesn't explain the sudden blindness or the headaches. Oral lichen planus could potentially account for tongue symptoms but wouldn't necessarily explain the blindness. Giant-cell arteritis (GCA), on the other hand, can cause headaches, vision problems (including blindness), and tongue pain or discoloration due to inflammation of the arteries. In severe cases, the reduced blood flow could cause tongue ulceration.	**Annotation:** Correct<br>**Comment:** All correct
**Final Answer:** Given the combination of symptoms and the image, the most fitting diagnosis would be: C. Giant-cell arteritis.	**Annotation:** Correct

Q97. NEJM Image Challenge 12/2/21

A female infant delivered at term had a “blueberry muffin” rash at birth. Laboratory tests and imaging studies were normal. Skin biopsy showed a dense infiltrate of cells with kidney-shaped nuclei and positive S100+ and CD1a+ on immunohistochemistry. What is the most likely diagnosis?

A. Congenital cytomegalovirus infection
B. Congenital rubella syndrome
C. Langerhans-cell histiocytosis
D. Leukemia cutis
E. Transient myeloproliferative disorder of Down syndrome

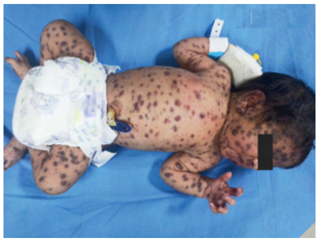

Correct Answer: Langerhans-cell histiocytosis
Explanation: The patient was diagnosed with congenital Langerhans-cell histiocytosis. Because of the lack of extracutaneous involvement, the lesions were expected to resolve without treatment. At follow-up 6 weeks later, the skin lesions had resolved. The infant continues to be followed for signs of recurrence.

GPT-4V Answer

Image Comprhension: The image shows a female infant with widespread purplish-red papular lesions distributed all over her body, resembling the “blueberry muffin” appearance. The baby’s face and body are peppered with these spots, and there are no apparent abnormalities other than the skin lesions. The presence of a diaper and medical paraphernalia indicates a clinical setting.

Annotation: Correct
Comment: None

Recall of Medical Knowledge: "Blueberry muffin" rash is a term used in pediatrics to describe a newborn with multiple purpuric or erythematous macules, nodules, or papules. The differential diagnosis for a "blueberry muffin" rash includes:
Congenital infections like rubella, cytomegalovirus (CMV), and toxoplasmosis. Hematologic disorders such as leukemia cutis and transient myeloproliferative disorder of Down syndrome. Langerhans-cell histiocytosis (LCH). The cells with kidney-shaped nuclei described from the biopsy and their positive staining for S100 and CD1a are indicative of Langerhans cells. Langerhans cells are a type of dendritic cell residing in the skin.
S100 is a protein marker often positive in various cells including melanocytes, Schwann cells, and Langerhans cells. CD1a is specifically expressed in Langerhans cells.

Annotation: Correct
Comment: None

Step-by-step Reasoning: The description of the skin biopsy showing cells with kidney-shaped nuclei is a histological feature suggestive of Langerhans cells. Immunohistochemistry showing positive S100 and CD1a further supports the diagnosis as these markers are indicative of Langerhans cells. Based on the aforementioned points, the skin findings and histological features are consistent with Langerhans-cell histiocytosis (LCH). While other options like congenital infections or hematologic disorders could give rise to a "blueberry muffin" rash, the skin biopsy findings described here are specific to LCH and not seen in the other listed conditions.

Annotation: Correct
Comment: None

Final Answer: Answer: C. Langerhans-cell histiocytosis.

Annotation: Correct

Q98. NEJM Image Challenge 11/25/21

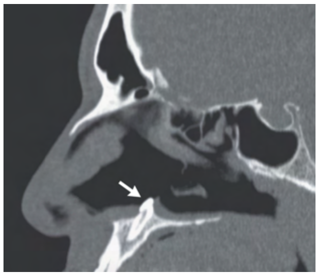

A 38-year-old man presented to the otolaryngology clinic with chronic difficulty breathing through his right nostril. Physical examination showed nasal septal deviation, calcified septal spurs, and a 2-cm perforation in the posterior septum. On rhinoscopy, a hard, nontender, white mass was observed in the floor of the right nostril. CT of the paranasal sinuses showed a well-defined, radiodense mass. Which of the following is the most likely etiology of the nasal mass?

A. Calcified polyp
B. Enchondroma
C. Osteoma
D. Rhinolith
E. Tooth

Correct Answer: Tooth
Explanation: The radiodense mass was consistent with an inverted ectopic tooth in the nasal cavity and was thought to explain the patient's obstructive symptoms. After surgical removal of the ectopic tooth, the patient's symptoms resolved.

GPT-4V Answer

**Image Comprhension:** The image appears to be a CT scan of the paranasal sinuses. The primary feature of interest is a well-defined, radiodense mass located in the floor of the right nostril, as indicated by the arrow. This radiodense mass is consistent with a calcified or bony structure.	**Annotation:** Correct<br>**Comment:** None
**Recall of Medical Knowledge:** Calcified polyp: Polyps are soft growths arising from the mucous membranes and are usually not calcified. However, over time, chronic inflammation can lead to calcification of polyps, making them radiodense. Enchondroma: Enchondromas are benign tumors of cartilage origin. They are usually found in bones and are uncommon in the nasal cavity. Osteoma: Osteomas are benign bony growths commonly found in the paranasal sinuses, especially the frontal sinus. They are slow-growing and can become quite large. Rhinolith: Rhinoliths are calcified masses that form around a nidus, such as a piece of cotton, dried nasal secretion, or other foreign body, left in the nasal cavity for an extended period. Rhinoliths can be observed as radiodense masses on imaging. Tooth: Ectopic teeth can occasionally be found in the nasal cavity, but they are extremely rare. These are usually supernumerary teeth that have migrated or developed outside their usual location.	**Annotation:** Correct<br>**Comment:** None
**Step-by-step Reasoning:** The nasal mass in the image is clearly radiodense, making it likely to be composed of bony or calcified material. Given the clinical presentation and the findings on the CT scan, an enchondroma or ectopic tooth seems less likely. Enchondromas are rare in the nasal cavity, and an ectopic tooth in the nasal cavity is exceptionally rare. A calcified polyp could be a possibility, but the description does not indicate a long-standing inflammatory process or other reasons for polyp formation. Osteoma could be a possibility, but the mass's location in the floor of the nostril makes it less typical for osteoma. Rhinoliths, however, form around foreign bodies left in the nasal cavity, and their presentation and appearance on imaging can match the findings described.	**Annotation:** Partially correct<br>**Comment:** Partially correct reasoning - ectopic tooth is extremely rare but the shape and presentation of the radiodense nasal mass is consistent with a tooth.
**Final Answer:** Based on the provided information and the CT scan image, the most likely etiology of the nasal mass is: D. Rhinolith.	**Annotation:** Incorrect

Q99. NEJM Image Challenge 11/18/21

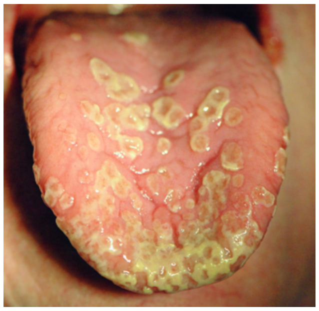

A 50-year-old woman with a history of Crohn's disease presented with 10 days of tongue and inner cheek pain. Laboratory studies showed an absolute eosinophil count of 870 per cubic millimeter (reference range, 50 to 500). What is the most likely diagnosis?

A. Herpes simplex gingivostomatitis
B. Herpetic geometric glossitis
C. Lichen planus
D. Oral candidiasis
E. Pyostomatitis vegetans

Correct Answer: Pyostomatitis vegetans
Explanation: A biopsy specimen of the dorsal tongue showed intraepithelial microabscesses with neutrophils and eosinophils, findings consistent with pyostomatitis vegetans. The patient was referred for gastroenterologic evaluation, and her oral lesions were treated with topical glucocorticoids and benzocaine. Pyostomatitis vegetans is a rare oral mucosal condition that is most often associated with inflammatory bowel disease.

GPT-4V Answer

**Image Comprhension:** The image depicts a close-up view of a tongue. The tongue exhibits multiple raised, irregular, yellowish-white plaques or lesions, which are distributed in a patchy manner. The surrounding tongue tissue appears inflamed and reddened.	**Annotation:** Correct<br>**Comment:** None
**Recall of Medical Knowledge:** Herpes simplex gingivostomatitis: Often presents with painful oral ulcers and can be associated with fever and malaise. The lesions usually start as small vesicles which rupture to form painful ulcers. Herpetic geometric glossitis: A variant of herpes simplex infection specific to the tongue, characterized by a geometric pattern of ulcers or erosions on the dorsum of the tongue. Lichen planus: A chronic inflammatory condition that can affect the skin and mucous membranes. In the oral cavity, it often presents as white, lacy streaks known as Wickham's striae. It may be associated with painful ulcers. Oral candidiasis (Thrush): Caused by the Candida fungus, it presents as creamy white lesions on the tongue or inner cheeks. It can be scraped off to reveal a red base. Pyostomatitis vegetans: A rare oral manifestation of inflammatory bowel disease, especially Crohn's disease. It is characterized by multiple pustules and yellowish, snail-track erosions in the mouth. It's often associated with an elevated eosinophil count.	**Annotation:** Correct<br>**Comment:** None
**Step-by-step Reasoning:** The description of the patient having a history of Crohn's disease and an elevated absolute eosinophil count aligns with the characteristics of pyostomatitis vegetans. The appearance of the tongue with raised, irregular, yellowish-white plaques is also consistent with pyostomatitis vegetans. The other options (A, B, C, and D) are less likely given the patient's history and the specific appearance of the lesions: While herpes simplex gingivostomatitis and herpetic geometric glossitis are both painful, their typical presentation does not align perfectly with the image or the clinical details provided. Lichen planus usually presents with white, lacy streaks and not the kind of lesions seen in the image. Oral candidiasis would present with creamy white lesions that can be scraped off, which is not described in this case.	**Annotation:** Correct<br>**Comment:** None
**Final Answer:** Conclusion: The most likely diagnosis is E. Pyostomatitis vegetans.	**Annotation:** Correct

Q100. NEJM Image Challenge 11/11/21

A 61-year-old woman presented with discoloration along her gums that had rapidly expanded over the past year. What is the diagnosis?

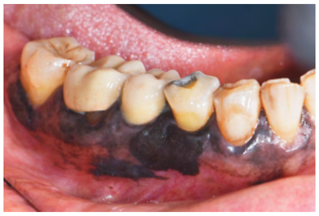

A. Amalgam tattoo
B. Gingival melanoma
C. Kaposi's sarcoma
D. Oral melanoacanthoma
E. Physiologic pigmentation

Correct Answer: Gingival melanoma
Explanation: The correct answer is gingival melanoma. Melanomas may arise from the mucosal epithelial lining of the oral cavity and gastrointestinal tract. The patient was referred to the oncology clinic and underwent imaging, which was negative for lymph-node involvement or distant metastases. She subsequently underwent surgical resection of the lesion and had no recurrence at four-month follow up.

GPT-4V Answer

**Image Comprhension:** The image depicts a close-up view of the inside of a mouth, specifically focusing on the lower gums. The teeth appear to be in relatively good condition. There is significant discoloration present on the gums. The discoloration is dark, almost black or deep purple, and seems to be concentrated in irregular patches, mainly towards the base of the teeth. The rest of the gums, especially towards the bottom of the image, appears pink and healthy.	**Annotation:** Correct<br>**Comment:** None
**Recall of Medical Knowledge:** Amalgam Tattoo: This is a blue-gray discoloration of the oral mucosa, resulting from the implantation of amalgam particles during dental procedures. It's a benign lesion. Gingival Melanoma: A rare type of oral malignant melanoma that originates from the melanocytes of the gingiva. It can appear as a dark pigmented lesion. Kaposi's Sarcoma: It is a cancer that develops from the cells lining lymph or blood vessels. It usually appears as purple, red, or brown blotches or tumors on the skin or inside the mouth. Oral Melanoacanthoma: It's a rare, benign pigmented lesion mostly seen in the buccal mucosa. It presents as a brown-black macule. Physiologic Pigmentation: This is the normal variation of gum color in some individuals, especially those with darker skin. It's benign and generally doesn't change over time.	**Annotation:** Correct<br>**Comment:** None
**Step-by-step Reasoning:** The discoloration observed is dark, which is consistent with a few of the mentioned conditions. The rapidly expanding growth over a year could be suggestive of malignancy. The discoloration does not seem to be uniformly spread as what would be expected from physiologic pigmentation. Amalgam tattoos are usually more localized and often linked to a previous dental procedure where amalgam might have been embedded in the soft tissues. In this image, the discoloration is more diffuse and widespread. Kaposi's Sarcoma usually appears as red, purple, or brown blotches. The appearance in the image is dark but not distinctly red or purple, so it might not be the most likely diagnosis, although not entirely ruled out. Oral Melanoacanthoma usually presents as a brown-black macule, but its rapid expansion over a year makes it less likely. Gingival Melanoma appears as a dark pigmented lesion, and given its malignancy, it can exhibit rapid growth.	**Annotation:** Correct<br>**Comment:** None
**Final Answer:** Based on the above reasoning, the most likely diagnosis seems to be B. Gingival Melanoma.	**Annotation:** Correct
